# Upregulation of LINC02154 promotes esophageal cancer progression by enhancing cell cycling and epithelial-mesenchymal transition

**DOI:** 10.1016/j.ncrna.2025.06.001

**Published:** 2025-06-02

**Authors:** Kotoha Shimote, Takeshi Niinuma, Hiroshi Kitajima, Kazuya Ishiguro, Eiichiro Yamamoto, Gota Sudo, Akira Yorozu, Mutsumi Toyota, Masahiro Kai, Masashi Idogawa, Hiromu Suzuki

**Affiliations:** a Division of Molecular Biology, Department of Biochemistry, Sapporo Medical University School of Medicine, Sapporo, Japan; b Department of Gastroenterology and Hepatology, Sapporo Medical University School of Medicine, Sapporo, Japan; c Division of Medical Genome Sciences, Department of Genomic and Preventive Medicine, Sapporo Medical University School of Medicine, Sapporo, Japan

**Keywords:** Esophageal cancer, ESCA, lncRNA, Cell cycle, miR-200b, VIM

## Abstract

Long noncoding RNAs (lncRNAs) play crucial roles in the progression of human malignancies; however, their involvement in esophageal cancer (ESCA) remains incompletely understood. In this study, we screened for lncRNAs upregulated in ESCA and identified 12 lncRNAs significantly upregulated in primary ESCA tumors. Among those, elevated LINC02154 expression correlated positively with advanced T stages. LINC02154 knockdown in ESCA cell lines suppressed cell proliferation and migration, while ectopic expression of LINC02154 enhanced colony formation. Depletion of LINC02154 suppressed genes involved in various oncogenic processes, including cell cycling, epithelial-mesenchymal transition (EMT), and metabolism. We also found that LINC02154 promotes EMT and enhances chemoresistance, at least in part, through suppression of miR-200b. Finally, RNA-pulldown and mass spectrometry analysis revealed that LINC02154 interacts with proteins involved in the cornified envelope or desmosome. These findings suggest that LINC02154 exerts oncogenic effects through modulation of multiple oncogenic signaling pathways in ESCA and that LINC02154 is a potential therapeutic target.

## Graphical abstract

**Figure:**
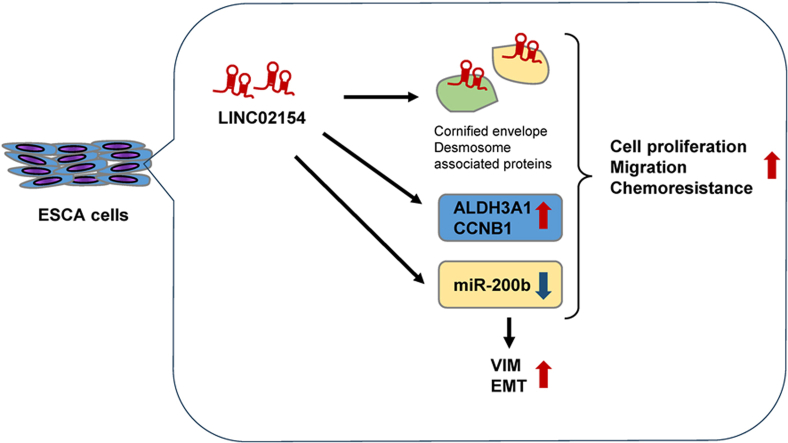

## 1 Introduction

Esophageal cancer (ESCA) is a deadly malignancy that ranked as the seventh leading cause of cancer-related mortality in 2022 [1]. There are two predominant histological subtypes of ESCA, esophageal squamous cell carcinoma (ESCC) and esophageal adenocarcinoma (EAC). ESCC is prevalent in East Asia and Africa, where it is strongly associated with such risk factors as tobacco smoking and alcohol consumption, while EAC is more common in Western countries, where it is linked to gastroesophageal reflux disease and obesity [2]. Despite recent advances in treatment, including improvements in surgical techniques, chemotherapy, immunotherapy, and radiotherapy, the overall 5-year survival rate for ESCA remains below 20 %, primarily due to late-stage diagnosis and the high incidence of metastasis [3]. Consequently, there is an urgent need to identify novel therapeutic targets in ESCA.

Long noncoding RNAs (lncRNAs) are a class of RNA molecules exceeding 200 nt in length that, unlike mRNAs, do not encode proteins. Despite their noncoding nature, lncRNAs play crucial roles in regulating gene expression and are involved in a variety of cellular processes, including chromatin remodeling, transcriptional regulation, and post-transcriptional modification [4]. The functional diversity of lncRNAs is attributed to their ability to interact with DNA, RNA, and proteins, thereby influencing cellular pathways at multiple levels [5]. For example, the lncRNA XIST is essential for X-chromosome inactivation and acts by both coating the X chromosome and recruiting polycomb repressive complex 2 (PRC2) to establish a transcriptionally silent state [6]. Dysregulation of lncRNAs has been implicated in various diseases, particularly cancer [7]. lncRNAs can function as oncogenes or tumor suppressors, and specific lncRNAs have been shown to promote tumorigenesis, metastasis, and resistance to therapy [8]. For instance, the lncRNA HOTAIR contributes to breast cancer metastasis by reprogramming the chromatin state and altering gene expression profiles [9].

Recent studies have increasingly focused on identifying novel biomarkers and therapeutic targets to improve the diagnosis and treatment of ESCA. Among these, lncRNAs have emerged as crucial regulators of gene expression with significant impacts on the biology of cancers, including ESCA [10]. For example, the lncRNA PVT1 is upregulated in ESCC, and its overexpression is associated with lymphatic metastasis, advanced TNM stage, and poorer metastasis-free survival [11]. Conversely, the tumor-suppressive lncRNA MEG3 is epigenetically silenced and downregulated in ESCC [12].

As demonstrated by these studies, lncRNAs play crucial roles in the progression of ESCA. Therefore, we aimed to identify novel ESCA-related lncRNAs that could serve as potential biomarkers or therapeutic targets. We identified overexpression of LINC02154 in primary ESCA tumors and ESCA cell lines, and found that upregulation of LINC02154 contributes to ESCA progression through multiple oncogenic pathways.

## 2 Material and methods

### 2.1 Cell lines, siRNA and miRNA transfection and vector production

The TE4 (RBRC-RCB2097), TE5 (RBRC-RCB1949), TE9 (RBRC-RCB1988) and TE15 (RBRC-RCB1951) cell lines were obtained from the RIKEN BRC Cell Bank (Tsukuba, Ibaraki, Japan). All cell lines were cultured in RPMI-1640 medium supplemented with 10 % fetal bovine serum (FBS). For RNA interference-induced gene knockdown, predesigned Dicer-Substrate Short Interfering RNAs (DsiRNAs) targeting LINC02154 were purchased from Integrated DNA Technologies, Inc. (Coralville, IA, USA). ESCA cells (3 × 10^3^ cells/well in 96-well plates or 1 × 10^5^ cells/well in 12-well plates) were transfected with DsiRNAs (20 nM) or a negative control DsiRNA (20 nM) using Lipofectamine RNAiMAX (Thermo Fisher Scientific, Waltham, MA, USA) according to the manufacturer's instructions. For miRNA transfection, ESCA cells (3 × 10^3^ cells/well in 96-well plates or 1 × 10^5^ cells/well in 12-well plates) were transfected with a miR-200b mimic, miRNA mimic control, miR-200b inhibitor, or inhibitor control (20 nM) using Lipofectamine RNAiMAX (Thermo Fisher Scientific) according to the manufacturer's instructions. The sequences of the siRNAs are listed in Supplementary Table 1. A lentivirus vector expressing LINC02154 was produced as described previously [13]. Cells infected with the LINC02154 vector were selected in culture medium containing 5 μg/mL blasticidin S (Thermo Fisher Scientific) for 1 week.

### 2.2 Cell viability and colony formation assay

ESCA cells were transfected with DsiRNAs as described above. Cell viability assays were then carried out 48 or 72 h after transfection using a Cell Counting kit-8 (Dojindo, Kumamoto, Japan) according to the manufacturer's instructions. For colony formation assays, cells infected with LINC02154 vector (2000 cells/well in 6-well plates) were cultured for 10 days in growth medium. The colonies that developed were stained with Giemsa and measured using ImageJ software (NIH, Bethesda, MD, USA).

### 2.3 Cell cycle assay

TE-5 cells were transfected with DsiRNAs as described above and incubated for 48 h, after which they were treated using a Cell Cycle Assay Solution Blue (Dojindo, Kumamoto, Japan) according to the manufacturer's instructions. Flow cytometric analyses were performed using a BD FACSCanto II (BD Biosciences, Franklin Lakes, NJ, USA) with BD FACSDiva software (BD Biosciences). Cell cycle analysis was performed using FlowJo software (FlowJo, LLC, Ashland, OR, USA).

### 2.4 Drug resistance assays

ESCA cells infected with the LINC02154 expression vector or an empty vector were seeded into 96-well plates (5000 cells/well) and incubated for 24 h. The cells were then treated for 48 h with cisplatin (5 or 10 μg/mL) or 5-fluorouracil (5-FU; 1.25 or 2 μg/mL), after which cell viabilities were assessed using the Cell Counting kit-8 (Dojindo, Kumamoto, Japan) according to the manufacturer's instructions.

### 2.5 Cell migration and invasion assays

Cell migration and invasion assays were performed using transwell chambers as described previously [14]. Briefly, ESCA cells were transfected with DsiRNAs or infected with LINC02154 vector as described above, after which 5 × 10^4^ cells were added to the upper chamber. RPMI-1640 medium supplemented with 10 % FBS was added to the lower chamber. After incubation for 22 h, migrating or invading cells were stained using a Diff quick stain kit (Sysmex, Tokyo, Japan).

### 2.6 RNA extraction and quantitative reverse-transcription PCR

Total RNA was extracted using a FastGene Basic Kit (Nippon Genetics Co., Ltd., Tokyo, Japan) or TRI reagent (Molecular Research Center, Inc., Cincinnati, OH, USA) according to the manufacturers' instructions. Total RNA was extracted from cells transfected with DsiRNA as described above 48 or 72 h after transfection. Total RNA from normal esophageal tissue from a healthy individual was purchased from BioChain (Newark, CA, USA). Nuclear and cytoplasmic RNAs of ESCA cells were isolated using a Cytoplasmic and Nuclear RNA Purification kit (Norgen Biotek, Thorold, Canada). Single-stranded cDNA was prepared using a PrimeScript RT Reagent Kit with gDNA Eraser Perfect Real Time (TaKaRa Bio, Kusatsu, Japan). qRT-PCR was performed using PowerUp SYBR Green Master Mix (Invitrogen by Thermo Fisher Scientific) with a Quant Studio 3 (Applied Biosystems by Thermo Fisher Scientific). Relative expression levels of target genes were determined using an endogenous housekeeping gene beta-2-microglobulin (B2M) as an internal control. Primer sequences are listed in Supplementary Table 2 miRNA expression analysis was performed using TaqMan microRNA assays (Thermo Fisher Scientific) according to the manufacturer's instructions. U6 snRNA (RNU6B; Thermo Fisher Scientific) was used as an endogenous control.

### 2.7 Western blot analysis

ESCA cells were transfected with DsiRNAs or miRNA mimic as described above, and total cell lysates were extracted 72 h after transfection. Western blot analyses were performed as described previously [15]. A mouse monoclonal anti-GAPDH mAb (1:5000 dilution, HRP-60004, Proteintech, Rosemont, IL, USA), rabbit monoclonal anti-cyclin B1 mAb (1:1000 dilution, #12231, Cell Signaling Technology, Danvers, MA, USA), and rabbit recombinant anti-ZEB2 antibody (1:3000 dilution, 82020-1-RR, Proteintech) were used.

### 2.8 RNA sequencing

Total RNA was extracted using the FastGene Basic Kit as described above, after which sequencing libraries were prepared using a NEBNext Poly(A) mRNA Magnetic Isolation Module (NEW ENGLAND BioLabs Inc., Ipswich, MA, USA) and NEBNext Ultra II Directional RNA Library Prep Kit (NEW ENGLAND BioLabs Inc.). Sequencing was then performed using NovaSeq 6000 (Illumina), and mapping and quantification were performed using the STAR-RSEM pipeline. RNA-sequencing (RNA-seq) data were analyzed using GeneSpring GX version 13 (Agilent Technologies, Santa Clara, CA, USA) and Gene Set Enrichment Analysis (GSEA; Broad Institute, Cambridge, MA, USA). The NCBI SRA accession number for the RNA-seq data is PRJNA1219221.

### 2.9 In vitro transcription

DNA templates for LINC02154 and its antisense RNA for in vitro transcription were PCR amplified from a LINC02154 expression vector. RNA labeled with 5-Bromo-UTP (BrU) was synthesized using a CUGA7 in vitro Transcription Kit according to the manufacturer's instructions (NIPPON GENETEC, Tokyo, Japan). Briefly, 0.25 pmol of template DNA was added to a reaction mixture containing T7 polymerase and BrU mix (1.25 mM BrU and 1.25 mM UTP) and incubated for 2 h at 37 °C. The primer sequences are listed in Supplementary Table 2.

### 2.10 RNA pulldown assay and mass spectrometry analysis

RNA pull-down assays were performed using a RiboCluster Profiler RiboTrap Kit (MBL, Tokyo, Japan) according to the manufacturer's instructions. TE-5 cells (1 × 10^8^) were lysed in 1200 μL of lysis buffer and incubated for 10 min at 4 °C, after which 60 μL of detergent solution were added. The lysate was then centrifuged at 3000× *g* for 3 min at 4 °C to precipitate the nuclei. The supernatant representing the cytoplasmic extract was mixed with BrU-labeled LINC021254 sense or anti-sense RNA (100 pmol), after which RNA-protein complexes were immunoprecipitated with BrdU antibody-immobilized Protein G Dynabeads (Thermo Fisher Scientific). Protein immunoprecipitated from TE-5 cells was used for peptide preparation for mass spectrometry analysis, and MS/MS data analysis was performed as described previously [13].

### 2.11 Data analysis

RNA-seq data from primary ESCA tumors in The Cancer Genome Atlas (TCGA) dataset were obtained and analyzed on UCSC Xena (http://xena.ucsc.edu/) [16]. RNA-seq data from ESCA cell lines (SRP186687) and human esophagus epithelial cells (SRP437528 and SRP561033) were obtained from the Sequence Read Archive (https://www.ncbi.nlm.nih.gov/sra), after which mapping and quantification were performed using the STAR-RSEM pipeline. Raw read counts of RNA-seq data from primary ESCC tissues (GSE130078) were obtained from the Sequence Read Archive, after which normalization and quantification were performed using DESeq2. miRNA target prediction in the LINC02154 sequence was performed using miRcode (http://www.mircode.org/) and scanMiR [17,18].

### 2.12 Statistical analysis

Quantitative variables were analyzed using a Student's *t*-test or one-way analysis of variance (ANOVA). Categorical values were compared using Fisher's exact test. Survival analysis was performed using the log-rank test for two-group comparisons. All data were analyzed using EZR version 1.40.

## 3 Results

### 3.1 Upregulation of LINC02154 is associated with tumor progression and the molecular features of ESCA

**Fig. 1:**
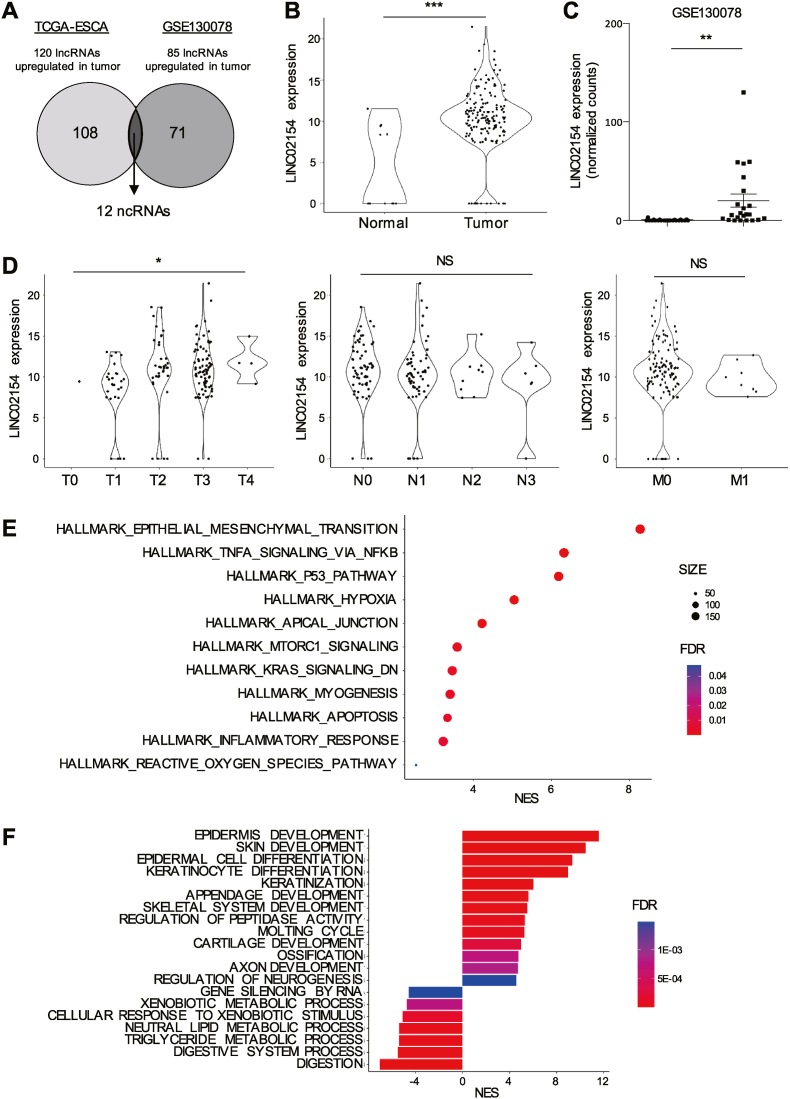
Detection of LINC02154 upregulation in primary ESCA tumors. (A) Venn diagram showing lncRNAs upregulated in primary ESCA tumors in the TCGA-ESCA and GSE130078 datasets. (B) Levels of LINC02154 expression in normal esophageal tissues (n = 11) and primary tumors (n = 160) in TCGA-ESCA dataset. (C) Levels of LINC02154 expression in normal tissues (n = 23) and primary tumors (n = 23) in the GSE130078 dataset. (D) Correlations between levels of LINC02154 expression and those of T-factors (left), N-factors (middle) and M-factors (right) in TCGA-ESCA dataset. (E) Results of GSEA for the indicated gene sets based on genes upregulated in ESCA with high LINC02154 expression. NES, normalized enrichment score; FDR, false discovery rate. (E) Results of GSEA for the indicated ontology gene sets based on genes upregulated in ESCA with high LINC02154 expression. ∗*P* < 0.05, ∗∗*P* < 0.01, ∗∗∗*P* < 0.001, NS, not significant.

Utilizing RNA-seq data from ESCA patients in The Cancer Genome Atlas dataset (TCGA-ESCA; normal, n = 11; tumor, n = 161), we identified 120 lncRNAs upregulated in ESCA tumor tissues (>16-fold, p < 0.00001). In addition, utilizing RNA-seq data from paired samples of tumors and normal tissues from 23 ESCC patients (GSE130078), we identified 83 lncRNAs upregulated in ESCC tumors (>16-fold, p < 0.05). Among the affected lncRNAs, 12 were upregulated in both cohorts (Fig. 1A, Supplementary Table 3). Among those 12, LINC02154 (ENSG00000235385) is reportedly upregulated in various types of tumors, including head and neck squamous cell carcinoma [19,20]. We therefore further analyzed the involvement of LINC02154 expression in the clinicopathological and molecular characteristics of primary ESCA. Upregulated LINC02154 expression was observed in primary tumor tissues in two different ESCA cohorts (Fig. 1B and C). The elevated levels of LINC02154 were associated with more advanced T-stages in primary ESCA, though they were not associated with the N- or M-stages (Fig. 1D). Moreover, although LINC02154 expression was upregulated in both EAC and ESCC as compared to normal esophageal tissues (Supplementary Fig. 1A), when TCGA-ESCA tumors were divided into EACs and ESCCs, the association between the LINC02154 expression level and T stage was not statistically significant, perhaps due to the small numbers of samples in the respective categories (Supplementary Fig. 1B and C). GSEA indicated that genes associated with cancer-related signaling, including epithelial-mesenchymal transition (EMT), TNF-α signaling, and the p53 pathway, were significantly enriched in primary ESCA tumors with high LINC02154 expression (Fig. 1E). In addition, gene ontology (GO) analysis revealed that higher expression of LINC02154 is associated with genes related to development, differentiation, and keratinization, and negatively associated with metabolic processes in primary ESCA (Fig. 1F).

### 3.2 LINC02154 exerts oncogenic effects in ESCA cells

**Fig. 2:**
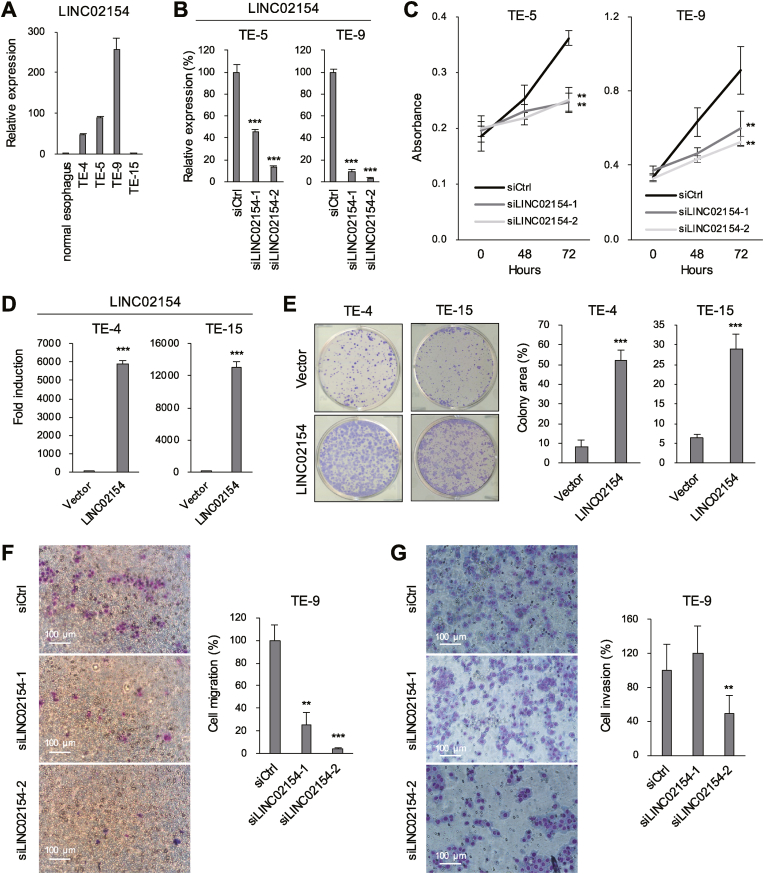
Functional analysis of LINC02154 in ESCA cells. (A) qRT-PCR analysis of LINC02154 expression in normal esophageal tissue and ESCA cell lines. (n = 3). (B) qRT-PCR analysis of LINC02154 expression in ESCA cells transfected with a control siRNA or siRNAs targeting LINC02154. (n = 3). (C) Cell viability assays in ESCA cells transfected with the indicated siRNAs. (n = 6). (D) qRT-PCR analysis of LINC02154 expression in ESCA cells infected with a control vector or a LINC02154 expression vector. (n = 3). (E) Colony formation assays using ESCA cells with or without ectopic LINC02154 expression. Summarized results are shown on the right. (n = 3). (F, G) Cell migration (F) and invasion (G) assays with TE-9 cells transfected with the indicated siRNA. Representative results are shown on the left, summarized results are shown on the right. (n = 3). Error bars represent SDs. ∗∗*P* < 0.01, ∗∗∗*P* < 0.001.

To assess the oncogenicity of LINC02154, we first assessed LINC02154 expression in several ESCA cell lines and detected higher LINC02154 expression in most ESCA cell lines than in normal esophageal tissue (Fig. 2A). Analysis using publicly available RNA-seq data also confirmed that normal esophageal epithelial cells express LINC02154 at limited levels as compared to ESCA cells (Supplementary Fig. 2A). In subsequent knockdown experiments, after confirming that LINC02154 expression was significantly decreased in two ESCA cell lines (TE-5 and TE-9) transfected with a siRNAs targeting LINC02154 (siLINC02154-1 or siLINC02154-2, Fig. 2B), we observed that LINC02154 depletion inhibited ESCA cell proliferation (Fig. 2C). Conversely, ectopic expression of LINC02154 in two ESCA cell lines expressing relatively lower levels of endogenous LINC02154 than TE-5 and TE-9 cells (TE-4 and TE-15, Fig. 2D) promoted colony formation by both cell lines (Fig. 2E). Cell cycle analysis revealed that knockdown of LINC02154 induced G1 arrest in ESCA cells (Supplementary Fig. 2B). Furthermore, knockdown of LINC02154 suppressed the migratory and invasive ability of ESCA cells (Fig. 2F and G). In contrast, overexpression of LINC02154 significantly enhanced the invasive ability of ESCA cells (Supplementary Fig. 2C and D).

### 3.3 Effects of LINC02154 knockdown on gene expression profiles in ESCA cells

**Fig. 3:**
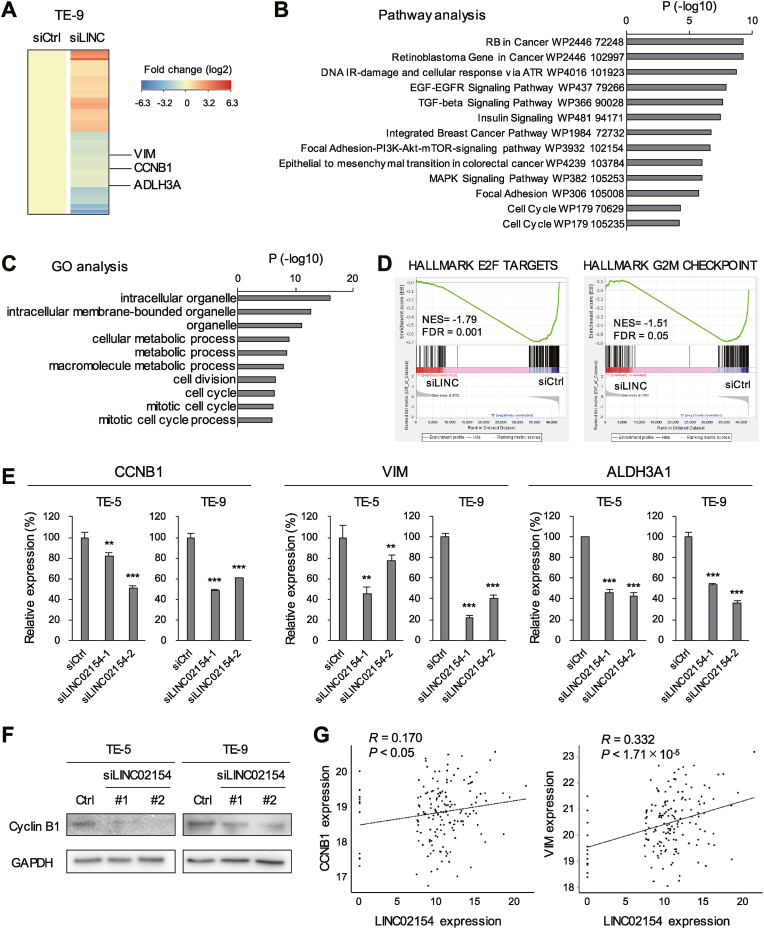
The effects of LINC02154 knockdown on gene expression profiles in ESCA cells. (A) Results of RNA-seq in TE-9 cells transfected with a control siRNA (siCtrl) or a siRNA targeting LINC02154 (siLINC). Shown is a heatmap of genes altered (>2-fold) by LINC02154 knockdown. (B, C) Pathway (B) and gene ontology (GO) analyses (C) of genes downregulated by LINC02154 knockdown. (D) Results of GSEA for indicated gene sets based on the RNA-seq data in (A). (E) qRT-PCR analysis of indicated genes in ESCA cells transfected with the indicated siRNAs. (n = 3). (F) Western blot analysis of cyclin B1 in ESCA cells transfected with the indicated siRNAs. (G) Correlations between expression levels of LINC02154 and those of CCNB1 or VIM in TCGA-ESCA dataset. Error bars represent SDs. ∗∗*P* < 0.01, ∗∗∗*P* < 0.001.

To further clarify the mechanism by which LINC02154 exerts its oncogenic effects, we performed RNA-seq analysis to assess the effect of LINC02154 knockdown on gene expression in ESCA cells (TE-9). We found that following LINC02154 knockdown, 2974 genes were upregulated (>2-fold) while 2991 genes were downregulated (>2-fold) (Fig. 3A, Supplementary Table 4). Pathway analysis revealed that genes associated with various cancer signaling pathways, including EMT and cell cycle processes, were significantly enriched among genes downregulated by LINC02154 knockdown (Fig. 3B). GO analysis suggested that genes associated with organelle, metabolic process and cell cycle were significantly enriched among the downregulated genes following LINC02154 knockdown (Fig. 3C). GSEA also demonstrated that genes involved in the cell cycle (E2F targets and the G2M checkpoint) were significantly enriched among genes downregulated by LINC02154 knockdown (Fig. 3D). Subsequent qRT-PCR analysis confirmed that expression of cyclin B1 (CCNB1), vimentin (VIM), and aldehyde dehydrogenase 3 family member A1 (ALDH3A1) was suppressed in ESCA cells following LINC02154 knockdown (Fig. 3A and E), and western blotting further demonstrated the downregulation of cyclin B1 in ESCA cells after LINC02154 knockdown (Fig. 3F). Moreover, analysis of TCGA-ESCA data revealed a positive correlation between levels of LINC02154 expression and those of CCNB1 and VIM expression in primary tumors (Fig. 3G).

### 3.4 LINC02154 regulates EMT and chemoresistance by interacting with miR-200b

**Fig. 4:**
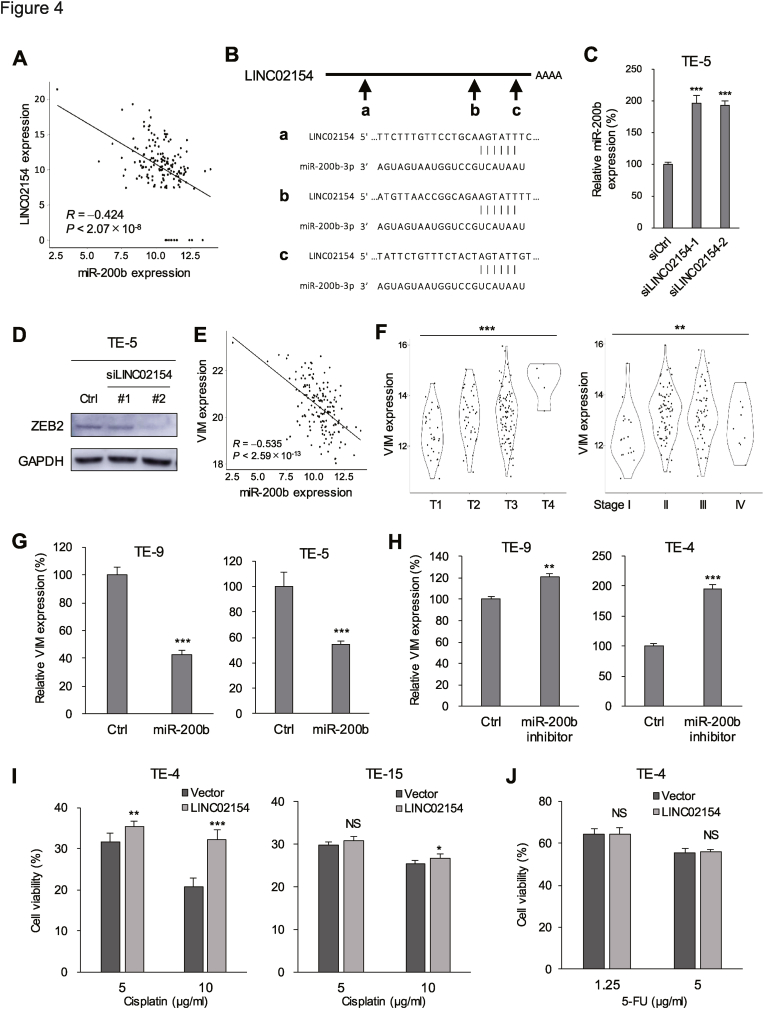
LINC02154 promotes EMT through suppression of miR-200b. (A) Correlation between levels of miR-200b expression and those of LINC02154 expression in TCGA-ESCA dataset. (B) Putative miR-200b-3p binding sites in the LINC02154 sequence. (C) qRT-PCR analysis of miR-200b in TE-5 cells transfected with a control siRNA or siRNAs targeting LINC02154. (n = 3). (D) Western blot analysis of ZEB2 in TE-5 cells transfected with the indicated siRNAs. (E) Correlation between levels of miR-200b expression and those of VIM in TCGA-ESCA dataset. (F) Correlation between VIM expression and T-factors (left) and clinical stages (right) in TCGA-ESCA dataset. (G) qRT-PCR analysis of VIM in ESCA cells transfected with a miR-mimic control or a miR-200b mimic. (n = 3). (H) qRT-PCR analysis of VIM in ESCA cells transfected with a miR-inhibitor control or a miR-200b inhibitor. (n = 3). (I, J) Results of cell viability assays in ESCA cells infected with the indicated vectors. Cells were treated with the indicated concentrations of cisplatin (H) or 5-FU (I). (n = 6). Error bars represent SDs. ∗∗*P* < 0.01, ∗∗∗*P* < 0.001, NS, not significant.

It is well documented that many lncRNAs exert their effects through interaction with microRNAs (miRNAs). We therefore used miRcode to search for miRNAs that may associate with LINC02154 and found 15 potential miRNA binding sites within LINC02154 (Supplementary Table 5). Among the miRNAs potentially associating with LINC02154, several exhibited a significant inverse correlation with LINC02154 expression levels (Supplementary Table 6). Among those, we noted that the levels of several miR-200 family members (miR-200b, miR-200c, and miR-429) exhibited tight negative correlations with those of LINC02154 in TCGA-ESCA dataset (Fig. 4A, Supplementary Fig. 2A). Moreover, using scanMiR, we identified three potential miR-200b–3b binding sites within the LINC02154 sequence (Fig. 4B).

LINC02154 knockdown significantly upregulated miR-200b-3p expression in TE-5 cells, suggesting LINC02154 negatively regulates miR-200b-3p in ESCA cells (Fig. 4C). It is well established that miR-200b inhibits EMT by suppressing expression of ZEB1 and ZEB2 [21]. Because we detected little ZEB1 expression in ESCA cell lines (data not shown), we assessed the effect of LINC02154 knockdown on ZEB2 and found that ZEB2 expression was diminished in ESCA cells transfected with siRNA targeting LINC02154 (Fig. 4D). In addition, as shown in Fig. 3G, levels of LINC02154 expression correlated positively with those of the EMT marker VIM. Consistent with those observations, levels of VIM expression correlated inversely with those of miR-200b in primary ESCA tumors (Fig. 4E). Upregulation of VIM was also associated with larger tumor size and more advanced clinical stage in primary tumors (Fig. 4F). Moreover, ectopic expression of miR-200b suppressed VIM expression in ESCA cells, while inhibition of miR-200b had the opposite effect (Fig. 4G and H). Because miR-200b reportedly attenuates chemoresistance in various types of cancer [22], we next investigated the effects of LINC02154 on chemoresistance in ESCA cells. Ectopic expression of LINC02154 enhanced resistance to cisplatin but did not affect sensitivity to 5-FU (Fig. 4I and J).

### 3.5 Identification of proteins that interact with LINC02154 in ESCA cells

**Fig. 5:**
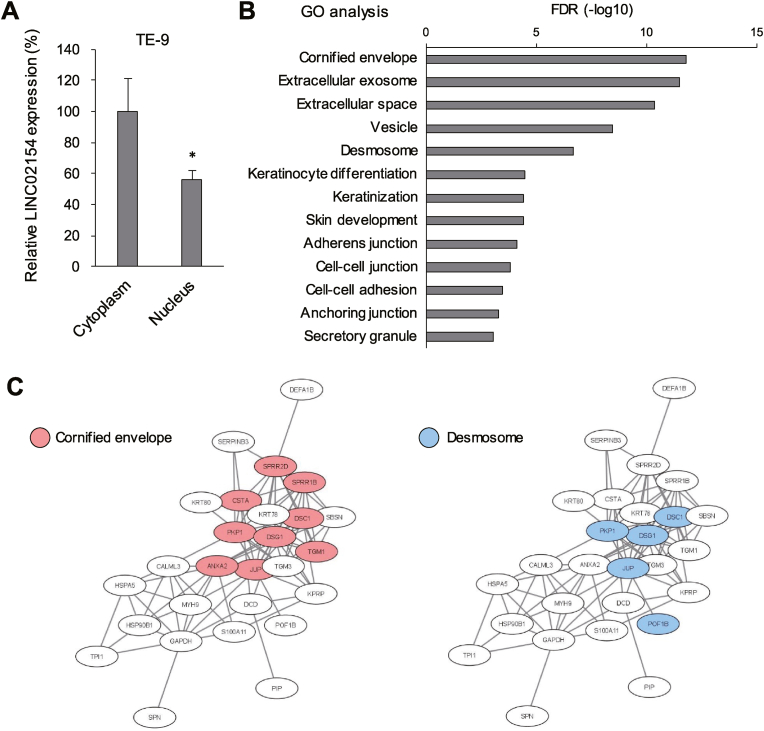
Identification of proteins that interact with LINC02154 in ESCA cells. (A) qRT-PCR analysis of LINC02154 in the cytoplasmic and nuclear fractions extracted from TE-9 cells. (n = 3). (B) Gene ontology (GO) analysis of proteins identified by mass spectrometry that potentially interact with LINC02154. (C) Interaction network among the proteins that potentially interact with LINC02154. Error bars represent SDs. ∗*P* < 0.05.

To determine the intracellular localization of LINC02154 in ESCA cells, we extracted RNAs from the cytoplasmic and nuclear fractions of TE-9 cells. qRT-PCR analysis of GAPDH and U6 confirmed the successful separation of respective fractions (Supplementary Fig. 4A). Subsequent qRT-PCR analysis revealed that LINC02154 is predominantly localized in the cytoplasm (Fig. 5A). To identify cytoplasmic proteins that interact with LINC02154 in ESCA cells, we performed RNA-pulldown followed by mass spectrometry analysis using cytoplasmic extracts from TE-5 cells and identified 32 proteins that potentially associate with LINC02154 (Supplementary Table 7). GO and protein-protein interaction analyses revealed that proteins associating with the cornified envelope and desmosome were enriched among the identified proteins (Fig. 5B and C).

## 4 Discussion

In the present study, we demonstrated that LINC02154 is upregulated and associated with activation of several oncogenic pathways in ESCA. We also confirmed that LINC02154 knockdown suppresses ESCA cell proliferation and migration, while its overexpression promotes colony formation. Moreover, we showed that LINC02154 may promote EMT by suppressing miR-200b expression. These findings are consistent with recent studies reporting the potential involvement of LINC02154 in various human malignancies. For instance, elevated LINC02154 expression reportedly correlates with poorer overall survival in patients with laryngeal squamous cell carcinoma (LSCC) [20], and LINC02154 was identified as a negative prognostic factor for LSCC patients [23]. LINC02154 also promotes proliferation and metastasis of hepatocellular carcinoma by activating the SPC24 and PI3K-AKT signaling pathways [19]. In addition, another recent study highlighted LINC02154 as a cuproptosis-related lncRNA in clear cell renal cell carcinoma [24]. We recently showed that upregulation of LINC02154 promotes cell cycling and enhances mitochondrial function in oral squamous cell carcinoma (OSCC) [13]. Consistent with that earlier study, here we found that LINC02154 knockdown suppresses expression of a number of cell cycle-related genes, including CCNB1.

Our RNA-seq data also demonstrated that LINC02154 knockdown reduces expression of metabolism-associated genes, including ALDH3A1. In non-small cell lung carcinoma, hypoxia-induced ALDH3A1 expression suppresses oxidative phosphorylation and promotes cell proliferation [25]. ALDH3A1 is also upregulated in pancreatic cancer tissues, where it plays a crucial role in tumor progression and immune suppression [26]. In ESCA, ALDH3A1 is regulated by the core regulatory circuitry transcription factors TP63, SOX2 and KLF5 and is involved in the proliferation of cancer cells [27]. Our results suggest that LINC02154 promotes ESCA cell proliferation, at least in part, through upregulation of ALDH3A1.

By searching for miRNAs that potentially interact with LINC02154, we found that LINC02154 negatively regulates miR-200b expression. It is well known that the miR-200 family regulates the expression of the ZEB family and suppresses EMT, and that ZEB2 enhances the promoter activity of VIM, suggesting that LINC02154 activates VIM through the LINC02154/miR-200b/ZEB2 axis [28,29]. In ESCC, miR-200b suppresses cell growth and induces cell cycle arrest by modulating expression of cell cycle regulator genes and the Wnt/β catenin signaling pathway [30], and the miR-200 family post-transcriptionally suppresses ZEB1 and ZEB2, which are crucial EMT activators in various types of cancers [31,32]. We observed that LINC02154 knockdown attenuates ZEB2 protein expression in ESCA cells, likely due to the upregulation of miR-200b. We also observed that LINC02154 knockdown reduces expression of the EMT marker VIM in ESCA cells. VIM encodes an intermediate filament protein, and its expression was shown to be a prognostic marker in ESCC [33]. Moreover, VIM expression is associated with larger tumor size, lymph node metastasis, and distant metastasis in ESCC [34], highlighting the significance of VIM expression in the progression of ESCA. Taken together, these results suggest that LINC02154 promotes EMT and upregulates VIM by suppressing miR-200b.

Our RNA-pulldown and mass spectrometry analysis revealed potential interactions between LINC02154 and proteins associated with the cornified envelope or desmosome. The cornified envelope is involved in cornified layer formation, which marks the endpoint of normal epidermal differentiation and cell death [35]. In the normal epidermis, the cornified envelope is composed of structural proteins including loricrin, involucrin and small proline-rich proteins (SPRRs) at the skin surface [36]. In head and neck squamous cell carcinoma, a cornification marker, SPRR3, is associated with cancer cell differentiation [37]. SPRR3 expression is also reportedly downregulated in esophageal epithelial dysplasia [38]. Additionally, expression of involucrin correlates with differentiation in ESCA cells [39]. These observations suggest these proteins and their interaction with LINC02154 may play an essential role in the development of squamous cell carcinoma.

Desmosomes are the most conspicuous adhesive structures in epithelial cells and are connected to the intermediate filament cytoskeletal network [40]. They are composed of proteins that belong to the desmosomal cadherin family, the armadillo family and the plakin family of cytolinkers [41]. In cancer, altered expression of desmosomal cadherins leads to the release of plakoglobin from desmosomes and activation of the Wnt/β catenin signaling pathway [41]. The induction of EMT leads to downregulation of desmosomal proteins, including desmoplakin and plakophilin-2 [42]. Moreover, plakoglobin overexpression reportedly promotes cisplatin resistance in epidermoid carcinoma [43]. Given the critical roles of desmosomes and their components in cancer progression, our results suggest that LINC02154 may promote ESCA progression and drug resistance by interacting with desmosomal proteins.

There are several limitations in this study. For instance, depletion of LINC02154 affects a number of genes involved in cancer pathways in ESCA cells, but the mechanism how LINC02154 regulate those genes is not fully understood. In our previous study, we found that LINC02154 regulates cell cycle-related genes through modulating a master regulator FOXM1 in OSCC cells, suggesting that similar mechanism is involved in ESCA cells. However, it remains unclear how LINC02154 regulates metabolic genes including ALDH3A1 in ESCA cells. Similarly, the mechanism how LINC02154 regulates miR-200b is not fully elucidated. Our finding of the multiple potential miR-200b-3p binding sites within the LINC02154 sequence suggests that LINC02154 may act as competing endogenous RNA against miR-200b-3p, while direct interaction between LINC02154 and miR-200b-3p needs to be validated. Finally, although we identified a series of proteins which potentially interact with LINC02154, their involvement in the oncogenic function of LINC02154 remains to be elucidated. Further studies are warranted to fully uncover the molecular functions of LINC02154 in ESCA cells.

## 5 Conclusion

In conclusion, our results demonstrate that LINC02154 is upregulated in primary ESCA and likely contributes to disease progression. LINC02154 promotes cell cycling, EMT, and chemoresistance, at least in part, through downregulation of miR-200b. Moreover, LINC02154 plays a role in ESCA progression by interacting with multiple intracellular proteins involved in various oncogenic pathways. These findings suggest that LINC02154 may represent a novel therapeutic target for ESCA treatment.

## CRediT authorship contribution statement

**Kotoha Shimote:** Writing – original draft, Visualization, Investigation. **Takeshi Niinuma:** Writing – original draft, Visualization, Investigation, Funding acquisition, Formal analysis, Data curation. **Hiroshi Kitajima:** Writing – review & editing, Methodology, Funding acquisition. **Kazuya Ishiguro:** Writing – review & editing, Validation. **Eiichiro Yamamoto:** Writing – review & editing. **Gota Sudo:** Writing – review & editing, Validation. **Akira Yorozu:** Writing – review & editing, Validation. **Mutsumi Toyota:** Investigation. **Masahiro Kai:** Writing – review & editing, Funding acquisition. **Masashi Idogawa:** Writing – review & editing. **Hiromu Suzuki:** Writing – original draft, Supervision, Project administration, Funding acquisition, Data curation, Conceptualization.

## Ethics approval and consent to participate

Not applicable.

## Data availability statement

RNA-seq data were deposited in NCBI SRA (accession ID, PRJNA1219221).

## Funding statement

This study was supported in part by JSPS KAKENHI Grant Numbers 21K07945 (T. Niinuma), 24K11173 (T. Niinuma), 24K10335 (H. Kitajima), 22K05444 (M. Kai), and 22H02925 (H. Suzuki), and the Takeda Science Foundation (T. Niinuma, 2018).

## Declaration of competing interest

The authors declare that they have no known competing financial interests or personal relationships that could have appeared to influence the work reported in this paper.
