# Supplementary material for: Upregulation of LINC02154 promotes esophageal cancer progression by enhancing cell cycling and epithelial-mesenchymal transition
Source: Noncoding RNA Res. 2025 Jun 2;14:107–16. doi: 10.1016/j.ncrna.2025.06.001 (PMC12173678; doi:10.1016/j.ncrna.2025.06.001)
Supplement: Multimedia component 1 [file mmc1.docx]

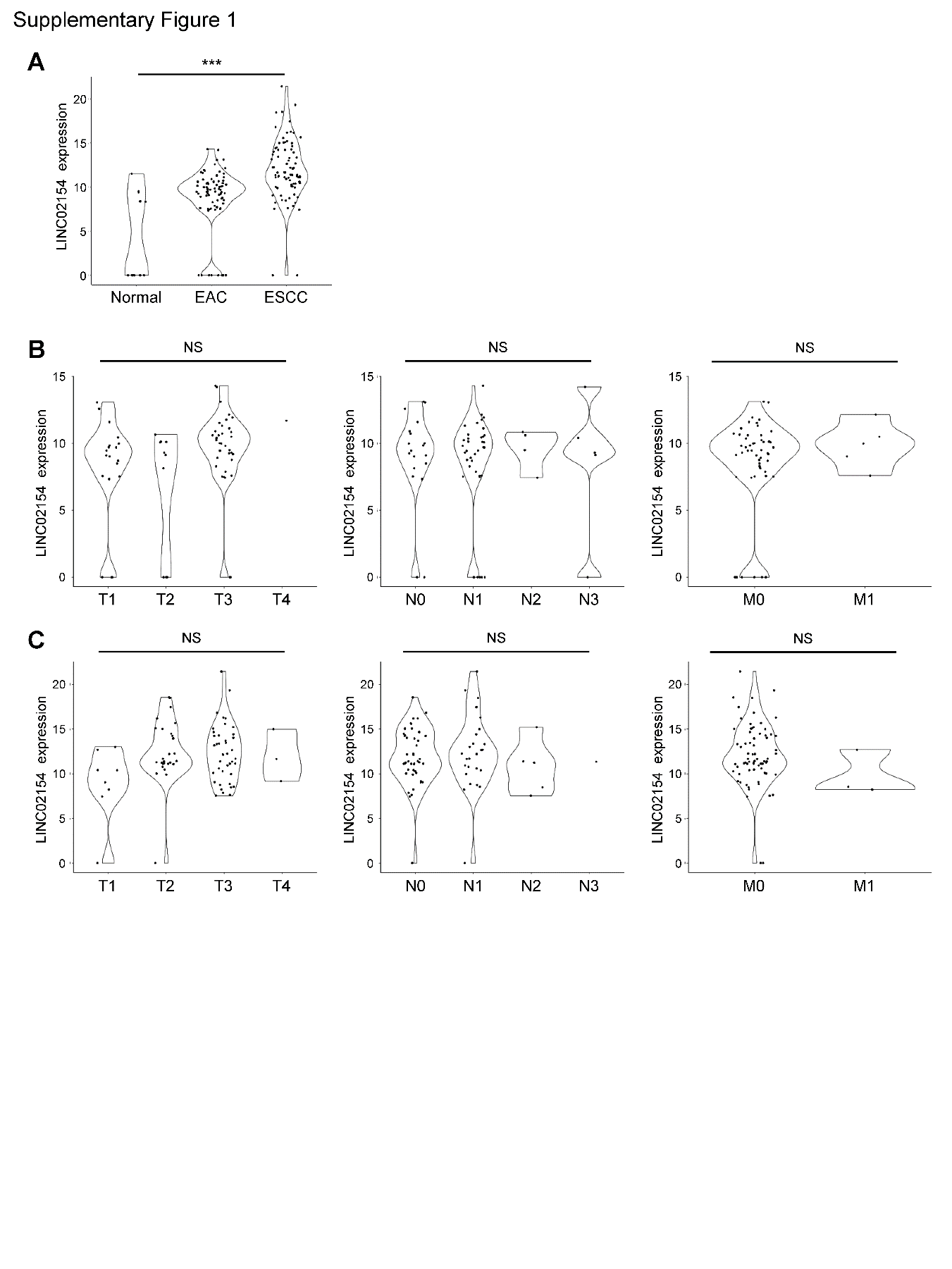


**Supplementary Figure 1**

Expression of LINC02154 and its correlation with clinical factors in primary ESCA tumors. (A) Levels of LINC02154 expression in normal esophageal tissues (n = 11), EAC tumors (n = 79) and ESCC tumors (n = 81) in TCGA-ESCA dataset. (B, C) Correlations between levels of LINC02154 expression and T-factors (left), N-factors (middle) or M-factors (right) in EAC tumors (B) and ESCC tumors (C) in TCGA-ESCA dataset. ****P*<0.001, NS: not significant.


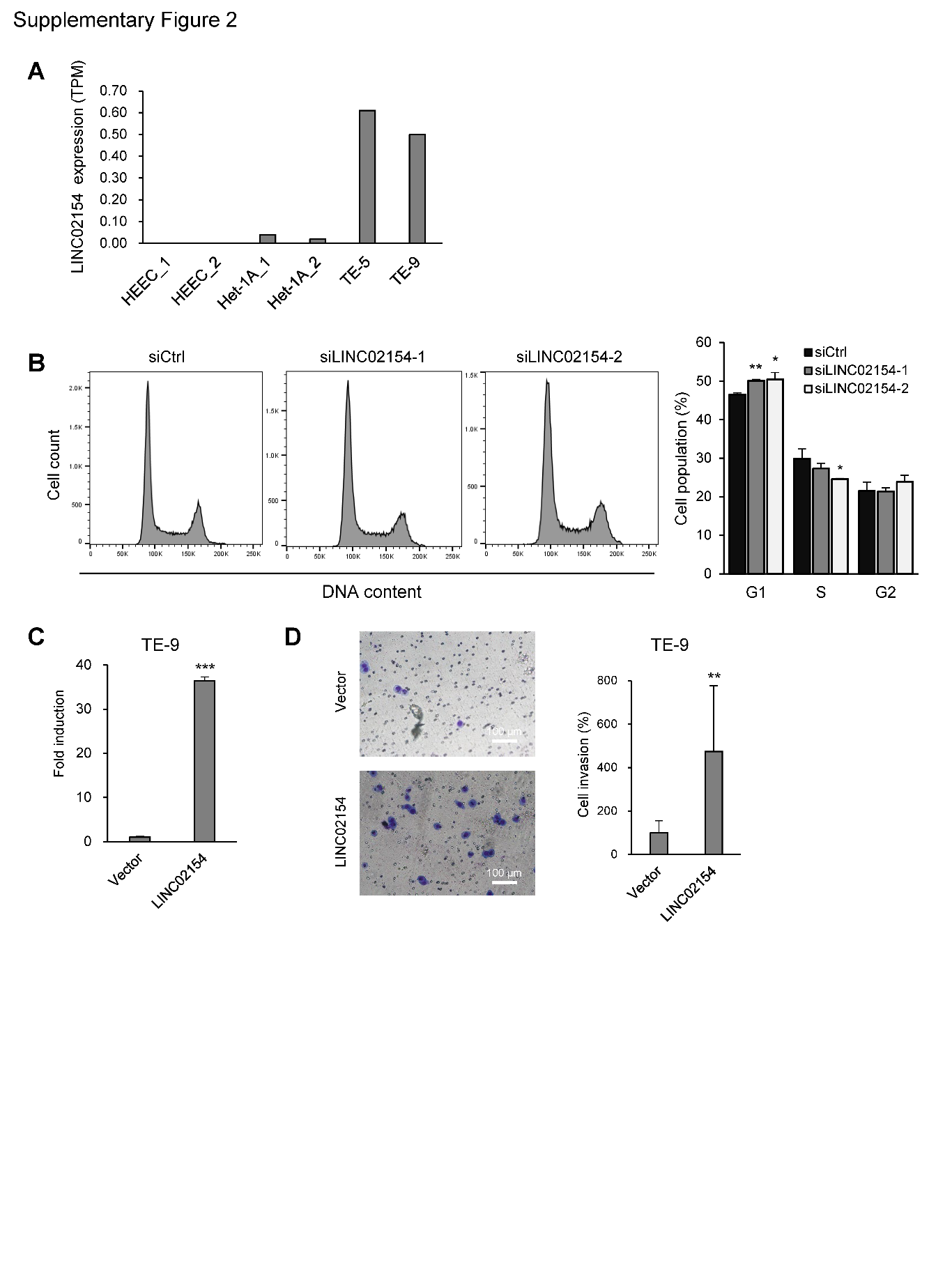


**Supplementary Figure 2**

Functional analysis of LINC02154 in ESCA cells. (A) Expression levels of LINC02154 in human esophageal epithelial cells (HEEC), Het-1A, and ESCA cell lines (TE-5, TE-9). (B) Cell cycle analysis in TE-5 cells transfected with a control siRNA or siRNAs targeting LINC02154. Representative results are shown on the left, summarized results on the right. (n = 3). (C) qRT-PCR analysis of LINC02154 expression in TE-9 cells infected with a control vector or a LINC02154 expression vector. (n = 3). (D) Cell invasion assays with TE-9 cells infected with the indicated vector. Summarized results are shown on the right. (n = 3). **P*<0.05, ***P*<0.01, ****P*<0.001.


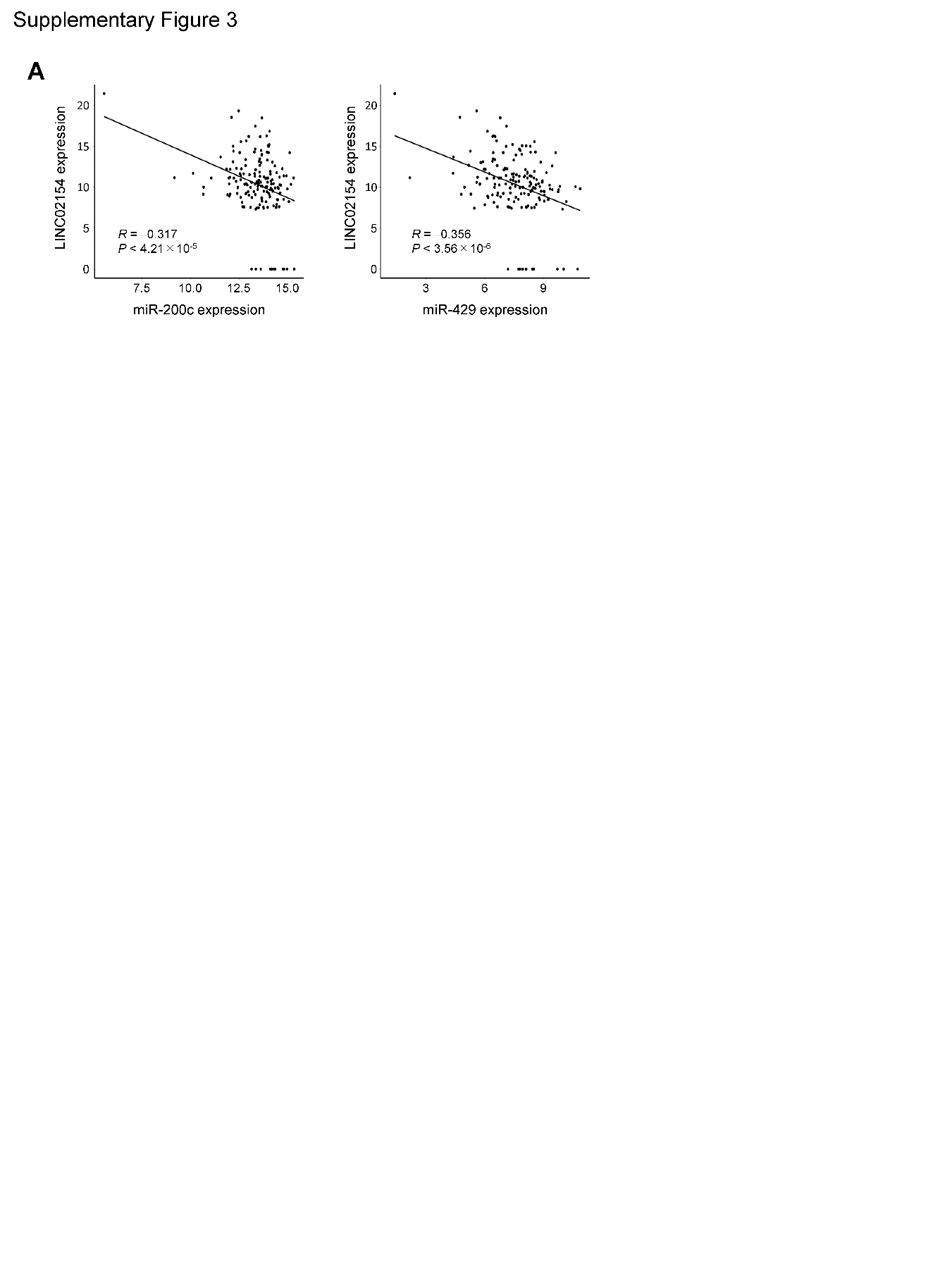


**Supplementary Figure 3**

Correlations between levels of LINC02154 expression and those of miR-200c or miR-429 in TCGA‑ESCA dataset.


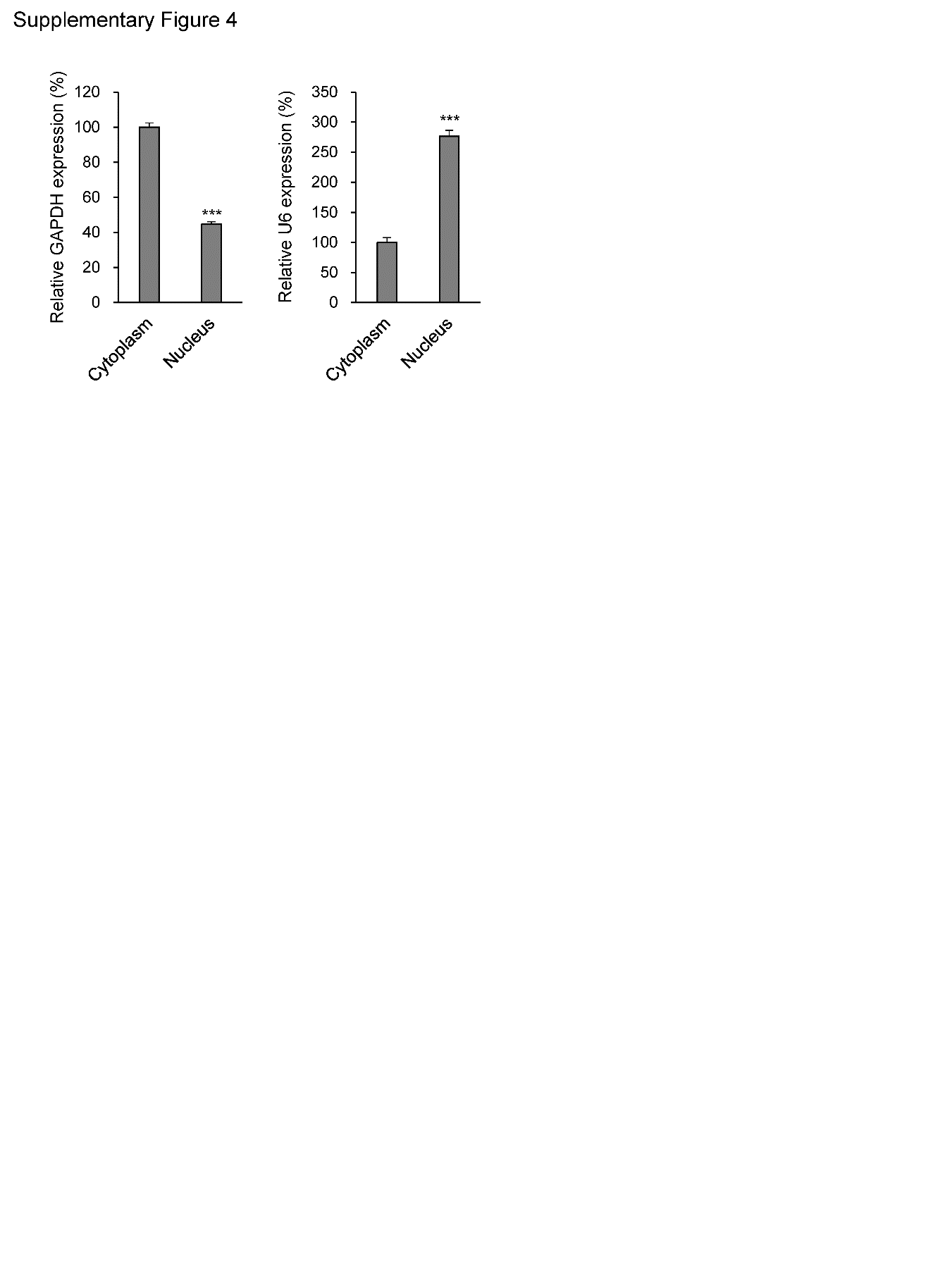


**Supplementary Figure 4**

qRT-PCR analysis of GAPDH (left) and U6 (right) in the cytoplasmic and nuclear fractions extracted from TE-9 cells. (n = 3). ****P*<0.001.
