# Supplementary material for: Upregulation of LINC02154 promotes esophageal cancer progression by enhancing cell cycling and epithelial-mesenchymal transition
Source: Noncoding RNA Res. 2025 Jun 2;14:107–16. doi: 10.1016/j.ncrna.2025.06.001 (PMC12173678; doi:10.1016/j.ncrna.2025.06.001)
Supplement: Multimedia component 2 [file mmc2.pdf]

Supplementary Table 1. Sequences of siRNAs and shRNAs used in this study

Name	Sequence
si-LINC02154-1	5'-GAAUUACUUAAUGAUCAUUGUCUTT-3'
si-LINC02154-2	5'-ACUCAGUCUCUAUGAUGGUAUUAGT-3'

Supplementary Table 2. Sequences of the primers used in this study

qRT-PCR		
LINC02154	Forward	5'-TGCCGTGATTGTGAGGCCTC-3'
	Reverse	5'-GCTGCCAGTTACTAATACCATCATAG-3'
B2M	Forward	5'-ACTCTCTCTTTCTGGCCTGGA-3'
	Reverse	5'-TCTCTGCTGGATGACGTGAG-3'
CCNB1	Forward	5'-TTCCAGTTATGCAGCACCTG-3'
	Reverse	5'-AGTGCAGAATTCAGCTGTGG-3'
ALDH3A1	Forward	5'-GATCCAGGAGCAGGAGCAG-3'
	Reverse	5'-AGGGAGCTTCTGGATCATGTAC-3'
VIM	Forward	5'-TGACCTTGAACGCAAAGTGG-3'
	Reverse	5'-TCAGGCTTGGAAACATCCAC-3'
RT-PCR		
Full length LINC02154	Forward	5'-TTAGTGAACCGGATCCCCATGTAAGAAGTCCCTTTG-3'
	Reverse	5'-AGCCTCCCCCAAGCTTGGCAGTTCAGGTTTTAATGG-3'

Supplementary Table 3. LncRNAs upregulated in ESCA

	TCGA ESCA		GSE130078	
LncRNA genes	Fold change	P	Fold change	P
ENSG00000236347.1	492.84	3.76E-11	86.00	5.12E-09
ENSG00000266869.1	410.38	3.02E-08	28.33	5.41E-09
ENSG00000235277.1	50.91	3.24E-06	20.50	3.53E-08
ENSG00000260976.1	287.29	1.83E-12	69.50	6.53E-07
ENSG00000248268.1	55.96	2.47E-07	94.00	1.96E-06
ENSG00000250509.1	237.37	7.18E-08	67.00	2.54E-05
ENSG00000203446.2	78.83	7.34E-13	62.00	2.67E-05
ENSG00000251151.2	32.62	5.89E-06	72.00	3.53E-05
ENSG00000250133.2	60.88	9.60E-19	17.60	4.28E-05
ENSG00000223485.1	30.20	1.67E-07	127.00	1.12E-04
ENSG00000271216.1	357.15	8.71E-09	271.00	7.36E-04
ENSG00000266401.1	43.37	2.03E-06	62.60	1.47E-03
ENSG00000230838.1	21.83	7.59E-06	72.00	6.37E-03
ENSG00000230533.1	63.08	1.17E-06	19.80	6.96E-03
ENSG00000235385.1	67.28	2.50E-06	61.75	8.36E-03
ENSG00000233589.1	35.04	3.51E-10	33.50	1.36E-01

Supplementary Table 4. Genes whose expression was altered by LINC02154 knockdown in TE-9 cells

Gene	Fold change	Gene Symbol
ENSG00000235174	-530.23	
ENSG00000276525	-208.66	
ENSG00000256512	-134.92	
ENSG00000278603	-107.91	
ENSG00000227726	-83.34	
ENSG00000261779	-83.34	
ENSG00000237566	-76.01	
ENSG00000288623	-71.13	
ENSG00000277922	-58.88	
ENSG00000284636	-58.88	
ENSG00000284687	-57.28	
ENSG00000231471	-56.89	
ENSG00000233838	-55.51	
ENSG00000262636	-44.59	
ENSG00000257767	-42.58	
ENSG00000170100	-40.45	ZNF778
ENSG00000234319	-37.93	
ENSG00000275895	-37.47	U2AF1L5
ENSG00000267458	-37.28	
ENSG00000240211	-34.76	
ENSG00000254866	-33.81	
ENSG00000240731	-33.79	
ENSG00000202058	-30.32	
ENSG00000240373	-30.09	
ENSG00000259112	-29.69	NDUFC2-KCTD14
ENSG00000255115	-29.02	
ENSG00000271424	-28.47	
ENSG00000185847	-28.14	LINC01405
ENSG00000232059	-27.81	
ENSG00000264501	-26.13	
ENSG00000267379	-26.08	
ENSG00000267059	-25.17	
ENSG00000253744	-24.62	
ENSG00000250012	-24.52	
ENSG00000167774	-24.50	
ENSG00000276757	-24.35	
ENSG00000230834	-24.17	
ENSG00000255329	-23.88	
ENSG00000134049	-23.77	

ENSG00000240993	-23.46	
ENSG00000170946	-22.99	DNAJC24
ENSG00000260342	-22.79	
ENSG00000238288	-22.38	
ENSG00000242861	-22.19	
ENSG00000139174	-22.12	PRICKLE1
ENSG00000204335	-22.00	SP5
ENSG00000232527	-21.24	LOC100996732
ENSG00000237343	-20.41	
ENSG00000228606	-20.40	
ENSG00000248256	-20.34	
ENSG00000272402	-20.11	
ENSG00000235369	-19.98	
ENSG00000259258	-19.63	
ENSG00000258325	-19.45	
ENSG00000241992	-19.35	
ENSG00000225721	-19.26	
ENSG00000178458	-19.19	
ENSG00000215472	-19.14	RPL17-C18orf32
ENSG00000105650	-18.54	LOC729966
ENSG00000258102	-18.42	MAP1LC3B2
ENSG00000280346	-18.40	
ENSG00000132016	-18.24	C19orf57
ENSG00000273619	-18.19	
ENSG00000103047	-17.41	TANGO6
ENSG00000263990	-17.11	
ENSG00000271889	-17.02	
ENSG00000234806	-16.95	
ENSG00000286803	-16.87	
ENSG00000243738	-16.62	
ENSG00000224126	-16.50	
ENSG00000271236	-16.36	
ENSG00000171100	-16.22	MTM1
ENSG00000226853	-16.18	
ENSG00000102243	-16.15	VGLL1
ENSG00000265393	-16.11	
ENSG00000227632	-15.96	
ENSG00000279807	-15.89	
ENSG00000131097	-15.78	HIGD1B
ENSG00000284954	-15.72	
ENSG00000267255	-15.65	
ENSG00000231513	-15.55	

ENSG00000272009	-15.53	LOC102724889
ENSG00000034533	-15.48	ASTE1
ENSG00000130775	-15.44	THEMIS2
ENSG00000180178	-15.42	FAR2P1
ENSG00000111196	-15.41	MAGOHB
ENSG00000225080	-15.34	
ENSG00000272128	-15.28	
ENSG00000270591	-15.17	
ENSG00000258800	-15.16	
ENSG00000267301	-15.15	
ENSG00000231503	-15.04	
ENSG00000186314	-14.90	PRELID2
ENSG00000107295	-14.83	SH3GL2
ENSG00000140807	-14.81	NKD1
ENSG00000242307	-14.80	
ENSG00000247572	-14.79	CKMT2-AS1
ENSG00000255229	-14.78	
ENSG00000137720	-14.67	C11orf1
ENSG00000257084	-14.56	MIR200CHG
ENSG00000243970	-14.54	
ENSG00000213519	-14.51	
ENSG00000172375	-14.36	C2CD2L
ENSG00000265840	-14.30	
ENSG00000267135	-14.30	
ENSG00000260482	-14.27	
ENSG00000263847	-14.08	
ENSG00000159788	-14.07	RGS12
ENSG00000180008	-14.03	SOCS4
ENSG00000119969	-13.95	HELLS
ENSG00000235169	-13.94	SMIM1
ENSG00000178105	-13.94	DDX10
ENSG00000182585	-13.92	EPGN
ENSG00000177707	-13.84	NECTIN3
ENSG00000259066	-13.77	
ENSG00000232573	-13.69	
ENSG00000196358	-13.62	NTNG2
ENSG00000220920	-13.57	
ENSG00000267710	-13.54	
ENSG00000264769	-13.28	
ENSG00000162944	-13.27	RFTN2
ENSG00000241568	-13.17	
ENSG00000273965	-13.17	

ENSG00000246575	-13.08	
ENSG00000115946	-13.07	PNO1
ENSG00000224114	-13.02	
ENSG00000261326	-13.01	
ENSG00000232901	-12.99	
ENSG00000253366	-12.95	
ENSG00000242715	-12.94	CCDC169
ENSG00000064933	-12.91	PMS1
ENSG00000229119	-12.91	
ENSG00000036054	-12.88	TBC1D23
ENSG00000201684	-12.82	
ENSG00000263815	-12.82	
ENSG00000143190	-12.77	POU2F1
ENSG00000229988	-12.65	
ENSG00000255164	-12.65	
ENSG00000276096	-12.65	
ENSG00000042980	-12.62	ADAM28
ENSG00000203397	-12.62	
ENSG00000243660	-12.53	
ENSG00000261731	-12.53	
ENSG00000272655	-12.53	
ENSG00000239494	-12.49	
ENSG00000280502	-12.49	
ENSG00000238251	-12.44	
ENSG00000125730	-12.41	C3
ENSG00000235859	-12.37	
ENSG00000284776	-12.36	
ENSG00000243420	-12.33	
ENSG00000265656	-12.19	
ENSG00000225039	-12.14	LINC01058
ENSG00000279352	-12.10	
ENSG00000151532	-12.04	VTI1A
ENSG00000165490	-12.04	DDIAS
ENSG00000267681	-12.02	
ENSG00000164331	-11.93	ANKRA2
ENSG00000250575	-11.90	
ENSG00000230929	-11.88	
ENSG00000114771	-11.86	AADAC
ENSG00000258732	-11.86	
ENSG00000241769	-11.81	LINC00893
ENSG00000110693	-11.74	SOX6
ENSG00000261240	-11.71	

ENSG00000267735	-11.71	
ENSG00000271218	-11.71	
ENSG00000281501	-11.71	
ENSG00000271746	-11.68	
ENSG00000284969	-11.64	
ENSG00000261848	-11.63	
ENSG00000113812	-11.62	ACTR8
ENSG00000255010	-11.55	
ENSG00000285668	-11.55	
ENSG00000259409	-11.46	
ENSG00000182568	-11.40	SATB1
ENSG00000258666	-11.33	
ENSG00000259710	-11.33	
ENSG00000179111	-11.31	HES7
ENSG00000232363	-11.22	
ENSG00000242136	-11.15	
ENSG00000251131	-11.05	
ENSG00000205090	-10.96	TMEM240
ENSG00000258311	-10.95	
ENSG00000278558	-10.91	TMEM191B
ENSG00000203321	-10.87	
ENSG00000287580	-10.85	
ENSG00000143401	-10.84	ANP32E
ENSG00000226429	-10.80	
ENSG00000128534	-10.77	LSM8
ENSG00000225419	-10.68	
ENSG00000285128	-10.67	
ENSG00000213275	-10.59	
ENSG00000225193	-10.59	
ENSG00000247137	-10.54	
ENSG00000286095	-10.50	
ENSG00000145819	-10.48	ARHGAP26
ENSG00000224680	-10.46	
ENSG00000226281	-10.46	
ENSG00000156097	-10.44	GPR61
ENSG00000274124	-10.43	
ENSG00000012779	-10.41	ALOX5
ENSG00000259345	-10.28	
ENSG00000236878	-10.24	
ENSG00000114861	-10.21	FOXP1
ENSG00000090924	-10.19	PLEKHG2
ENSG00000228115	-10.17	

ENSG00000255933	-10.15	
ENSG00000075035	-10.11	WSCD2
ENSG00000165269	-10.11	AQP7
ENSG00000080298	-10.06	RFX3
ENSG00000260277	-10.06	
ENSG00000158406	-9.99	HIST1H4H
ENSG00000145287	-9.97	PLAC8
ENSG00000164109	-9.94	MAD2L1
ENSG00000214654	-9.92	
ENSG00000231871	-9.91	IPO9-AS1
ENSG00000137821	-9.89	LRRC49
ENSG00000197170	-9.88	PSMD12
ENSG00000183479	-9.83	TREX2
ENSG00000244405	-9.83	ETV5
ENSG00000272170	-9.83	
ENSG00000125144	-9.78	MT1G
ENSG00000186642	-9.78	PDE2A
ENSG00000268643	-9.78	
ENSG00000268434	-9.76	
ENSG00000262920	-9.73	
ENSG00000183785	-9.71	TUBA8
ENSG00000114854	-9.69	TNNC1
ENSG00000230510	-9.60	PPP5D1
ENSG00000213177	-9.56	
ENSG00000132300	-9.53	PTCD3
ENSG00000235888	-9.50	
ENSG00000157890	-9.48	MEGF11
ENSG00000143702	-9.46	CEP170
ENSG00000156795	-9.45	WDYHV1
ENSG00000257390	-9.45	
ENSG00000132330	-9.45	SCLY
ENSG00000232075	-9.43	
ENSG00000243433	-9.43	
ENSG00000261008	-9.43	LINC01572
ENSG00000232739	-9.42	
ENSG00000131374	-9.37	TBC1D5
ENSG00000175567	-9.36	UCP2
ENSG00000244361	-9.29	
ENSG00000229018	-9.28	
ENSG00000111725	-9.28	PRKAB1
ENSG00000260269	-9.27	
ENSG00000196227	-9.26	FAM217B

ENSG00000228812	-9.26	LAMA5-AS1
ENSG00000011198	-9.17	ABHD5
ENSG00000121895	-9.14	TMEM156
ENSG00000258967	-9.11	
ENSG00000260018	-9.11	
ENSG00000117010	-9.10	ZNF684
ENSG00000196459	-9.07	TRAPPC2
ENSG00000254854	-9.05	LOC102724301
ENSG00000265490	-9.01	
ENSG00000134146	-8.96	DPH6
ENSG00000279226	-8.92	
ENSG00000279121	-8.90	
ENSG00000143556	-8.86	S100A7
ENSG00000228802	-8.86	
ENSG00000082146	-8.84	STRADB
ENSG00000254685	-8.84	FPGT
ENSG00000100744	-8.80	GSKIP
ENSG00000237310	-8.78	GS1-124K5.4
ENSG00000275017	-8.76	
ENSG00000180917	-8.72	CMTR2
ENSG00000106541	-8.72	AGR2
ENSG00000250734	-8.69	
ENSG00000258745	-8.69	
ENSG00000265334	-8.68	
ENSG00000269374	-8.66	
ENSG00000285708	-8.65	
ENSG00000248996	-8.64	
ENSG00000250027	-8.64	
ENSG00000273958	-8.64	
ENSG00000108064	-8.64	TFAM
ENSG00000100298	-8.59	APOBEC3H
ENSG00000271259	-8.55	
ENSG00000185615	-8.53	PDIA2
ENSG00000262959	-8.53	
ENSG00000175352	-8.52	NRIP3
ENSG00000235605	-8.48	
ENSG00000260778	-8.47	LOC106660606
ENSG00000232788	-8.45	
ENSG00000279469	-8.45	
ENSG00000179673	-8.45	RPRML
ENSG00000251556	-8.44	
ENSG00000272589	-8.44	ZSWIM8-AS1

ENSG00000274666	-8.44	
ENSG00000166821	-8.44	PEX11A
ENSG00000232021	-8.43	LEF1-AS1
ENSG00000227431	-8.43	CSE1L-AS1
ENSG00000286032	-8.43	
ENSG00000233706	-8.40	
ENSG00000152404	-8.38	CWF19L2
ENSG00000100532	-8.36	CGRRF1
ENSG00000134668	-8.34	SPOCD1
ENSG00000204758	-8.34	LOC100268168
ENSG00000163331	-8.31	DAPL1
ENSG00000239626	-8.31	
ENSG00000171469	-8.30	ZNF561
ENSG00000258413	-8.28	
ENSG00000166896	-8.26	ATP23
ENSG00000180596	-8.25	HIST1H2BC
ENSG00000151062	-8.24	CACNA2D4
ENSG00000242992	-8.21	
ENSG00000270154	-8.21	
ENSG00000124785	-8.16	NRN1
ENSG00000224891	-8.13	
ENSG00000255867	-8.10	DENND5B-AS1
ENSG00000249768	-8.09	
ENSG00000259316	-8.03	
ENSG00000233175	-8.03	
ENSG00000106415	-7.98	GLCCI1
ENSG00000256482	-7.98	
ENSG00000127720	-7.97	METTL25
ENSG00000248932	-7.96	LOC100507291
ENSG00000112245	-7.93	PTP4A1
ENSG00000134326	-7.92	CMPK2
ENSG00000205076	-7.92	LGALS7
ENSG00000198857	-7.87	
ENSG00000273272	-7.85	
ENSG00000219790	-7.77	
ENSG00000279064	-7.77	
ENSG00000101928	-7.71	MOSPD1
ENSG00000104327	-7.70	CALB1
ENSG00000182459	-7.68	TEX19
ENSG00000134532	-7.67	SOX5
ENSG00000244229	-7.67	
ENSG00000273080	-7.67	

ENSG00000171843	-7.66	MLLT3
ENSG00000243871	-7.65	
ENSG00000285822	-7.65	
ENSG00000116035	-7.62	VAX2
ENSG00000223726	-7.62	
ENSG00000257139	-7.62	
ENSG00000272950	-7.62	
ENSG00000254609	-7.60	
ENSG00000239671	-7.58	
ENSG00000254388	-7.57	
ENSG00000264273	-7.57	
ENSG00000267016	-7.56	
ENSG00000139921	-7.55	TMX1
ENSG00000113621	-7.54	TXNDC15
ENSG00000198464	-7.54	ZNF480
ENSG00000178115	-7.53	
ENSG00000103599	-7.52	IQCH
ENSG00000255530	-7.52	
ENSG00000184502	-7.51	GAST
ENSG00000253934	-7.51	
ENSG00000286024	-7.50	
ENSG00000275638	-7.48	
ENSG00000203812	-7.48	HIST2H2AA3
ENSG00000095261	-7.47	PSMD5
ENSG00000134317	-7.46	GRHL1
ENSG00000129467	-7.46	ADCY4
ENSG00000271199	-7.46	
ENSG00000242748	-7.45	
ENSG00000253167	-7.43	
ENSG00000011052	-7.38	NME1-NME2
ENSG00000240356	-7.38	
ENSG00000272837	-7.38	
ENSG00000230175	-7.35	
ENSG00000234789	-7.35	
ENSG00000255126	-7.35	
ENSG00000283496	-7.33	
ENSG00000101353	-7.32	MROH8
ENSG00000128512	-7.32	DOCK4
ENSG00000229153	-7.32	EPHA1-AS1
ENSG00000230799	-7.30	
ENSG00000104613	-7.30	INTS10
ENSG00000278275	-7.29	

ENSG00000259181	-7.26	
ENSG00000236451	-7.24	
ENSG00000168803	-7.23	ADAL
ENSG00000255566	-7.22	
ENSG00000084453	-7.21	SLCO1A2
ENSG00000203778	-7.21	FAM229B
ENSG00000234925	-7.20	
ENSG00000254860	-7.18	TMEM9B-AS1
ENSG00000186998	-7.17	EMID1
ENSG00000075711	-7.17	DLG1
ENSG00000104147	-7.15	OIP5
ENSG00000150337	-7.15	FCGR1A
ENSG00000081870	-7.14	HSPB11
ENSG00000227354	-7.13	RBM26-AS1
ENSG00000163235	-7.11	TGFA
ENSG00000138050	-7.11	THUMPD2
ENSG00000240695	-7.08	
ENSG00000156253	-7.07	RWDD2B
ENSG00000286140	-7.05	
ENSG00000287349	-7.03	
ENSG00000165837	-7.02	ERICH6B
ENSG00000182185	-7.01	RAD51B
ENSG00000172456	-6.98	FGGY
ENSG00000173867	-6.98	
ENSG00000205084	-6.97	TMEM231
ENSG00000124701	-6.96	APOBEC2
ENSG00000196368	-6.96	NUDT11
ENSG00000233834	-6.95	LOC100506098
ENSG00000164045	-6.95	CDC25A
ENSG00000163352	-6.94	LENEP
ENSG00000188100	-6.89	FAM25A
ENSG00000128656	-6.88	CHN1
ENSG00000248564	-6.87	
ENSG00000270733	-6.84	
ENSG00000236682	-6.82	
ENSG00000241288	-6.81	LOC101927056
ENSG00000103546	-6.81	SLC6A2
ENSG00000286688	-6.79	
ENSG00000135437	-6.79	RDH5
ENSG00000198105	-6.79	ZNF248
ENSG00000124839	-6.78	RAB17
ENSG00000189050	-6.78	RNFT1

ENSG00000232627	-6.78	
ENSG00000158636	-6.78	EMSY
ENSG00000205572	-6.77	SERF1B
ENSG00000286216	-6.74	
ENSG00000143815	-6.73	LBR
ENSG00000281333	-6.72	
ENSG00000263235	-6.71	
ENSG00000055163	-6.71	CYFIP2
ENSG00000223774	-6.69	
ENSG00000242941	-6.69	
ENSG00000250476	-6.69	
ENSG00000272081	-6.69	
ENSG00000255920	-6.69	
ENSG00000132837	-6.68	DMGDH
ENSG00000245598	-6.64	DACT3-AS1
ENSG00000157927	-6.63	RADIL
ENSG00000275506	-6.63	
ENSG00000110811	-6.62	P3H3
ENSG00000106069	-6.60	LOC101928168
ENSG00000225981	-6.59	
ENSG00000155111	-6.58	CDK19
ENSG00000115363	-6.57	EVA1A
ENSG00000248668	-6.57	OXCT1-AS1
ENSG00000135870	-6.57	RC3H1
ENSG00000105926	-6.55	MPP6
ENSG00000189114	-6.55	BLOC1S3
ENSG00000203724	-6.53	C1orf53
ENSG00000143994	-6.52	ABHD1
ENSG00000235897	-6.52	TM4SF19-AS1
ENSG00000250604	-6.49	
ENSG00000255038	-6.49	
ENSG00000260657	-6.49	
ENSG00000267280	-6.48	TBX2-AS1
ENSG00000274315	-6.47	
ENSG00000113594	-6.44	LIFR
ENSG00000257636	-6.42	
ENSG00000106692	-6.42	FKTN
ENSG00000099377	-6.40	HSD3B7
ENSG00000215915	-6.40	ATAD3C
ENSG00000224842	-6.40	
ENSG00000169474	-6.40	SPRR1A
ENSG00000104756	-6.40	KCTD9

ENSG00000258655	-6.38		
ENSG00000259984	-6.34		
ENSG00000169439	-6.31	SDC2	
ENSG00000253944	-6.31		
ENSG00000262482	-6.31		
ENSG00000100867	-6.31	DHRS2	
ENSG00000125354	-6.30		6-Sep
ENSG00000247708	-6.30	STX18-AS1	
ENSG00000259407	-6.29		
ENSG00000163960	-6.29	UBXN7	
ENSG00000243927	-6.28	MRPS6	
ENSG00000101888	-6.27	NXT2	
ENSG00000076242	-6.26	MLH1	
ENSG00000120526	-6.26	NUDCD1	
ENSG00000225573	-6.26		
ENSG00000237130	-6.26		
ENSG00000260469	-6.25		
ENSG00000119537	-6.24	KDSR	
ENSG00000134709	-6.24	HOOK1	
ENSG00000233586	-6.24		
ENSG00000067066	-6.23	SP100	
ENSG00000183655	-6.23	KLHL25	
ENSG00000104723	-6.22	TUSC3	
ENSG00000279568	-6.22		
ENSG00000138767	-6.22	CNOT6L	
ENSG00000280354	-6.22		
ENSG00000164953	-6.21	TMEM67	
ENSG00000130844	-6.21	ZNF331	
ENSG00000049089	-6.18	COL9A2	
ENSG00000187260	-6.17	WDR86	
ENSG00000275484	-6.16		
ENSG00000264458	-6.13		
ENSG00000058804	-6.13	NDC1	
ENSG00000198915	-6.12	RASGEF1A	
ENSG00000231616	-6.12		
ENSG00000255237	-6.12		
ENSG00000024862	-6.10	CCDC28A	
ENSG00000242808	-6.10		
ENSG00000172014	-6.09	ANKRD20A4	
ENSG00000183148	-6.09	ANKRD20A2	
ENSG00000242553	-6.09		
ENSG00000015568	-6.08	RGPD5	

ENSG00000243236	-6.07	
ENSG00000274031	-6.07	
ENSG00000262302	-6.06	
ENSG00000237550	-6.06	
ENSG00000128482	-6.06	RNF112
ENSG00000273261	-6.05	
ENSG00000070729	-6.05	CNGB1
ENSG00000224618	-6.05	
ENSG00000241680	-6.05	
ENSG00000260466	-6.05	
ENSG00000275549	-6.03	STPG3-AS1
ENSG00000179094	-6.02	PER1
ENSG00000213598	-6.01	
ENSG00000238123	-6.01	
ENSG00000103707	-6.00	MTFMT
ENSG00000142252	-5.97	GEMIN7
ENSG00000271032	-5.97	
ENSG00000235823	-5.97	
ENSG00000118513	-5.95	MYB
ENSG00000263326	-5.95	
ENSG00000267246	-5.95	
ENSG00000057019	-5.94	DCBLD2
ENSG00000178295	-5.94	GEN1
ENSG00000160870	-5.93	CYP3A7
ENSG00000276649	-5.93	
ENSG00000113407	-5.93	TARS
ENSG00000279813	-5.92	
ENSG00000104626	-5.91	ERI1
ENSG00000267150	-5.90	
ENSG00000171224	-5.89	C10orf35
ENSG00000187815	-5.88	ZFP69
ENSG00000230979	-5.87	
ENSG00000025156	-5.87	HSF2
ENSG00000137871	-5.86	LOC145783
ENSG00000257379	-5.86	
ENSG00000142279	-5.85	WTIP
ENSG00000155085	-5.85	AK9
ENSG00000269915	-5.85	
ENSG00000279621	-5.84	
ENSG00000173728	-5.83	C1orf100
ENSG00000261070	-5.83	LOC338694
ENSG00000186687	-5.83	LYRM7

ENSG00000146006	-5.82	LRRTM2
ENSG00000231840	-5.81	
ENSG00000281649	-5.80	EBLN3P
ENSG00000106460	-5.77	TMEM106B
ENSG00000273306	-5.75	
ENSG00000131730	-5.74	CKMT2
ENSG00000285953	-5.73	
ENSG00000083099	-5.73	LYRM2
ENSG00000104219	-5.72	ZDHHC2
ENSG00000166225	-5.72	FRS2
ENSG00000214940	-5.72	NPIPA8
ENSG00000038945	-5.71	MSR1
ENSG00000117069	-5.71	ST6GALNAC5
ENSG00000100483	-5.71	VCPKMT
ENSG00000118322	-5.70	ATP10B
ENSG00000227253	-5.70	
ENSG00000240793	-5.70	
ENSG00000146263	-5.68	MMS22L
ENSG00000163539	-5.68	CLASP2
ENSG00000213277	-5.68	
ENSG00000259479	-5.68	
ENSG00000128604	-5.67	IRF5
ENSG00000205634	-5.66	LINC00898
ENSG00000272362	-5.65	
ENSG00000135740	-5.63	SLC9A5
ENSG00000280284	-5.63	
ENSG00000126698	-5.63	DNAJC8
ENSG00000164683	-5.62	HEY1
ENSG00000116117	-5.61	PARD3B
ENSG00000122507	-5.60	BBS9
ENSG00000253829	-5.60	
ENSG00000270050	-5.60	
ENSG00000179119	-5.59	SPTY2D1
ENSG00000250053	-5.59	
ENSG00000123607	-5.58	TTC21B
ENSG00000215302	-5.58	
ENSG00000282964	-5.58	
ENSG00000239828	-5.57	
ENSG00000135164	-5.57	DMTF1
ENSG00000189067	-5.54	LITAF
ENSG00000178150	-5.54	ZNF114
ENSG00000248103	-5.54	

ENSG00000261613	-5.52	
ENSG00000272716	-5.51	
ENSG00000287817	-5.51	
ENSG00000078319	-5.50	
ENSG00000198791	-5.50	CNOT7
ENSG00000213648	-5.50	SULT1A4
ENSG00000243961	-5.50	
ENSG00000271049	-5.50	
ENSG00000100296	-5.50	THOC5
ENSG00000233868	-5.49	
ENSG00000250994	-5.49	
ENSG00000260115	-5.49	
ENSG00000270108	-5.49	
ENSG00000135211	-5.48	TMEM60
ENSG00000266909	-5.47	
ENSG00000280026	-5.46	
ENSG00000166479	-5.45	TMX3
ENSG00000283515	-5.43	
ENSG00000234665	-5.42	
ENSG00000148200	-5.40	NR6A1
ENSG00000272897	-5.40	
ENSG00000154146	-5.39	NRGN
ENSG00000147010	-5.39	SH3KBP1
ENSG00000227212	-5.38	
ENSG00000258090	-5.38	
ENSG00000160221	-5.37	C21orf33
ENSG00000158163	-5.37	DZIP1L
ENSG00000189149	-5.37	
ENSG00000149090	-5.36	PAMR1
ENSG00000266728	-5.36	
ENSG00000149591	-5.35	TAGLN
ENSG00000067992	-5.35	PDK3
ENSG00000101898	-5.35	
ENSG00000206562	-5.35	METTL6
ENSG00000287851	-5.35	
ENSG00000099810	-5.34	MTAP
ENSG00000189376	-5.33	C8orf76
ENSG00000112029	-5.33	FBXO5
ENSG00000170417	-5.33	TMEM182
ENSG00000138642	-5.33	HERC6
ENSG00000215492	-5.33	
ENSG00000274949	-5.33	

ENSG00000282772	-5.33	
ENSG00000168172	-5.32	HOOK3
ENSG00000175899	-5.32	A2M
ENSG00000131558	-5.30	EXOC4
ENSG00000227973	-5.30	
ENSG00000271914	-5.30	
ENSG00000119640	-5.30	ACYP1
ENSG00000106540	-5.29	
ENSG00000244490	-5.28	
ENSG00000273174	-5.28	
ENSG00000278367	-5.28	
ENSG00000229124	-5.27	VIM-AS1
ENSG00000118503	-5.26	TNFAIP3
ENSG00000256894	-5.26	
ENSG00000133119	-5.26	RFC3
ENSG00000151881	-5.25	TMEM267
ENSG00000162694	-5.25	EXTL2
ENSG00000148357	-5.24	HMCN2
ENSG00000219700	-5.24	
ENSG00000269533	-5.24	
ENSG00000179387	-5.23	ELMOD2
ENSG00000250910	-5.23	LOC102724776
ENSG00000183258	-5.22	DDX41
ENSG00000005059	-5.22	MCUB
ENSG00000259544	-5.22	
ENSG00000166478	-5.21	ZNF143
ENSG00000089169	-5.20	RPH3A
ENSG00000244045	-5.20	TMEM199
ENSG00000277150	-5.19	F8A3
ENSG00000163617	-5.19	CCDC191
ENSG00000225230	-5.18	
ENSG00000115425	-5.18	PECR
ENSG00000181804	-5.17	SLC9A9
ENSG00000248956	-5.17	
ENSG00000267980	-5.15	
ENSG00000085365	-5.14	SCAMP1
ENSG00000222020	-5.14	LOC101928111
ENSG00000255666	-5.14	
ENSG00000270006	-5.14	LOC101928659
ENSG00000176485	-5.14	PLA2G16
ENSG00000276850	-5.13	
ENSG00000286847	-5.13	

ENSG00000269072	-5.11	LOC101928517
ENSG00000172379	-5.11	ARNT2
ENSG00000203804	-5.10	ADAMTSL4-AS1
ENSG00000145331	-5.09	TRMT10A
ENSG00000232640	-5.09	
ENSG00000167183	-5.08	PRR15L
ENSG00000267033	-5.08	
ENSG00000283991	-5.08	
ENSG00000131437	-5.08	KIF3A
ENSG00000258951	-5.08	
ENSG00000230804	-5.07	
ENSG00000261669	-5.07	
ENSG00000277147	-5.07	LINC00869
ENSG00000011201	-5.06	ANOS1
ENSG00000130517	-5.06	PGPEP1
ENSG00000269929	-5.05	
ENSG00000154359	-5.05	LONRF1
ENSG00000287929	-5.05	
ENSG00000263394	-5.04	
ENSG00000116106	-5.03	EPHA4
ENSG00000271581	-5.03	
ENSG00000166596	-5.03	CFAP52
ENSG00000236117	-5.02	
ENSG00000112761	-5.01	WISP3
ENSG00000253708	-5.01	
ENSG00000104901	-5.00	DKKL1
ENSG00000144935	-5.00	TRPC1
ENSG00000228919	-5.00	
ENSG00000227540	-4.99	
ENSG00000248578	-4.99	
ENSG00000170365	-4.99	SMAD1
ENSG00000083535	-4.99	PIBF1
ENSG00000284830	-4.98	
ENSG00000005238	-4.97	FAM214B
ENSG00000163913	-4.97	IFT122
ENSG00000136011	-4.97	STAB2
ENSG00000227400	-4.97	
ENSG00000250645	-4.97	
ENSG00000197312	-4.97	DDI2
ENSG00000197969	-4.96	VPS13A
ENSG00000260661	-4.96	
ENSG00000254469	-4.96	

ENSG00000170836	-4.95	PPM1D
ENSG00000254929	-4.95	
ENSG00000183734	-4.95	ASCL2
ENSG00000248664	-4.95	
ENSG00000203785	-4.95	SPRR2E
ENSG00000272787	-4.95	
ENSG00000102098	-4.94	SCML2
ENSG00000253716	-4.94	MINCR
ENSG00000224511	-4.94	LINC00365
ENSG00000141576	-4.93	RNF157
ENSG00000113578	-4.93	FGF1
ENSG00000213872	-4.92	
ENSG00000248101	-4.92	
ENSG00000232931	-4.91	
ENSG00000227788	-4.91	
ENSG00000228340	-4.91	MIR646HG
ENSG00000245322	-4.91	LOC256880
ENSG00000274471	-4.91	
ENSG00000213020	-4.91	ZNF611
ENSG00000224049	-4.91	
ENSG00000180189	-4.90	
ENSG00000229167	-4.90	
ENSG00000255330	-4.90	
ENSG00000179833	-4.89	SERTAD2
ENSG00000175575	-4.89	PAAF1
ENSG00000229474	-4.88	PATL2
ENSG00000162616	-4.88	DNAJB4
ENSG00000054219	-4.87	LY75
ENSG00000207955	-4.87	
ENSG00000239906	-4.87	
ENSG00000233381	-4.87	
ENSG00000226407	-4.86	
ENSG00000228624	-4.86	HDAC2-AS2
ENSG00000180155	-4.85	
ENSG00000227578	-4.85	
ENSG00000280018	-4.85	
ENSG00000134250	-4.85	NOTCH2
ENSG00000225032	-4.83	LOC102723566
ENSG00000144635	-4.82	DYNC1LI1
ENSG00000217289	-4.81	
ENSG00000224067	-4.81	
ENSG00000182287	-4.81	AP1S2

ENSG00000135406	-4.80	PRPH
ENSG00000265908	-4.79	
ENSG00000267390	-4.79	
ENSG00000137878	-4.79	GCOM1
ENSG00000260651	-4.78	
ENSG00000125462	-4.78	C1orf61
ENSG00000128881	-4.77	TTBK2
ENSG00000214146	-4.77	LINC02026
ENSG00000164031	-4.77	DNAJB14
ENSG00000066651	-4.76	TRMT11
ENSG00000238188	-4.74	
ENSG00000160471	-4.73	COX6B2
ENSG00000223976	-4.73	
ENSG00000227008	-4.73	
ENSG00000225205	-4.73	
ENSG00000114383	-4.72	TUSC2
ENSG00000156500	-4.72	FAM122C
ENSG00000286742	-4.72	
ENSG00000254509	-4.70	
ENSG00000145743	-4.70	FBXL17
ENSG00000235677	-4.70	
ENSG00000104368	-4.69	PLAT
ENSG00000228335	-4.69	
ENSG00000233297	-4.69	
ENSG00000267141	-4.69	
ENSG00000196302	-4.68	
ENSG00000262362	-4.68	
ENSG00000155016	-4.67	CYP2U1
ENSG00000215533	-4.67	LINC00189
ENSG00000237923	-4.67	LOC105375014
ENSG00000251484	-4.67	
ENSG00000114268	-4.67	PFKFB4
ENSG00000186765	-4.66	FSCN2
ENSG00000226688	-4.64	ENTPD1-AS1
ENSG00000231024	-4.64	
ENSG00000167912	-4.64	LOC100505501
ENSG00000152443	-4.64	ZNF776
ENSG00000260498	-4.63	
ENSG00000269026	-4.63	
ENSG00000280832	-4.63	
ENSG00000288640	-4.63	
ENSG00000248487	-4.63	ABHD14A

ENSG00000235091	-4.62	
ENSG00000131019	-4.61	ULBP3
ENSG00000237870	-4.61	LOC102724434
ENSG00000174099	-4.60	MSRB3
ENSG00000250107	-4.60	CACNA1G-AS1
ENSG00000178761	-4.59	FAM219B
ENSG00000187534	-4.59	
ENSG00000236663	-4.59	FRGCA
ENSG00000267643	-4.59	
ENSG00000083720	-4.57	OXCT1
ENSG00000228630	-4.57	HOTAIR
ENSG00000133114	-4.57	GPALPP1
ENSG00000215018	-4.57	COL28A1
ENSG00000111801	-4.56	BTN3A3
ENSG00000234515	-4.56	
ENSG00000274712	-4.56	
ENSG00000079308	-4.56	TNS1
ENSG00000139890	-4.55	REM2
ENSG00000008294	-4.55	SPAG9
ENSG00000174628	-4.55	IQCK
ENSG00000226874	-4.54	
ENSG00000135698	-4.54	MPHOSPH6
ENSG00000181619	-4.54	GPR135
ENSG00000240204	-4.54	SMKR1
ENSG00000257000	-4.54	
ENSG00000279529	-4.53	
ENSG00000146409	-4.53	SLC18B1
ENSG00000184205	-4.52	TSPYL2
ENSG00000125246	-4.52	CLYBL
ENSG00000152056	-4.52	AP1S3
ENSG00000132432	-4.52	SEC61G
ENSG00000148516	-4.52	ZEB1
ENSG00000165113	-4.52	GKAP1
ENSG00000184144	-4.52	CNTN2
ENSG00000138678	-4.52	GPAT3
ENSG00000165996	-4.52	HACD1
ENSG00000156531	-4.52	PHF6
ENSG00000101751	-4.52	POLI
ENSG00000122694	-4.51	GLIPR2
ENSG00000250682	-4.50	
ENSG00000196748	-4.49	CLPSL2
ENSG00000150527	-4.48	

ENSG00000264837	-4.48	
ENSG00000184113	-4.47	CLDN5
ENSG00000259954	-4.47	IL21R-AS1
ENSG00000261706	-4.47	
ENSG00000267049	-4.47	
ENSG00000183077	-4.46	AFMID
ENSG00000104450	-4.46	SPAG1
ENSG00000198553	-4.46	KCNRG
ENSG00000136045	-4.45	PWP1
ENSG00000230021	-4.44	LOC101928626
ENSG00000256417	-4.44	
ENSG00000278942	-4.43	
ENSG00000119950	-4.42	MXI1
ENSG00000219891	-4.42	
ENSG00000143514	-4.42	TP53BP2
ENSG00000167355	-4.42	
ENSG00000259498	-4.42	
ENSG00000145431	-4.41	PDGFC
ENSG00000254527	-4.41	
ENSG00000067533	-4.41	RRP15
ENSG00000216775	-4.39	LOC730101
ENSG00000111186	-4.39	WNT5B
ENSG00000171570	-4.39	
ENSG00000215874	-4.39	
ENSG00000258515	-4.38	
ENSG00000184349	-4.38	EFNA5
ENSG00000260170	-4.38	
ENSG00000260231	-4.38	JHDM1D-AS1
ENSG00000237438	-4.38	CECR7
ENSG00000196912	-4.37	ANKRD36B
ENSG00000285331	-4.37	
ENSG00000071575	-4.37	TRIB2
ENSG00000095485	-4.37	CWF19L1
ENSG00000248125	-4.36	
ENSG00000243056	-4.35	EIF4EBP3
ENSG00000248866	-4.34	USP46-AS1
ENSG00000264007	-4.34	
ENSG00000170266	-4.33	GLB1
ENSG00000163389	-4.33	POGLUT1
ENSG00000197467	-4.33	COL13A1
ENSG00000287005	-4.33	
ENSG00000271969	-4.32	

ENSG00000186205	-4.32		1-Mar
ENSG00000170456	-4.31	DENND5B	
ENSG00000186951	-4.31	PPARA	
ENSG00000254034	-4.31		
ENSG00000186615	-4.31	KTN1-AS1	
ENSG00000175832	-4.31	ETV4	
ENSG00000170498	-4.30	KISS1	
ENSG00000230311	-4.30		
ENSG00000230659	-4.30		
ENSG00000236745	-4.30		
ENSG00000240288	-4.30	GHRLOS	
ENSG00000137814	-4.30	HAUS2	
ENSG00000198393	-4.29	ZNF26	
ENSG00000137868	-4.29	STRA6	
ENSG00000235590	-4.29	GNAS-AS1	
ENSG00000113456	-4.28	RAD1	
ENSG00000175877	-4.28	TMEM270	
ENSG00000176371	-4.28	ZSCAN2	
ENSG00000230433	-4.28		
ENSG00000286244	-4.28		
ENSG00000230426	-4.26		
ENSG00000109046	-4.26	WSB1	
ENSG00000127578	-4.26	WFIKKN1	
ENSG00000257740	-4.26		
ENSG00000121486	-4.25	TRMT1L	
ENSG00000168491	-4.24	CCDC110	
ENSG00000226221	-4.24		
ENSG00000230102	-4.24	LINC02028	
ENSG00000241494	-4.24		
ENSG00000260882	-4.24		
ENSG00000247675	-4.23	LRP4-AS1	
ENSG00000091831	-4.23	ESR1	
ENSG00000235837	-4.23		
ENSG00000287839	-4.23		
ENSG00000165338	-4.22	HECTD2	
ENSG00000254489	-4.22	LOC105376609	
ENSG00000280113	-4.22		
ENSG00000248401	-4.21		
ENSG00000275756	-4.21		
ENSG00000168890	-4.21	TMEM150A	
ENSG00000170855	-4.21	TRIAP1	
ENSG00000139656	-4.21	SMIM2	

ENSG00000157017	-4.20	GHRL
ENSG00000259116	-4.20	LOC102723809
ENSG00000124243	-4.19	BCAS4
ENSG00000244578	-4.19	LINC01391
ENSG00000286319	-4.19	
ENSG00000099910	-4.19	KLHL22
ENSG00000153130	-4.19	SCOC
ENSG00000227262	-4.19	
ENSG00000247728	-4.18	
ENSG00000231851	-4.18	UTAT33
ENSG00000229539	-4.18	
ENSG00000267117	-4.18	
ENSG00000185946	-4.18	RNPC3
ENSG00000284060	-4.18	
ENSG00000271360	-4.18	
ENSG00000225606	-4.17	
ENSG00000146530	-4.17	VWDE
ENSG00000240086	-4.17	
ENSG00000260923	-4.17	
ENSG00000261329	-4.17	
ENSG00000136197	-4.16	C7orf25
ENSG00000170248	-4.16	PDCD6IP
ENSG00000137726	-4.16	FXYD6
ENSG00000260747	-4.16	
ENSG00000243004	-4.16	
ENSG00000153291	-4.15	SLC25A27
ENSG00000197442	-4.15	MAP3K5
ENSG00000100918	-4.13	REC8
ENSG00000133661	-4.13	SFTPD
ENSG00000264269	-4.13	
ENSG00000138778	-4.13	CENPE
ENSG00000117724	-4.12	CENPF
ENSG00000187824	-4.12	TMEM220
ENSG00000157578	-4.11	LCA5L
ENSG00000140598	-4.11	EFL1
ENSG00000261011	-4.11	
ENSG00000258945	-4.10	
ENSG00000161544	-4.10	CYGB
ENSG00000261242	-4.10	
ENSG00000083642	-4.08	PDS5B
ENSG00000140262	-4.08	TCF12
ENSG00000099338	-4.08	CATSPERG

ENSG00000153933	-4.08	DGKE
ENSG00000203684	-4.08	IBA57-AS1
ENSG00000235529	-4.08	AGAP1-IT1
ENSG00000270022	-4.08	
ENSG00000165410	-4.08	CFL2
ENSG00000160117	-4.07	ANKLE1
ENSG00000255571	-4.07	MIR9-3HG
ENSG00000257964	-4.06	
ENSG00000005469	-4.06	CROT
ENSG00000144182	-4.06	LIPT1
ENSG00000244380	-4.06	
ENSG00000250999	-4.06	
ENSG00000253754	-4.06	
ENSG00000237686	-4.06	LOC101929705
ENSG00000260081	-4.06	LOC105373383
ENSG00000206344	-4.05	HCG27
ENSG00000089250	-4.05	NOS1
ENSG00000272971	-4.05	
ENSG00000254485	-4.05	
ENSG00000241438	-4.05	
ENSG00000248593	-4.05	DSTNP2
ENSG00000262648	-4.05	
ENSG00000272430	-4.05	
ENSG00000116761	-4.04	CTH
ENSG00000177494	-4.03	ZBED2
ENSG00000174796	-4.03	THAP6
ENSG00000145284	-4.02	SCD5
ENSG00000272155	-4.02	
ENSG00000138757	-4.02	G3BP2
ENSG00000104047	-4.01	DTWD1
ENSG00000225513	-4.01	
ENSG00000260145	-4.01	
ENSG00000142871	-4.00	CYR61
ENSG00000261872	-4.00	
ENSG00000282757	-4.00	
ENSG00000136935	-3.99	GOLGA1
ENSG00000228906	-3.99	
ENSG00000276368	-3.99	HIST1H2AJ
ENSG00000079215	-3.98	SLC1A3
ENSG00000160460	-3.98	SPTBN4
ENSG00000023287	-3.97	RB1CC1
ENSG00000163515	-3.97	RETNLB

ENSG00000197258	-3.97	
ENSG00000188243	-3.96	COMMD6
ENSG00000196951	-3.96	SCOC-AS1
ENSG00000225526	-3.96	MKRN2OS
ENSG00000256087	-3.96	ZNF432
ENSG00000227741	-3.95	LOC729867
ENSG00000245849	-3.94	RAD51-AS1
ENSG00000158246	-3.94	FAM46B
ENSG00000257921	-3.94	
ENSG00000113448	-3.94	PDE4D
ENSG00000164902	-3.94	PHAX
ENSG00000206341	-3.93	
ENSG00000274859	-3.93	
ENSG00000135116	-3.93	HRK
ENSG00000143452	-3.93	HORMAD1
ENSG00000227473	-3.93	
ENSG00000146587	-3.92	RBAK
ENSG00000224387	-3.92	
ENSG00000227542	-3.92	
ENSG00000258846	-3.92	
ENSG00000272205	-3.92	
ENSG00000164362	-3.91	TERT
ENSG00000115145	-3.90	STAM2
ENSG00000230679	-3.90	ENO1-AS1
ENSG00000197948	-3.89	FCHSD1
ENSG00000171658	-3.89	NMRAL2P
ENSG00000187164	-3.89	SHTN1
ENSG00000160606	-3.89	TLCD1
ENSG00000196505	-3.89	GDAP2
ENSG00000223508	-3.89	
ENSG00000232508	-3.89	
ENSG00000149311	-3.89	ATM
ENSG00000232852	-3.88	
ENSG00000271576	-3.88	
ENSG00000129071	-3.88	MBD4
ENSG00000160282	-3.87	FTCD
ENSG00000235315	-3.87	
ENSG00000069482	-3.87	GAL
ENSG00000165568	-3.86	AKR1E2
ENSG00000106006	-3.86	HOXA6
ENSG00000163002	-3.86	NUP35
ENSG00000167325	-3.86	RRM1

ENSG00000266978	-3.86	
ENSG00000287624	-3.86	
ENSG00000066117	-3.85	SMARCD1
ENSG00000047249	-3.85	ATP6V1H
ENSG00000070950	-3.85	RAD18
ENSG00000268307	-3.85	
ENSG00000278083	-3.85	
ENSG00000286682	-3.85	
ENSG00000180806	-3.85	HOXC9
ENSG00000181873	-3.85	IBA57
ENSG00000275582	-3.85	
ENSG00000236503	-3.84	
ENSG00000136490	-3.84	LIMD2
ENSG00000196214	-3.84	ZNF766
ENSG00000101343	-3.83	CRNKL1
ENSG00000233483	-3.83	
ENSG00000100593	-3.83	ISM2
ENSG00000158806	-3.83	NPM2
ENSG00000196935	-3.83	SRGAP1
ENSG00000179477	-3.82	ALOX12B
ENSG00000109667	-3.82	SLC2A9
ENSG00000163154	-3.82	TNFAIP8L2
ENSG00000280721	-3.82	
ENSG00000232850	-3.81	PTGES2-AS1
ENSG00000176124	-3.81	DLEU1
ENSG00000131152	-3.81	
ENSG00000227195	-3.81	MIR663AHG
ENSG00000104081	-3.81	BMF
ENSG00000215819	-3.81	
ENSG00000162148	-3.80	PPP1R32
ENSG00000225171	-3.80	
ENSG00000250349	-3.80	
ENSG00000254480	-3.80	LOC100506082
ENSG00000272509	-3.80	
ENSG00000273267	-3.80	
ENSG00000144746	-3.79	ARL6IP5
ENSG00000130203	-3.79	APOE
ENSG00000223886	-3.79	
ENSG00000230673	-3.79	
ENSG00000231910	-3.79	
ENSG00000153044	-3.79	CENPH
ENSG00000196950	-3.78	SLC39A10

ENSG00000241749	-3.78	RPSAP52
ENSG00000263934	-3.78	SNORD3A
ENSG00000272368	-3.78	
ENSG00000227321	-3.78	
ENSG00000269897	-3.78	COMMD3-BMI1
ENSG00000102038	-3.77	SMARCA1
ENSG00000223749	-3.77	MIR503HG
ENSG00000239556	-3.77	
ENSG00000244712	-3.77	
ENSG00000253540	-3.77	
ENSG00000255557	-3.77	
ENSG00000277368	-3.77	
ENSG00000130649	-3.76	CYP2E1
ENSG00000233040	-3.76	
ENSG00000012660	-3.76	ELOVL5
ENSG00000204147	-3.76	ASAH2B
ENSG00000229227	-3.76	
ENSG00000066827	-3.76	ZFAT
ENSG00000237382	-3.75	
ENSG00000224897	-3.74	POT1-AS1
ENSG00000135521	-3.74	LTV1
ENSG00000232625	-3.72	
ENSG00000272760	-3.72	
ENSG00000115325	-3.72	DOK1
ENSG00000231752	-3.71	EMBP1
ENSG00000137815	-3.71	RTF1
ENSG00000164061	-3.71	BSN
ENSG00000205584	-3.71	
ENSG00000231767	-3.71	
ENSG00000267472	-3.71	
ENSG00000198315	-3.70	ZKSCAN8
ENSG00000159674	-3.70	SPON2
ENSG00000241990	-3.70	
ENSG00000163815	-3.70	CLEC3B
ENSG00000264569	-3.70	
ENSG00000182578	-3.69	CSF1R
ENSG00000183598	-3.69	HIST2H3D
ENSG00000204789	-3.69	
ENSG00000256678	-3.69	
ENSG00000272720	-3.69	
ENSG00000286342	-3.69	
ENSG00000215251	-3.68	FASTKD5

ENSG00000143751	-3.68	SDE2
ENSG00000286485	-3.68	
ENSG00000234682	-3.68	
ENSG00000241728	-3.68	
ENSG00000174015	-3.68	SPERT
ENSG00000172465	-3.67	TCEAL1
ENSG00000117472	-3.67	TSPAN1
ENSG00000132846	-3.67	ZBED3
ENSG00000092096	-3.67	SLC22A17
ENSG00000276185	-3.67	
ENSG00000173239	-3.67	LIPM
ENSG00000182853	-3.67	VMO1
ENSG00000231942	-3.67	
ENSG00000259171	-3.67	
ENSG00000274020	-3.67	LINC01138
ENSG00000074266	-3.66	EED
ENSG00000163072	-3.66	NOSTRIN
ENSG00000070526	-3.66	ST6GALNAC1
ENSG00000184898	-3.66	RBM43
ENSG00000248405	-3.66	PRR5-ARHGAP8
ENSG00000131400	-3.66	NAPSA
ENSG00000170579	-3.65	DLGAP1
ENSG00000249148	-3.65	
ENSG00000004777	-3.65	ARHGAP33
ENSG00000159256	-3.64	MORC3
ENSG00000198087	-3.64	CD2AP
ENSG00000140284	-3.64	SLC27A2
ENSG00000251359	-3.64	WWC2-AS2
ENSG00000227769	-3.63	
ENSG00000243960	-3.63	
ENSG00000279989	-3.63	
ENSG00000148814	-3.63	LRRC27
ENSG00000196565	-3.62	HBG2
ENSG00000115290	-3.62	GRB14
ENSG00000259212	-3.62	
ENSG00000266969	-3.62	
ENSG00000123836	-3.62	PFKFB2
ENSG00000284906	-3.61	
ENSG00000179528	-3.61	LBX2
ENSG00000242173	-3.60	ARHGDIG
ENSG00000138459	-3.60	SLC35A5
ENSG00000151553	-3.60	FAM160B1

ENSG00000282121	-3.60	
ENSG00000042088	-3.59	TDP1
ENSG00000162757	-3.59	C1orf74
ENSG00000239713	-3.59	APOBEC3G
ENSG00000253907	-3.59	
ENSG00000183520	-3.59	UTP11
ENSG00000272836	-3.59	
ENSG00000251136	-3.58	LOC101929709
ENSG00000174485	-3.58	DENND4A
ENSG00000181135	-3.58	LOC101928160
ENSG00000164211	-3.58	STARD4
ENSG00000179299	-3.58	NSUN7
ENSG00000181450	-3.58	ZNF678
ENSG00000228477	-3.58	
ENSG00000237080	-3.58	
ENSG00000148019	-3.58	CEP78
ENSG00000073605	-3.58	GSDMB
ENSG00000228384	-3.57	
ENSG00000286433	-3.57	
ENSG00000230316	-3.57	FEZF1-AS1
ENSG00000050393	-3.57	MCUR1
ENSG00000171466	-3.57	ZNF562
ENSG00000269956	-3.56	MKNK1-AS1
ENSG00000168016	-3.56	TRANK1
ENSG00000164744	-3.56	SUN3
ENSG00000225133	-3.56	
ENSG00000250917	-3.56	
ENSG00000280212	-3.56	
ENSG00000150873	-3.55	C2orf50
ENSG00000240053	-3.55	
ENSG00000101082	-3.54	SLA2
ENSG00000220472	-3.54	
ENSG00000267395	-3.54	
ENSG00000232757	-3.53	
ENSG00000268655	-3.53	LOC101059948
ENSG00000270094	-3.53	
ENSG00000170356	-3.53	OR2A20P
ENSG00000111666	-3.52	CHPT1
ENSG00000177197	-3.52	
ENSG00000196741	-3.52	
ENSG00000185630	-3.51	PBX1
ENSG00000172728	-3.51	FUT10

ENSG00000101844	-3.51	ATG4A
ENSG00000133739	-3.51	LRRCC1
ENSG00000103160	-3.51	HSDL1
ENSG00000162813	-3.51	BPNT1
ENSG00000188818	-3.50	ZDHHC11
ENSG00000224081	-3.50	SLC44A3-AS1
ENSG00000220392	-3.50	
ENSG00000286366	-3.50	
ENSG00000091136	-3.49	LAMB1
ENSG00000180176	-3.49	TH
ENSG00000231007	-3.49	
ENSG00000261575	-3.49	
ENSG00000270580	-3.49	PKD1P6-NPIPP1
ENSG00000076555	-3.49	ACACB
ENSG00000121964	-3.49	GTDC1
ENSG00000237523	-3.48	LINC00857
ENSG00000243649	-3.48	CFB
ENSG00000152766	-3.48	ANKRD22
ENSG00000153714	-3.48	LURAP1L
ENSG00000228113	-3.48	
ENSG00000268047	-3.48	
ENSG00000280420	-3.48	
ENSG00000165861	-3.48	ZFYVE1
ENSG00000126067	-3.48	PSMB2
ENSG00000128254	-3.47	C22orf24
ENSG00000131401	-3.47	NAPSB
ENSG00000178691	-3.47	SUZ12
ENSG00000198860	-3.47	TSEN15
ENSG00000119801	-3.46	YPEL5
ENSG00000089682	-3.46	RBM41
ENSG00000121210	-3.46	TMEM131L
ENSG00000237982	-3.46	
ENSG00000255118	-3.46	
ENSG00000270690	-3.46	
ENSG00000272432	-3.46	
ENSG00000272853	-3.46	
ENSG00000284707	-3.46	
ENSG00000142583	-3.45	SLC2A5
ENSG00000162654	-3.45	GBP4
ENSG00000287080	-3.45	
ENSG00000011376	-3.45	LARS2
ENSG00000086666	-3.44	ZFAND6

ENSG00000176142	-3.44	TMEM39A
ENSG00000112357	-3.44	PEX7
ENSG00000221829	-3.44	FANCG
ENSG00000155313	-3.44	USP25
ENSG00000149557	-3.44	FEZ1
ENSG00000174292	-3.43	TNK1
ENSG00000143924	-3.43	EML4
ENSG00000109445	-3.43	ZNF330
ENSG00000025423	-3.43	HSD17B6
ENSG00000048540	-3.43	LMO3
ENSG00000171766	-3.43	GATM
ENSG00000198353	-3.43	HOXC4
ENSG00000285106	-3.43	
ENSG00000285583	-3.43	
ENSG00000180385	-3.43	
ENSG00000198121	-3.42	LPAR1
ENSG00000139354	-3.42	GAS2L3
ENSG00000112599	-3.41	GUCA1B
ENSG00000225265	-3.41	TAF1A-AS1
ENSG00000242267	-3.41	
ENSG00000239257	-3.41	
ENSG00000050130	-3.41	JKAMP
ENSG00000134253	-3.41	TRIM45
ENSG00000178425	-3.41	NT5DC1
ENSG00000100731	-3.41	PCNX1
ENSG00000069493	-3.40	CLEC2D
ENSG00000153006	-3.40	SREK1IP1
ENSG00000177627	-3.40	C12orf54
ENSG00000224321	-3.40	
ENSG00000231858	-3.40	LOC105373805
ENSG00000280062	-3.40	
ENSG00000076770	-3.40	MBNL3
ENSG00000260558	-3.39	
ENSG00000238245	-3.39	
ENSG00000257438	-3.39	
ENSG00000002549	-3.38	LAP3
ENSG00000197446	-3.38	CYP2F1
ENSG00000254109	-3.38	RBPMS-AS1
ENSG00000041982	-3.38	TNC
ENSG00000258012	-3.38	
ENSG00000260628	-3.38	
ENSG00000134243	-3.37	SORT1

ENSG00000253492	-3.37	
ENSG00000254335	-3.37	
ENSG00000261625	-3.37	
ENSG00000226009	-3.36	KCNIP2-AS1
ENSG00000204396	-3.36	VWA7
ENSG00000259514	-3.36	
ENSG00000285943	-3.36	
ENSG00000137806	-3.36	NDUFAF1
ENSG00000260430	-3.36	
ENSG00000165714	-3.36	BORCS5
ENSG00000257831	-3.35	
ENSG00000224975	-3.35	INE1
ENSG00000275778	-3.35	PRH1-PRR4
ENSG00000137760	-3.35	ALKBH8
ENSG00000129534	-3.35	MIS18BP1
ENSG00000176225	-3.35	RTTN
ENSG00000213614	-3.35	HEXA
ENSG00000250425	-3.34	
ENSG00000164056	-3.34	SPRY1
ENSG00000177058	-3.34	SLC38A9
ENSG00000234835	-3.34	
ENSG00000253959	-3.34	
ENSG00000258510	-3.34	
ENSG00000259363	-3.34	LOC400464
ENSG00000285292	-3.34	
ENSG00000286181	-3.34	
ENSG00000236266	-3.33	
ENSG00000109819	-3.33	PPARGC1A
ENSG00000159208	-3.33	CIART
ENSG00000179593	-3.33	ALOX15B
ENSG00000225913	-3.33	
ENSG00000227220	-3.33	
ENSG00000233237	-3.33	
ENSG00000261089	-3.33	
ENSG00000285733	-3.33	
ENSG00000241322	-3.32	CDRT1
ENSG00000254814	-3.32	
ENSG00000162852	-3.32	CNST
ENSG00000123119	-3.32	NECAB1
ENSG00000253172	-3.32	
ENSG00000166167	-3.31	BTRC
ENSG00000177133	-3.31	LINC00982

ENSG00000224752	-3.31	
ENSG00000234645	-3.31	
ENSG00000254037	-3.31	
ENSG00000260622	-3.31	
ENSG00000162971	-3.31	TYW5
ENSG00000275902	-3.30	
ENSG00000206195	-3.30	DUXAP8
ENSG00000151876	-3.30	FBXO4
ENSG00000138604	-3.30	GLCE
ENSG00000226883	-3.30	
ENSG00000260571	-3.30	
ENSG00000281207	-3.30	SLFNL1-AS1
ENSG00000251483	-3.30	
ENSG00000134954	-3.30	ETS1
ENSG00000287407	-3.30	
ENSG00000236319	-3.29	
ENSG00000257103	-3.29	LSM14A
ENSG00000257605	-3.29	
ENSG00000251675	-3.29	
ENSG00000272825	-3.29	
ENSG00000224046	-3.29	LOC101927420
ENSG00000285632	-3.29	
ENSG00000283486	-3.29	
ENSG00000282416	-3.29	
ENSG00000166387	-3.28	PPFIBP2
ENSG00000198909	-3.28	MAP3K3
ENSG00000274227	-3.28	
ENSG00000233956	-3.28	
ENSG00000242220	-3.28	TCP10L
ENSG00000277981	-3.28	
ENSG00000144681	-3.28	STAC
ENSG00000186141	-3.28	POLR3C
ENSG00000234797	-3.28	
ENSG00000182177	-3.27	ASB18
ENSG00000244586	-3.27	
ENSG00000246695	-3.27	
ENSG00000266775	-3.27	
ENSG00000269859	-3.27	
ENSG00000079482	-3.27	OPHN1
ENSG00000254560	-3.27	BBOX1-AS1
ENSG00000246067	-3.26	RAB30-AS1
ENSG00000228008	-3.26	

ENSG00000109775	-3.26	UFSP2
ENSG00000085433	-3.26	WDR47
ENSG00000103522	-3.26	IL21R
ENSG00000109072	-3.26	VTN
ENSG00000159387	-3.26	IRX6
ENSG00000172339	-3.26	ALG14
ENSG00000139737	-3.26	SLAIN1
ENSG00000108666	-3.25	C17orf75
ENSG00000198677	-3.25	TTC37
ENSG00000176401	-3.25	EID2B
ENSG00000240694	-3.25	PNMA2
ENSG00000264304	-3.25	
ENSG00000146476	-3.24	ARMT1
ENSG00000128218	-3.24	VPREB3
ENSG00000214447	-3.24	
ENSG00000223802	-3.24	CERS1
ENSG00000232912	-3.24	
ENSG00000253258	-3.24	
ENSG00000266970	-3.24	LOC101928674
ENSG00000273049	-3.24	
ENSG00000277332	-3.24	LOC101928087
ENSG00000068654	-3.24	POLR1A
ENSG00000103978	-3.24	TMEM87A
ENSG00000231087	-3.23	
ENSG00000237356	-3.23	
ENSG00000259553	-3.23	
ENSG00000266053	-3.23	NDUFV2-AS1
ENSG00000259155	-3.23	
ENSG00000089177	-3.23	KIF16B
ENSG00000122970	-3.22	IFT81
ENSG00000249087	-3.22	ZNF436-AS1
ENSG00000231887	-3.22	PRH1
ENSG00000182118	-3.22	FAM89A
ENSG00000135537	-3.22	AFG1L
ENSG00000219435	-3.22	CATSPERZ
ENSG00000267871	-3.22	LOC105372476
ENSG00000109063	-3.21	MYH3
ENSG00000144231	-3.21	POLR2D
ENSG00000255959	-3.21	
ENSG00000125378	-3.21	BMP4
ENSG00000168811	-3.21	IL12A
ENSG00000214389	-3.21	

ENSG00000287908	-3.21	
ENSG00000154153	-3.21	RETREG1
ENSG00000132746	-3.21	ALDH3B2
ENSG00000151743	-3.20	AMN1
ENSG00000240445	-3.20	
ENSG00000248131	-3.20	
ENSG00000226864	-3.20	ATE1-AS1
ENSG00000254429	-3.20	
ENSG00000244313	-3.20	
ENSG00000175324	-3.20	LSM1
ENSG00000204176	-3.20	SYT15
ENSG00000143919	-3.19	CAMKMT
ENSG00000229273	-3.19	
ENSG00000139223	-3.19	ANP32D
ENSG00000177699	-3.19	
ENSG00000236546	-3.19	LOC105378668
ENSG00000267122	-3.19	
ENSG00000101639	-3.19	CEP192
ENSG00000261320	-3.18	
ENSG00000048342	-3.18	CC2D2A
ENSG00000082515	-3.18	MRPL22
ENSG00000111452	-3.18	ADGRD1
ENSG00000070915	-3.18	SLC12A3
ENSG00000231310	-3.18	
ENSG00000254907	-3.18	
ENSG00000273117	-3.18	
ENSG00000288258	-3.18	
ENSG00000076321	-3.18	KLHL20
ENSG00000218510	-3.17	LINC00339
ENSG00000227409	-3.17	
ENSG00000168661	-3.17	ZNF30
ENSG00000227245	-3.17	
ENSG00000260236	-3.17	
ENSG00000140481	-3.17	CCDC33
ENSG00000091947	-3.17	TMEM101
ENSG00000127947	-3.16	PTPN12
ENSG00000115239	-3.16	ASB3
ENSG00000151572	-3.16	ANO4
ENSG00000286138	-3.16	
ENSG00000287781	-3.16	
ENSG00000178093	-3.16	TSSK6
ENSG00000180336	-3.15	MEIOC

ENSG00000197301	-3.15	LOC100129940
ENSG00000116641	-3.15	DOCK7
ENSG00000101158	-3.15	NELFCD
ENSG00000185267	-3.15	CDNF
ENSG00000230395	-3.15	
ENSG00000265939	-3.15	
ENSG00000183763	-3.15	TRAIP
ENSG00000163257	-3.15	DCAF16
ENSG00000149573	-3.14	MPZL2
ENSG00000133983	-3.14	COX16
ENSG00000197813	-3.14	
ENSG00000198947	-3.14	DMD
ENSG00000237575	-3.14	PYY2
ENSG00000247774	-3.14	PCED1B-AS1
ENSG00000258359	-3.14	
ENSG00000267898	-3.14	
ENSG00000268584	-3.14	
ENSG00000255503	-3.14	
ENSG00000213022	-3.14	
ENSG00000162928	-3.14	PEX13
ENSG00000128805	-3.13	ARHGAP22
ENSG00000280893	-3.13	
ENSG00000104324	-3.13	CPQ
ENSG00000044459	-3.13	CNTLN
ENSG00000108370	-3.13	RGS9
ENSG00000180611	-3.13	MB21D2
ENSG00000235934	-3.13	
ENSG00000172530	-3.12	BANP
ENSG00000102804	-3.12	TSC22D1
ENSG00000079313	-3.12	REXO1
ENSG00000163728	-3.12	TTC14
ENSG00000010810	-3.12	FYN
ENSG00000061455	-3.12	PRDM6
ENSG00000171811	-3.12	CFAP46
ENSG00000266709	-3.12	MGC12916
ENSG00000005249	-3.12	PRKAR2B
ENSG00000184613	-3.12	NELL2
ENSG00000158019	-3.12	BABAM2
ENSG00000248008	-3.11	NRAV
ENSG00000171448	-3.11	ZBTB26
ENSG00000133460	-3.11	SLC2A11
ENSG00000183196	-3.11	CHST6

ENSG00000227197	-3.11	
ENSG00000237872	-3.11	POU5F1P4
ENSG00000250501	-3.11	
ENSG00000251330	-3.11	
ENSG00000250786	-3.10	SNHG18
ENSG00000279863	-3.10	
ENSG00000221914	-3.10	PPP2R2A
ENSG00000279584	-3.10	
ENSG00000286616	-3.10	
ENSG00000145725	-3.10	PPIP5K2
ENSG00000130177	-3.10	CDC16
ENSG00000042317	-3.10	SPATA7
ENSG00000071073	-3.09	MGAT4A
ENSG00000116819	-3.09	TFAP2E
ENSG00000122592	-3.09	HOXA7
ENSG00000267291	-3.09	LOC105372397
ENSG00000286811	-3.09	
ENSG00000168925	-3.08	CTRB1
ENSG00000239322	-3.08	ATP6V1B1-AS1
ENSG00000258900	-3.08	
ENSG00000262115	-3.08	
ENSG00000267261	-3.08	
ENSG00000286864	-3.08	
ENSG00000113300	-3.08	CNOT6
ENSG00000137831	-3.07	UACA
ENSG00000107201	-3.07	DDX58
ENSG00000233708	-3.07	
ENSG00000241720	-3.07	
ENSG00000259594	-3.07	
ENSG00000089685	-3.07	BIRC5
ENSG00000239827	-3.06	SUGT1P3
ENSG00000250240	-3.06	
ENSG00000197580	-3.06	BCO2
ENSG00000132196	-3.06	HSD17B7
ENSG00000131966	-3.06	ACTR10
ENSG00000182973	-3.05	CNOT10
ENSG00000196436	-3.05	NPIPB15
ENSG00000196502	-3.05	SULT1A1
ENSG00000149346	-3.05	SLX4IP
ENSG00000255046	-3.05	
ENSG00000274383	-3.05	
ENSG00000274904	-3.05	

ENSG00000286696	-3.05	
ENSG00000163867	-3.05	ZMYM6
ENSG00000143951	-3.05	WDPCP
ENSG00000231290	-3.05	APCDD1L-AS1
ENSG00000115935	-3.05	WIPF1
ENSG00000166444	-3.04	ST5
ENSG00000118785	-3.04	SPP1
ENSG00000253981	-3.04	
ENSG00000150907	-3.03	FOXO1
ENSG00000050438	-3.03	SLC4A8
ENSG00000197165	-3.03	SULT1A2
ENSG00000224837	-3.03	
ENSG00000258733	-3.03	
ENSG00000115364	-3.03	MRPL19
ENSG00000247081	-3.03	BAALC-AS1
ENSG00000111780	-3.02	
ENSG00000245573	-3.02	BDNF-AS
ENSG00000255856	-3.02	
ENSG00000255983	-3.02	
ENSG00000259673	-3.02	IQCH-AS1
ENSG00000286129	-3.02	
ENSG00000168556	-3.02	ING2
ENSG00000272600	-3.01	
ENSG00000015479	-3.01	
ENSG00000165055	-3.01	METTL2B
ENSG00000164253	-3.01	WDR41
ENSG00000133657	-3.01	ATP13A3
ENSG00000143776	-3.01	CDC42BPA
ENSG00000141255	-3.01	SPATA22
ENSG00000188223	-3.01	
ENSG00000197535	-3.01	MYO5A
ENSG00000122299	-3.01	ZC3H7A
ENSG00000141540	-3.00	TTYH2
ENSG00000174032	-3.00	SLC25A30
ENSG00000217801	-3.00	LOC100288175
ENSG00000215193	-2.99	PEX26
ENSG00000166173	-2.99	LARP6
ENSG00000135436	-2.99	FAM186B
ENSG00000178074	-2.99	C2orf69
ENSG00000070031	-2.99	SCT
ENSG00000137673	-2.99	MMP7
ENSG00000168918	-2.99	INPP5D

ENSG00000176510	-2.99	
ENSG00000240747	-2.99	KRBOX1
ENSG00000267699	-2.99	
ENSG00000271484	-2.99	
ENSG00000273925	-2.99	
ENSG00000073712	-2.99	FERMT2
ENSG00000266820	-2.99	
ENSG00000183155	-2.99	RABIF
ENSG00000100462	-2.98	PRMT5
ENSG00000234851	-2.98	
ENSG00000277778	-2.98	PGM5P2
ENSG00000236671	-2.98	
ENSG00000116704	-2.98	SLC35D1
ENSG00000144908	-2.98	ALDH1L1
ENSG00000180694	-2.98	TMEM64
ENSG00000184616	-2.98	
ENSG00000189057	-2.98	FAM111B
ENSG00000280087	-2.98	
ENSG00000096060	-2.98	FKBP5
ENSG00000169118	-2.97	CSNK1G1
ENSG00000151466	-2.97	SCLT1
ENSG00000140563	-2.97	MCTP2
ENSG00000242622	-2.97	
ENSG00000247735	-2.97	
ENSG00000183486	-2.97	MX2
ENSG00000225791	-2.97	TRAM2-AS1
ENSG00000269813	-2.97	
ENSG00000285879	-2.97	
ENSG00000153976	-2.97	HS3ST3A1
ENSG00000242242	-2.96	
ENSG00000167904	-2.96	TMEM68
ENSG00000267123	-2.96	LINC02081
ENSG00000272573	-2.96	MUSTN1
ENSG00000174939	-2.96	ASPHD1
ENSG00000268350	-2.96	FAM156A
ENSG00000105219	-2.96	CNTD2
ENSG00000080986	-2.96	NDC80
ENSG00000064393	-2.96	HIPK2
ENSG00000126524	-2.95	SBDS
ENSG00000241472	-2.95	PTPRG-AS1
ENSG00000128928	-2.95	IVD
ENSG00000236542	-2.95	

ENSG00000264204	-2.95	
ENSG00000260280	-2.95	SLX1B-SULT1A4
ENSG00000167306	-2.95	MYO5B
ENSG00000161647	-2.94	MPP3
ENSG00000115368	-2.94	WDR75
ENSG00000093100	-2.94	
ENSG00000128917	-2.94	DLL4
ENSG00000213620	-2.94	
ENSG00000259528	-2.94	
ENSG00000269091	-2.94	
ENSG00000181433	-2.94	SAGE1
ENSG00000244625	-2.94	
ENSG00000275457	-2.94	
ENSG00000167281	-2.93	RBFOX3
ENSG00000055732	-2.93	MCOLN3
ENSG00000261687	-2.93	
ENSG00000273300	-2.93	
ENSG00000159263	-2.93	SIM2
ENSG00000169136	-2.93	ATF5
ENSG00000180370	-2.93	PAK2
ENSG00000196352	-2.92	CD55
ENSG00000128607	-2.92	KLHDC10
ENSG00000128683	-2.92	GAD1
ENSG00000250295	-2.92	RDH10-AS1
ENSG00000156958	-2.92	GALK2
ENSG00000188833	-2.92	ENTPD8
ENSG00000213185	-2.91	FAM24B
ENSG00000171608	-2.91	PIK3CD
ENSG00000162344	-2.91	FGF19
ENSG00000121289	-2.91	CEP89
ENSG00000114670	-2.91	NEK11
ENSG00000143036	-2.91	SLC44A3
ENSG00000166275	-2.91	
ENSG00000230778	-2.91	ANKRD63
ENSG00000174136	-2.91	RGMB
ENSG00000163964	-2.91	PIGX
ENSG00000280287	-2.91	
ENSG00000117697	-2.91	NSL1
ENSG00000225764	-2.90	P3H2-AS1
ENSG00000240441	-2.90	
ENSG00000258420	-2.90	
ENSG00000272226	-2.90	

ENSG00000280183	-2.90	
ENSG00000089505	-2.90	CMTM1
ENSG00000173273	-2.90	TNKS
ENSG00000196497	-2.90	IPO4
ENSG00000198824	-2.90	CHAMP1
ENSG00000255882	-2.89	
ENSG00000110107	-2.89	PRPF19
ENSG00000164338	-2.89	UTP15
ENSG00000165060	-2.89	FXN
ENSG00000178750	-2.89	STX19
ENSG00000262171	-2.89	
ENSG00000279971	-2.89	
ENSG00000131037	-2.89	EPS8L1
ENSG00000104687	-2.89	GSR
ENSG00000275936	-2.89	
ENSG00000088179	-2.89	PTPN4
ENSG00000112759	-2.88	SLC29A1
ENSG00000163517	-2.88	HDAC11
ENSG00000254618	-2.88	
ENSG00000138798	-2.88	EGF
ENSG00000213625	-2.88	LEPROT
ENSG00000214671	-2.88	
ENSG00000228544	-2.88	CCDC183-AS1
ENSG00000262943	-2.88	ALOX12P2
ENSG00000139211	-2.88	AMIGO2
ENSG00000105137	-2.87	SYDE1
ENSG00000225889	-2.87	
ENSG00000171130	-2.87	ATP6V0E2
ENSG00000182366	-2.87	FAM87A
ENSG00000160746	-2.87	ANO10
ENSG00000112977	-2.87	DAP
ENSG00000102158	-2.87	MAGT1
ENSG00000169570	-2.87	DTWD2
ENSG00000154309	-2.86	DISP1
ENSG00000215859	-2.86	
ENSG00000183242	-2.86	WT1-AS
ENSG00000253991	-2.86	
ENSG00000259548	-2.86	
ENSG00000237886	-2.86	
ENSG00000051382	-2.86	PIK3CB
ENSG00000256469	-2.86	
ENSG00000227693	-2.85	

ENSG00000186352	-2.85	ANKRD37
ENSG00000233296	-2.85	LOC105373354
ENSG00000239636	-2.85	
ENSG00000269044	-2.85	
ENSG00000164961	-2.85	WASHC5
ENSG00000260978	-2.85	
ENSG00000099250	-2.84	NRP1
ENSG00000179256	-2.84	SMCO3
ENSG00000205981	-2.84	DNAJC19
ENSG00000122687	-2.83	MRM2
ENSG00000213190	-2.83	MLLT11
ENSG00000169604	-2.83	ANTXR1
ENSG00000214357	-2.83	NEURL1B
ENSG00000287710	-2.83	
ENSG00000186230	-2.83	ZNF749
ENSG00000265415	-2.83	
ENSG00000185669	-2.83	SNAI3
ENSG00000258177	-2.83	
ENSG00000088836	-2.82	SLC4A11
ENSG00000284024	-2.82	
ENSG00000106952	-2.82	TNFSF8
ENSG00000109738	-2.82	GLRB
ENSG00000244371	-2.82	
ENSG00000260874	-2.82	LOC105371050
ENSG00000261251	-2.82	
ENSG00000283378	-2.82	LOC100289279
ENSG00000287742	-2.82	
ENSG00000153885	-2.82	KCTD15
ENSG00000119203	-2.81	CPSF3
ENSG00000171217	-2.81	CLDN20
ENSG00000109534	-2.81	GAR1
ENSG00000226251	-2.81	LOC101929541
ENSG00000277511	-2.81	
ENSG00000118515	-2.81	SGK1
ENSG00000270629	-2.80	LOC100996763
ENSG00000124772	-2.80	CPNE5
ENSG00000186417	-2.80	GLDN
ENSG00000198919	-2.80	DZIP3
ENSG00000273226	-2.80	
ENSG00000186867	-2.80	QRFPR
ENSG00000180229	-2.80	HERC2P3
ENSG00000185829	-2.79	ARL17A

ENSG00000259529	-2.79	
ENSG00000135912	-2.79	TTLL4
ENSG00000168405	-2.79	
ENSG00000186088	-2.79	GSAP
ENSG00000198208	-2.79	RPS6KL1
ENSG00000269275	-2.79	
ENSG00000165264	-2.79	NDUFB6
ENSG00000230087	-2.79	
ENSG00000167748	-2.78	KLK1
ENSG00000188315	-2.78	C3orf62
ENSG00000240395	-2.78	
ENSG00000250451	-2.78	HOXC-AS1
ENSG00000288067	-2.78	
ENSG00000114279	-2.78	FGF12
ENSG00000204899	-2.77	MZT1
ENSG00000241015	-2.77	TPM3P9
ENSG00000205213	-2.77	LGR4
ENSG00000094975	-2.77	SUCO
ENSG00000019102	-2.77	VSIG2
ENSG00000113209	-2.77	PCDHB5
ENSG00000120500	-2.77	ARR3
ENSG00000188396	-2.77	TCTEX1D4
ENSG00000197302	-2.77	LOC107983990
ENSG00000204620	-2.77	
ENSG00000224079	-2.77	
ENSG00000230417	-2.77	LINC00856
ENSG00000251259	-2.77	
ENSG00000185238	-2.77	PRMT3
ENSG00000151612	-2.77	ZNF827
ENSG00000227370	-2.77	
ENSG00000182584	-2.77	ACTL10
ENSG00000184261	-2.77	KCNK12
ENSG00000114745	-2.77	GORASP1
ENSG00000265148	-2.76	TSPOAP1-AS1
ENSG00000281327	-2.76	LINC01338
ENSG00000258891	-2.76	
ENSG00000144959	-2.76	NCEH1
ENSG00000265474	-2.76	
ENSG00000131788	-2.76	PIAS3
ENSG00000108384	-2.76	RAD51C
ENSG00000228343	-2.76	
ENSG00000221886	-2.76	ZBED8

ENSG00000251188	-2.76	
ENSG00000283541	-2.76	
ENSG00000119446	-2.76	RBM18
ENSG00000125772	-2.75	GPCPD1
ENSG00000165125	-2.75	TRPV6
ENSG00000273149	-2.75	
ENSG00000139675	-2.75	HNRNPA1L2
ENSG00000174951	-2.75	FUT1
ENSG00000178917	-2.75	ZNF852
ENSG00000172345	-2.75	STARD5
ENSG00000134222	-2.75	PSRC1
ENSG00000064205	-2.75	WISP2
ENSG00000167637	-2.75	ZNF283
ENSG00000237264	-2.75	
ENSG00000254870	-2.75	ATP6V1G2-DDX39B
ENSG00000278918	-2.75	
ENSG00000231721	-2.75	LINC-PINT
ENSG00000266086	-2.75	
ENSG00000137267	-2.75	TUBB2A
ENSG00000162961	-2.74	DPY30
ENSG00000198736	-2.74	MSRB1
ENSG00000215270	-2.74	
ENSG00000135838	-2.74	NPL
ENSG00000106633	-2.74	GCK
ENSG00000138381	-2.74	ASNSD1
ENSG00000174640	-2.74	SLCO2A1
ENSG00000230408	-2.74	LOC101927795
ENSG00000232110	-2.74	
ENSG00000250041	-2.74	
ENSG00000259031	-2.74	
ENSG00000261367	-2.74	
ENSG00000263393	-2.74	
ENSG00000203999	-2.74	LINC01270
ENSG00000138182	-2.73	KIF20B
ENSG00000251034	-2.73	
ENSG00000100934	-2.73	SEC23A
ENSG00000087301	-2.73	TXNDC16
ENSG00000138152	-2.73	BTBD16
ENSG00000139985	-2.73	ADAM21
ENSG00000255225	-2.73	
ENSG00000161057	-2.73	PSMC2
ENSG00000142197	-2.73	DOPEY2

ENSG00000267374	-2.73	MIR924HG
ENSG00000167394	-2.73	ZNF668
ENSG00000158156	-2.73	XKR8
ENSG00000101493	-2.73	ZNF516
ENSG00000254706	-2.73	
ENSG00000259132	-2.72	
ENSG00000267160	-2.72	
ENSG00000159784	-2.72	FAM131B
ENSG00000167632	-2.72	TRAPPC9
ENSG00000088876	-2.72	ZNF343
ENSG00000127124	-2.72	HIVEP3
ENSG00000256349	-2.72	
ENSG00000258701	-2.72	LINC00638
ENSG00000280206	-2.72	
ENSG00000281706	-2.72	LINC01012
ENSG00000197043	-2.72	ANXA6
ENSG00000162591	-2.72	MEGF6
ENSG00000144283	-2.72	PKP4
ENSG00000025039	-2.71	RRAGD
ENSG00000167985	-2.71	
ENSG00000196843	-2.71	ARID5A
ENSG00000213513	-2.71	
ENSG00000259365	-2.71	
ENSG00000108423	-2.71	TUBD1
ENSG00000093009	-2.71	CDC45
ENSG00000188612	-2.71	SUMO2
ENSG00000108389	-2.71	MTMR4
ENSG00000139899	-2.71	CBLN3
ENSG00000267680	-2.70	ZNF224
ENSG00000231970	-2.70	
ENSG00000237286	-2.70	
ENSG00000125352	-2.70	RNF113A
ENSG00000079101	-2.70	CLUL1
ENSG00000155792	-2.70	DEPTOR
ENSG00000229190	-2.70	
ENSG00000243979	-2.70	
ENSG00000244968	-2.70	LIFR-AS1
ENSG00000250532	-2.70	
ENSG00000266283	-2.70	
ENSG00000227766	-2.70	
ENSG00000250714	-2.69	
ENSG00000166501	-2.69	PRKCB

ENSG00000181355	-2.69	OFCC1
ENSG00000188981	-2.69	MSANTD1
ENSG00000267048	-2.69	
ENSG00000282418	-2.69	
ENSG00000134852	-2.69	CLOCK
ENSG00000181610	-2.69	MRPS23
ENSG00000146411	-2.69	SLC2A12
ENSG00000263020	-2.69	
ENSG00000118971	-2.68	CCND2
ENSG00000135535	-2.68	CD164
ENSG00000151233	-2.68	GXYLT1
ENSG00000164164	-2.68	OTUD4
ENSG00000270147	-2.68	
ENSG00000105088	-2.68	OLFM2
ENSG00000268565	-2.68	
ENSG00000033170	-2.68	FUT8
ENSG00000077232	-2.68	DNAJC10
ENSG00000152256	-2.67	PDK1
ENSG00000171101	-2.67	SIGLEC17P
ENSG00000250722	-2.67	SELENOP
ENSG00000134899	-2.67	ERCC5
ENSG00000111860	-2.67	CEP85L
ENSG00000213707	-2.67	
ENSG00000215790	-2.67	SLC35E2
ENSG00000279878	-2.67	
ENSG00000184160	-2.67	ADRA2C
ENSG00000164105	-2.67	SAP30
ENSG00000262528	-2.66	
ENSG00000135638	-2.66	EMX1
ENSG00000156113	-2.66	KCNMA1
ENSG00000184304	-2.66	PRKD1
ENSG00000187140	-2.66	FOXD3
ENSG00000213069	-2.66	
ENSG00000204380	-2.66	PKP4-AS1
ENSG00000187554	-2.65	TLR5
ENSG00000112365	-2.65	ZBTB24
ENSG00000196636	-2.65	SDHAF3
ENSG00000214113	-2.65	LYRM4
ENSG00000234964	-2.65	
ENSG00000273398	-2.65	
ENSG00000265749	-2.65	
ENSG00000058063	-2.65	ATP11B

ENSG00000141570	-2.64	CBX8
ENSG00000260304	-2.64	
ENSG00000155367	-2.64	PPM1J
ENSG00000248268	-2.64	
ENSG00000251301	-2.64	LINC02384
ENSG00000259657	-2.64	
ENSG00000287454	-2.64	
ENSG00000167670	-2.64	CHAF1A
ENSG00000116906	-2.64	GNPAT
ENSG00000137200	-2.64	CMTR1
ENSG00000167925	-2.63	GHDC
ENSG00000268606	-2.63	MAGEA2
ENSG00000119408	-2.63	NEK6
ENSG00000243708	-2.63	PLA2G4B
ENSG00000228109	-2.63	MELTF-AS1
ENSG00000273559	-2.63	CWC25
ENSG00000068615	-2.63	REEP1
ENSG00000163499	-2.63	CRYBA2
ENSG00000185513	-2.63	L3MBTL1
ENSG00000228126	-2.63	FALEC
ENSG00000258983	-2.63	
ENSG00000262061	-2.63	LOC100506388
ENSG00000175274	-2.63	TP53I11
ENSG00000263528	-2.63	IKBKE
ENSG00000181852	-2.62	RNF41
ENSG00000168477	-2.62	TNXB
ENSG00000006634	-2.62	DBF4
ENSG00000171873	-2.62	ADRA1D
ENSG00000183647	-2.62	ZNF530
ENSG00000224426	-2.62	
ENSG00000265126	-2.62	
ENSG00000267112	-2.62	
ENSG00000270832	-2.62	
ENSG00000131094	-2.62	C1QL1
ENSG00000143157	-2.62	POGK
ENSG00000142733	-2.62	MAP3K6
ENSG00000180846	-2.62	CSNK1G2-AS1
ENSG00000065717	-2.62	TLE2
ENSG00000224424	-2.61	PRKAR2A-AS1
ENSG00000158089	-2.61	GALNT14
ENSG00000178177	-2.61	LCORL
ENSG00000281128	-2.61	PTENP1-AS

ENSG00000134324	-2.61	LPIN1
ENSG00000196338	-2.61	NLGN3
ENSG00000229207	-2.61	
ENSG00000249412	-2.61	
ENSG00000267631	-2.61	CGB1
ENSG00000166482	-2.61	MFAP4
ENSG00000104043	-2.61	ATP8B4
ENSG00000124813	-2.61	RUNX2
ENSG00000164070	-2.61	HSPA4L
ENSG00000125657	-2.61	TNFSF9
ENSG00000141569	-2.60	TRIM65
ENSG00000116001	-2.60	TIA1
ENSG00000103365	-2.60	GGA2
ENSG00000010318	-2.60	PHF7
ENSG00000141449	-2.60	GREB1L
ENSG00000261488	-2.60	
ENSG00000138772	-2.60	ANXA3
ENSG00000265168	-2.60	
ENSG00000052723	-2.60	SIKE1
ENSG00000137076	-2.60	TLN1
ENSG00000257056	-2.60	LINC02282
ENSG00000185480	-2.60	PARPBP
ENSG00000086848	-2.60	ALG9
ENSG00000022267	-2.59	FHL1
ENSG00000095585	-2.59	BLNK
ENSG00000229380	-2.59	LOC101927000
ENSG00000236204	-2.59	LINC01376
ENSG00000260409	-2.59	
ENSG00000266777	-2.59	
ENSG00000164221	-2.59	CCDC112
ENSG00000123485	-2.59	HJURP
ENSG00000125430	-2.58	HS3ST3B1
ENSG00000249395	-2.58	
ENSG00000131477	-2.58	RAMP2
ENSG00000188766	-2.58	SPRED3
ENSG00000227684	-2.58	
ENSG00000237934	-2.58	
ENSG00000075213	-2.58	SEMA3A
ENSG00000121989	-2.58	ACVR2A
ENSG00000151461	-2.57	UPF2
ENSG00000062716	-2.57	VMP1
ENSG00000133624	-2.57	ZNF767P

ENSG00000113790	-2.57	EHHADH
ENSG00000134533	-2.57	RERG
ENSG00000145375	-2.57	SPATA5
ENSG00000163739	-2.57	CXCL1
ENSG00000216331	-2.57	
ENSG00000267481	-2.57	
ENSG00000196290	-2.57	NIF3L1
ENSG00000170837	-2.57	GPR27
ENSG00000103528	-2.56	SYT17
ENSG00000189369	-2.56	GSPT2
ENSG00000106588	-2.56	
ENSG00000023608	-2.56	SNAPC1
ENSG00000064199	-2.56	SPA17
ENSG00000188816	-2.56	HMX2
ENSG00000224789	-2.56	
ENSG00000227702	-2.56	
ENSG00000261713	-2.56	SSTR5-AS1
ENSG00000267890	-2.56	
ENSG00000140968	-2.56	IRF8
ENSG00000156162	-2.56	DPY19L4
ENSG00000164284	-2.55	GRPEL2
ENSG00000130348	-2.55	QRSL1
ENSG00000233360	-2.55	LOC101927051
ENSG00000102096	-2.55	PIM2
ENSG00000168490	-2.55	PHYHIP
ENSG00000094755	-2.55	GABRP
ENSG00000105672	-2.55	ETV2
ENSG00000239323	-2.55	
ENSG00000254419	-2.55	
ENSG00000182518	-2.55	FAM104B
ENSG00000118017	-2.55	A4GNT
ENSG00000149809	-2.55	TM7SF2
ENSG00000117114	-2.55	ADGRL2
ENSG00000184979	-2.54	USP18
ENSG00000234409	-2.54	CCDC188
ENSG00000138594	-2.54	TMOD3
ENSG00000090487	-2.54	SPG21
ENSG00000092470	-2.54	WDR76
ENSG00000244556	-2.54	
ENSG00000196284	-2.54	SUPT3H
ENSG00000134256	-2.54	CD101
ENSG00000171522	-2.54	PTGER4

ENSG00000174672	-2.54	BRSK2
ENSG00000197405	-2.54	C5AR1
ENSG00000236308	-2.54	
ENSG00000143889	-2.54	HNRNPLL
ENSG00000215883	-2.54	CYB5RL
ENSG00000184432	-2.54	COPB2
ENSG00000273802	-2.53	HIST1H2BG
ENSG00000012174	-2.53	MBTPS2
ENSG00000229376	-2.53	
ENSG00000258876	-2.53	
ENSG00000226363	-2.53	
ENSG00000173338	-2.53	KCNK7
ENSG00000228917	-2.52	
ENSG00000177570	-2.52	SAMD12
ENSG00000105427	-2.52	CNFN
ENSG00000091490	-2.52	SEL1L3
ENSG00000211455	-2.52	STK38L
ENSG00000128276	-2.52	RFPL3
ENSG00000268403	-2.52	LOC644656
ENSG00000198658	-2.52	
ENSG00000253659	-2.52	
ENSG00000257501	-2.52	LOC643711
ENSG00000148700	-2.52	ADD3
ENSG00000211456	-2.52	SACM1L
ENSG00000239388	-2.52	ASB14
ENSG00000182545	-2.52	RNASE10
ENSG00000222044	-2.52	
ENSG00000203469	-2.51	
ENSG00000188107	-2.51	EYS
ENSG00000151729	-2.51	SLC25A4
ENSG00000232344	-2.51	
ENSG00000287582	-2.51	
ENSG00000264247	-2.51	LINC00909
ENSG00000049167	-2.51	ERCC8
ENSG00000118690	-2.51	ARMC2
ENSG00000196418	-2.51	ZNF124
ENSG00000250299	-2.51	
ENSG00000257896	-2.51	
ENSG00000272791	-2.51	
ENSG00000274614	-2.51	
ENSG00000274808	-2.51	TBC1D3B
ENSG00000274987	-2.51	

ENSG00000279970	-2.51	
ENSG00000283992	-2.51	
ENSG00000287467	-2.51	
ENSG00000227512	-2.51	
ENSG00000146556	-2.51	WASH2P
ENSG00000270706	-2.51	
ENSG00000273162	-2.51	
ENSG00000120697	-2.51	ALG5
ENSG00000147799	-2.50	ARHGAP39
ENSG00000122783	-2.50	C7orf49
ENSG00000138134	-2.50	STAMBPL1
ENSG00000260302	-2.50	
ENSG00000100578	-2.50	KIAA0586
ENSG00000109674	-2.50	NEIL3
ENSG00000214514	-2.50	KRT42P
ENSG00000231789	-2.50	PIK3CD-AS2
ENSG00000243829	-2.50	
ENSG00000257302	-2.50	
ENSG00000267218	-2.50	
ENSG00000225331	-2.50	LINC01678
ENSG00000122550	-2.50	KLHL7
ENSG00000251279	-2.50	
ENSG00000263531	-2.50	
ENSG00000259948	-2.50	
ENSG00000152332	-2.49	UHMK1
ENSG00000138795	-2.49	LEF1
ENSG00000103888	-2.49	CEMIP
ENSG00000172845	-2.49	SP3
ENSG00000161649	-2.49	CD300LG
ENSG00000165169	-2.49	DYNLT3
ENSG00000236853	-2.49	
ENSG00000267219	-2.49	
ENSG00000270900	-2.49	
ENSG00000132436	-2.49	FIGNL1
ENSG00000257042	-2.49	
ENSG00000111319	-2.49	SCNN1A
ENSG00000236045	-2.49	
ENSG00000077514	-2.49	POLD3
ENSG00000136631	-2.49	VPS45
ENSG00000102738	-2.49	MRPS31
ENSG00000259081	-2.49	
ENSG00000270773	-2.49	

ENSG00000160207	-2.48	HSF2BP
ENSG00000275223	-2.48	
ENSG00000218416	-2.48	PP14571
ENSG00000170469	-2.48	SPATA24
ENSG00000131773	-2.48	KHDRBS3
ENSG00000173145	-2.48	NOC3L
ENSG00000204177	-2.48	BMS1P5
ENSG00000185247	-2.48	MAGEA11
ENSG00000160345	-2.48	C9orf116
ENSG00000261604	-2.48	
ENSG00000109572	-2.48	CLCN3
ENSG00000270184	-2.48	
ENSG00000185818	-2.48	NAT8L
ENSG00000181381	-2.47	DDX60L
ENSG00000153896	-2.47	ZNF599
ENSG00000251187	-2.47	
ENSG00000260973	-2.47	
ENSG00000019144	-2.47	PHLDB1
ENSG00000165304	-2.47	MELK
ENSG00000111245	-2.47	MYL2
ENSG00000271730	-2.47	
ENSG00000172336	-2.47	POP7
ENSG00000088726	-2.47	TMEM40
ENSG00000227695	-2.47	
ENSG00000241258	-2.46	CRCP
ENSG00000114423	-2.46	CBLB
ENSG00000176472	-2.46	ZNF575
ENSG00000128408	-2.46	RIBC2
ENSG00000267228	-2.46	
ENSG00000105607	-2.46	GCDH
ENSG00000008405	-2.46	CRY1
ENSG00000137992	-2.46	DBT
ENSG00000186862	-2.46	PDZD7
ENSG00000188211	-2.46	NCR3LG1
ENSG00000229839	-2.46	
ENSG00000230587	-2.46	
ENSG00000245384	-2.46	CXXC4-AS1
ENSG00000255008	-2.46	
ENSG00000255501	-2.46	CARD18
ENSG00000020922	-2.46	MRE11
ENSG00000181690	-2.46	PLAG1
ENSG00000153094	-2.46	BCL2L11

ENSG00000167395	-2.46	ZNF646
ENSG00000151835	-2.46	SACS
ENSG00000277639	-2.46	LOC105371267
ENSG00000117222	-2.46	RBBP5
ENSG00000176678	-2.46	FOXL1
ENSG00000256897	-2.46	
ENSG00000248254	-2.45	
ENSG00000172795	-2.45	DCP2
ENSG00000169981	-2.45	ZNF35
ENSG00000150787	-2.45	PTS
ENSG00000124107	-2.45	SLPI
ENSG00000138439	-2.45	FAM117B
ENSG00000198826	-2.45	ARHGAP11A
ENSG00000227345	-2.45	PARG
ENSG00000103642	-2.45	LACTB
ENSG00000005483	-2.44	KMT2E
ENSG00000101391	-2.44	CDK5RAP1
ENSG00000270039	-2.44	
ENSG00000260772	-2.44	
ENSG00000276663	-2.44	
ENSG00000242207	-2.44	
ENSG00000120341	-2.44	SEC16B
ENSG00000164823	-2.44	OSGIN2
ENSG00000203280	-2.44	LOC100128531
ENSG00000224465	-2.44	
ENSG00000010030	-2.44	ETV7
ENSG00000133392	-2.44	MYH11
ENSG00000154719	-2.44	MRPL39
ENSG00000238186	-2.43	
ENSG00000163132	-2.43	MSX1
ENSG00000106799	-2.43	TGFBR1
ENSG00000131668	-2.43	BARX1
ENSG00000155465	-2.43	SLC7A7
ENSG00000159374	-2.43	M1AP
ENSG00000188004	-2.43	SNHG28
ENSG00000227006	-2.43	
ENSG00000268649	-2.43	
ENSG00000168461	-2.43	RAB31
ENSG00000269068	-2.43	
ENSG00000106459	-2.43	NRF1
ENSG00000101680	-2.43	LAMA1
ENSG00000223725	-2.43	

ENSG00000103404	-2.43	USP31
ENSG00000254632	-2.43	
ENSG00000122435	-2.43	TRMT13
ENSG00000235098	-2.42	ANKRD65
ENSG00000162419	-2.42	GMEB1
ENSG00000134283	-2.42	PPHLN1
ENSG00000109066	-2.42	TMEM104
ENSG00000171566	-2.42	PLRG1
ENSG00000121152	-2.42	NCAPH
ENSG00000146094	-2.42	DOK3
ENSG00000146267	-2.42	FAXC
ENSG00000174928	-2.42	C3orf33
ENSG00000185811	-2.42	IKZF1
ENSG00000228364	-2.42	
ENSG00000258323	-2.42	
ENSG00000272211	-2.42	
ENSG00000254428	-2.42	
ENSG00000228816	-2.42	
ENSG00000234630	-2.42	
ENSG00000104093	-2.42	DMXL2
ENSG00000255302	-2.42	EID1
ENSG00000081923	-2.41	ATP8B1
ENSG00000213420	-2.41	GPC2
ENSG00000023041	-2.41	ZDHHC6
ENSG00000158458	-2.41	NRG2
ENSG00000174529	-2.41	TMEM81
ENSG00000229228	-2.41	LINC00582
ENSG00000250909	-2.41	
ENSG00000170185	-2.41	USP38
ENSG00000135451	-2.41	TROAP
ENSG00000169139	-2.41	UBE2V2
ENSG00000167528	-2.41	ZNF641
ENSG00000120533	-2.41	ENY2
ENSG00000137502	-2.41	RAB30
ENSG00000138658	-2.40	ZGRF1
ENSG00000107938	-2.40	EDRF1
ENSG00000007516	-2.40	BAIAP3
ENSG00000256690	-2.40	LOC105369332
ENSG00000151657	-2.40	KIN
ENSG00000197180	-2.40	CH17-340M24.3
ENSG00000257764	-2.40	
ENSG00000260834	-2.40	

ENSG00000288596	-2.40	
ENSG00000135960	-2.40	EDAR
ENSG00000051341	-2.40	POLQ
ENSG00000176105	-2.40	YES1
ENSG00000283268	-2.40	
ENSG00000270871	-2.40	
ENSG00000091262	-2.40	ABCC6
ENSG00000225726	-2.40	
ENSG00000112238	-2.40	PRDM13
ENSG00000174953	-2.40	DHX36
ENSG00000167685	-2.39	ZNF444
ENSG00000158286	-2.39	RNF207
ENSG00000164048	-2.39	ZNF589
ENSG00000164347	-2.39	GFM2
ENSG00000180818	-2.39	HOXC10
ENSG00000197444	-2.39	OGDHL
ENSG00000225518	-2.39	
ENSG00000100625	-2.39	SIX4
ENSG00000198417	-2.38	MT1F
ENSG00000197601	-2.38	FAR1
ENSG00000136560	-2.38	TANK
ENSG00000152193	-2.38	RNF219
ENSG00000270882	-2.38	HIST2H4A
ENSG00000143850	-2.38	PLEKHA6
ENSG00000124915	-2.38	DKFZP434K028
ENSG00000170442	-2.38	KRT86
ENSG00000232677	-2.38	LINC00665
ENSG00000253197	-2.38	
ENSG00000283352	-2.38	LINC00680-GUSBP4
ENSG00000284128	-2.38	
ENSG00000145087	-2.38	STXBP5L
ENSG00000134020	-2.38	PEBP4
ENSG00000129048	-2.38	ACKR4
ENSG00000265443	-2.38	
ENSG00000251095	-2.38	
ENSG00000115194	-2.38	SLC30A3
ENSG00000135297	-2.38	MTO1
ENSG00000175104	-2.38	TRAF6
ENSG00000232284	-2.37	GNG12-AS1
ENSG00000026103	-2.37	FAS
ENSG00000166352	-2.37	C11orf74
ENSG00000245694	-2.37	LOC101927480

ENSG00000154839	-2.37	SKA1
ENSG00000197603	-2.37	C5orf42
ENSG00000235774	-2.37	
ENSG00000239207	-2.37	
ENSG00000272234	-2.37	
ENSG00000234147	-2.37	
ENSG00000253445	-2.37	
ENSG00000152242	-2.37	C18orf25
ENSG00000172244	-2.36	C5orf34
ENSG00000198399	-2.36	ITSN2
ENSG00000246982	-2.36	
ENSG00000224195	-2.36	
ENSG00000261188	-2.36	
ENSG00000277135	-2.36	
ENSG00000286042	-2.36	
ENSG00000131591	-2.36	C1orf159
ENSG00000154059	-2.36	IMPACT
ENSG00000181143	-2.36	MUC16
ENSG00000111790	-2.36	FGFR1OP2
ENSG00000121410	-2.36	A1BG
ENSG00000015133	-2.36	CCDC88C
ENSG00000101901	-2.35	ALG13
ENSG00000163909	-2.35	HEYL
ENSG00000257553	-2.35	
ENSG00000021300	-2.35	PLEKHB1
ENSG00000077935	-2.35	SMC1B
ENSG00000109099	-2.35	PMP22
ENSG00000128692	-2.35	
ENSG00000256667	-2.35	
ENSG00000274215	-2.35	
ENSG00000164649	-2.35	CDCA7L
ENSG00000282885	-2.35	
ENSG00000070808	-2.35	CAMK2A
ENSG00000233436	-2.35	BTBD18
ENSG00000011405	-2.35	PIK3C2A
ENSG00000082512	-2.35	TRAF5
ENSG00000141971	-2.34	MVB12A
ENSG00000062194	-2.34	GPBP1
ENSG00000169914	-2.34	OTUD3
ENSG00000212978	-2.34	LOC339803
ENSG00000138385	-2.34	SSB
ENSG00000154727	-2.34	GABPA

ENSG00000259187	-2.34	
ENSG00000157036	-2.34	EXOG
ENSG00000188257	-2.34	PLA2G2A
ENSG00000237638	-2.34	
ENSG00000267857	-2.34	
ENSG00000284642	-2.34	
ENSG00000286548	-2.34	
ENSG00000171320	-2.34	ESCO2
ENSG00000178307	-2.34	TMEM11
ENSG00000232220	-2.34	
ENSG00000152223	-2.34	EPG5
ENSG00000118217	-2.34	ATF6
ENSG00000235961	-2.34	PNMA6A
ENSG00000119682	-2.33	AREL1
ENSG00000164022	-2.33	AIMP1
ENSG00000272645	-2.33	
ENSG00000134248	-2.33	LAMTOR5
ENSG00000236751	-2.33	LINC01186
ENSG00000254338	-2.33	MAFA-AS1
ENSG00000136492	-2.33	BRIP1
ENSG00000170161	-2.33	LOC728673
ENSG00000256271	-2.33	CACNA1C-AS2
ENSG00000024526	-2.33	DEPDC1
ENSG00000276116	-2.33	
ENSG00000170260	-2.33	ZNF212
ENSG00000118194	-2.32	TNNT2
ENSG00000251503	-2.32	CENPS-CORT
ENSG00000151849	-2.32	CENPJ
ENSG00000257957	-2.32	
ENSG00000171094	-2.32	ALK
ENSG00000189195	-2.32	
ENSG00000215097	-2.32	
ENSG00000268751	-2.32	
ENSG00000271937	-2.32	
ENSG00000096070	-2.32	BRPF3
ENSG00000166348	-2.32	USP54
ENSG00000267709	-2.32	LOC101928844
ENSG00000172840	-2.32	PDP2
ENSG00000266578	-2.32	
ENSG00000075651	-2.32	PLD1
ENSG00000115944	-2.32	COX7A2L
ENSG00000126261	-2.32	UBA2

ENSG00000132879	-2.32	FBXO44
ENSG00000170312	-2.32	CDK1
ENSG00000182324	-2.31	KCNJ14
ENSG00000143630	-2.31	HCN3
ENSG00000048649	-2.31	RSF1
ENSG00000135002	-2.31	RFK
ENSG00000185088	-2.31	RPS27L
ENSG00000125285	-2.31	SOX21
ENSG00000172543	-2.31	CTSW
ENSG00000178229	-2.31	ZNF543
ENSG00000243896	-2.31	OR2A7
ENSG00000267227	-2.31	
ENSG00000286507	-2.31	
ENSG00000089006	-2.31	SNX5
ENSG00000273188	-2.31	
ENSG00000185686	-2.31	PRAME
ENSG00000183386	-2.30	FHL3
ENSG00000129003	-2.30	VPS13C
ENSG00000228782	-2.30	
ENSG00000166813	-2.30	KIF7
ENSG00000168137	-2.30	SETD5
ENSG00000254495	-2.30	
ENSG00000230797	-2.30	YY2
ENSG00000234751	-2.30	
ENSG00000279809	-2.30	
ENSG00000213780	-2.30	GTF2H4
ENSG00000135637	-2.30	CCDC142
ENSG00000010219	-2.30	DYRK4
ENSG00000262903	-2.30	
ENSG00000103995	-2.29	CEP152
ENSG00000176732	-2.29	PFN4
ENSG00000180914	-2.29	OXTR
ENSG00000271711	-2.29	
ENSG00000272968	-2.29	RBAK-RBAKDN
ENSG00000273284	-2.29	
ENSG00000197712	-2.29	FAM114A1
ENSG00000147324	-2.29	MFHAS1
ENSG00000026025	-2.29	VIM
ENSG00000111832	-2.29	RWDD1
ENSG00000184368	-2.29	MAP7D2
ENSG00000248223	-2.29	
ENSG00000167487	-2.29	KLHL26

ENSG00000176208	-2.29	ATAD5	
ENSG00000142945	-2.29	KIF2C	
ENSG00000171914	-2.29	TLN2	
ENSG00000139531	-2.29	SUOX	
ENSG00000169499	-2.29	PLEKHA2	
ENSG00000154920	-2.29	EME1	
ENSG00000131069	-2.28	ACSS2	
ENSG00000236908	-2.28		
ENSG00000182224	-2.28	CYB5D1	
ENSG00000140104	-2.28	C14orf79	
ENSG00000185668	-2.28	POU3F1	
ENSG00000205639	-2.28	MFSD2B	
ENSG00000279583	-2.28		
ENSG00000134744	-2.28	ZCCHC11	
ENSG00000104549	-2.28	SQLE	
ENSG00000227855	-2.28	DPY19L2P3	
ENSG00000096717	-2.27	SIRT1	
ENSG00000152977	-2.27	ZIC1	
ENSG00000151239	-2.27	TWF1	
ENSG00000254087	-2.27	LYN	
ENSG00000267645	-2.27		
ENSG00000176349	-2.27		
ENSG00000235217	-2.27	TSPY26P	
ENSG00000186522	-2.27		10-Sep
ENSG00000070081	-2.27	NUCB2	
ENSG00000080819	-2.27	CPOX	
ENSG00000136444	-2.27	RSAD1	
ENSG00000114738	-2.27	MAPKAPK3	
ENSG00000250397	-2.27		
ENSG00000163138	-2.27	PACRGL	
ENSG00000196792	-2.26	STRN3	
ENSG00000182240	-2.26	BACE2	
ENSG00000100764	-2.26	PSMC1	
ENSG00000140396	-2.26	NCOA2	
ENSG00000178226	-2.26	PRSS36	
ENSG00000070886	-2.26	EPHA8	
ENSG00000184185	-2.26	KCNJ12	
ENSG00000215283	-2.26		
ENSG00000236137	-2.26	LOC101929099	
ENSG00000257315	-2.26	ZBED6	
ENSG00000113811	-2.26	SELENOK	
ENSG00000105197	-2.26	TIMM50	

ENSG00000232821	-2.26	
ENSG00000253930	-2.26	
ENSG00000069329	-2.26	VPS35
ENSG00000121940	-2.26	CLCC1
ENSG00000164438	-2.26	TLX3
ENSG00000075218	-2.25	GTSE1
ENSG00000085231	-2.25	AK6
ENSG00000137601	-2.25	NEK1
ENSG00000136982	-2.25	DSCC1
ENSG00000197563	-2.25	PIGN
ENSG00000077458	-2.25	FAM76B
ENSG00000229029	-2.25	
ENSG00000255847	-2.25	
ENSG00000111490	-2.25	TBC1D30
ENSG00000164885	-2.25	CDK5
ENSG00000164038	-2.25	SLC9B2
ENSG00000135541	-2.25	AHI1
ENSG00000108010	-2.25	GLRX3
ENSG00000259295	-2.25	
ENSG00000095739	-2.25	BAMBI
ENSG00000116353	-2.25	MECR
ENSG00000239467	-2.25	
ENSG00000158769	-2.25	F11R
ENSG00000141279	-2.24	NPEPPS
ENSG00000090612	-2.24	ZNF268
ENSG00000249207	-2.24	
ENSG00000204267	-2.24	TAP2
ENSG00000125319	-2.24	C17orf53
ENSG00000141040	-2.24	ZNF287
ENSG00000173917	-2.24	HOXB2
ENSG00000206028	-2.24	
ENSG00000248896	-2.24	
ENSG00000254340	-2.24	
ENSG00000267705	-2.24	
ENSG00000196118	-2.24	CCDC189
ENSG00000132004	-2.24	FBXW9
ENSG00000279345	-2.24	
ENSG00000163032	-2.24	VSNL1
ENSG00000063438	-2.24	AHRR
ENSG00000171970	-2.23	ZNF57
ENSG00000075303	-2.23	SLC25A40
ENSG00000166575	-2.23	TMEM135

ENSG00000017260	-2.23	ATP2C1
ENSG00000111885	-2.23	MAN1A1
ENSG00000121621	-2.23	KIF18A
ENSG00000213176	-2.23	
ENSG00000260447	-2.23	
ENSG00000273036	-2.23	FAM95C
ENSG00000123080	-2.23	CDKN2C
ENSG00000136436	-2.23	CALCOCO2
ENSG00000185361	-2.23	TNFAIP8L1
ENSG00000215252	-2.23	GOLGA8B
ENSG00000081087	-2.23	OSTM1
ENSG00000166037	-2.23	CEP57
ENSG00000036549	-2.23	ZZZ3
ENSG00000125885	-2.23	MCM8
ENSG00000069956	-2.23	MAPK6
ENSG00000133704	-2.23	IPO8
ENSG00000149262	-2.22	INTS4
ENSG00000153046	-2.22	CDYL
ENSG00000272473	-2.22	
ENSG00000170234	-2.22	PWWP2A
ENSG00000127415	-2.22	IDUA
ENSG00000179988	-2.22	PSTK
ENSG00000068079	-2.22	IFI35
ENSG00000273047	-2.22	
ENSG00000185652	-2.22	NTF3
ENSG00000251139	-2.22	
ENSG00000256043	-2.22	CTSO
ENSG00000279532	-2.22	
ENSG00000180447	-2.22	GAS1
ENSG00000270820	-2.22	
ENSG00000180902	-2.21	D2HGDH
ENSG00000151348	-2.21	EXT2
ENSG00000198924	-2.21	DCLRE1A
ENSG00000272970	-2.21	
ENSG00000279203	-2.21	
ENSG00000106144	-2.21	CASP2
ENSG00000126368	-2.21	NR1D1
ENSG00000114841	-2.21	DNAH1
ENSG00000169174	-2.21	PCSK9
ENSG00000070367	-2.21	EXOC5
ENSG00000138231	-2.21	DBR1
ENSG00000030419	-2.21	IKZF2

ENSG00000101849	-2.20	TBL1X
ENSG00000159579	-2.20	RSPRY1
ENSG00000168701	-2.20	TMEM208
ENSG00000269982	-2.20	
ENSG00000267001	-2.20	
ENSG00000160867	-2.20	FGFR4
ENSG00000106278	-2.20	PTPRZ1
ENSG00000182484	-2.20	
ENSG00000105865	-2.20	DUS4L
ENSG00000138430	-2.20	OLA1
ENSG00000102048	-2.20	ASB9
ENSG00000170465	-2.20	KRT6C
ENSG00000229127	-2.20	
ENSG00000270182	-2.20	
ENSG00000131323	-2.20	TRAF3
ENSG00000132912	-2.20	DCTN4
ENSG00000176834	-2.20	VSIG10
ENSG00000144407	-2.20	PTH2R
ENSG00000134375	-2.20	TIMM17A
ENSG00000162664	-2.20	ZNF326
ENSG00000143207	-2.20	RFWD2
ENSG00000136044	-2.19	APPL2
ENSG00000184445	-2.19	KNTC1
ENSG00000136059	-2.19	VILL
ENSG00000174669	-2.19	SLC29A2
ENSG00000009413	-2.19	REV3L
ENSG00000186918	-2.19	ZNF395
ENSG00000151287	-2.19	TEX30
ENSG00000158234	-2.19	FAIM
ENSG00000203867	-2.19	RBM20
ENSG00000228352	-2.19	
ENSG00000229931	-2.19	
ENSG00000247934	-2.19	
ENSG00000267736	-2.19	
ENSG00000286288	-2.19	
ENSG00000197498	-2.19	RPF2
ENSG00000133872	-2.19	SARAF
ENSG00000248643	-2.19	RBM14-RBM4
ENSG00000129484	-2.19	PARP2
ENSG00000175354	-2.19	PTPN2
ENSG00000134809	-2.18	TIMM10
ENSG00000180098	-2.18	TRNAU1AP

ENSG00000104321	-2.18	TRPA1
ENSG00000081665	-2.18	ZNF506
ENSG00000135315	-2.18	CEP162
ENSG00000159915	-2.18	ZNF233
ENSG00000072182	-2.18	ASIC4
ENSG00000260328	-2.18	
ENSG00000268603	-2.18	
ENSG00000279778	-2.18	
ENSG00000136943	-2.18	CTSV
ENSG00000172315	-2.18	TP53RK
ENSG00000188610	-2.18	FAM72B
ENSG00000123094	-2.18	RASSF8
ENSG00000214765	-2.17	SEPT7P2
ENSG00000175745	-2.17	NR2F1
ENSG00000136574	-2.17	GATA4
ENSG00000273841	-2.17	TAF9
ENSG00000156858	-2.17	PRR14
ENSG00000104833	-2.17	TUBB4A
ENSG00000107951	-2.17	MTPAP
ENSG00000187790	-2.17	FANCM
ENSG00000231566	-2.17	LOC401585
ENSG00000262583	-2.17	
ENSG00000270986	-2.17	
ENSG00000286124	-2.17	
ENSG00000286435	-2.17	
ENSG00000267064	-2.17	UXT-AS1
ENSG00000185532	-2.17	PRKG1
ENSG00000168883	-2.17	USP39
ENSG00000076067	-2.17	RBMS2
ENSG00000234742	-2.17	
ENSG00000224189	-2.17	HAGLR
ENSG00000116095	-2.16	PLEKHA3
ENSG00000185591	-2.16	SP1
ENSG00000118518	-2.16	RNF146
ENSG00000204616	-2.16	TRIM31
ENSG00000110871	-2.16	COQ5
ENSG00000270179	-2.16	
ENSG00000279253	-2.16	
ENSG00000286159	-2.16	
ENSG00000086102	-2.16	NFX1
ENSG00000260669	-2.16	
ENSG00000119431	-2.16	HDHD3

ENSG00000154310	-2.15	TNIK
ENSG00000264343	-2.15	NOTCH2NL
ENSG00000229413	-2.15	
ENSG00000131096	-2.15	PYY
ENSG00000196593	-2.15	
ENSG00000214946	-2.15	TBC1D26
ENSG00000245281	-2.15	LOC101929066
ENSG00000104783	-2.15	KCNN4
ENSG00000253144	-2.15	
ENSG00000143970	-2.15	ASXL2
ENSG00000250251	-2.15	
ENSG00000263711	-2.15	LOC400655
ENSG00000224505	-2.15	
ENSG00000103494	-2.14	RPGRIP1L
ENSG00000236830	-2.14	CBR3-AS1
ENSG00000101266	-2.14	CSNK2A1
ENSG00000137752	-2.14	CASP1
ENSG00000003393	-2.14	ALS2
ENSG00000233860	-2.14	
ENSG00000198162	-2.14	MAN1A2
ENSG00000238098	-2.14	ABCA17P
ENSG00000076258	-2.14	FMO4
ENSG00000108239	-2.14	TBC1D12
ENSG00000135917	-2.14	SLC19A3
ENSG00000178385	-2.14	PLEKHM3
ENSG00000225165	-2.14	
ENSG00000225442	-2.14	
ENSG00000227304	-2.14	
ENSG00000234235	-2.14	
ENSG00000260186	-2.14	
ENSG00000278486	-2.14	
ENSG00000025434	-2.14	NR1H3
ENSG00000164920	-2.14	OSR2
ENSG00000163001	-2.14	CFAP36
ENSG00000109625	-2.14	CPZ
ENSG00000087510	-2.14	TFAP2C
ENSG00000175305	-2.14	CCNE2
ENSG00000072682	-2.14	P4HA2
ENSG00000246596	-2.14	
ENSG00000225663	-2.14	MCRIP1
ENSG00000109171	-2.13	SLAIN2
ENSG00000287600	-2.13	

ENSG00000242948	-2.13	
ENSG00000274828	-2.13	
ENSG00000172638	-2.13	EFEMP2
ENSG00000016864	-2.13	GLT8D1
ENSG00000100908	-2.13	EMC9
ENSG00000168263	-2.13	KCNV2
ENSG00000165525	-2.13	NEMF
ENSG00000177200	-2.13	CHD9
ENSG00000147118	-2.13	ZNF182
ENSG00000180764	-2.13	PIPSL
ENSG00000213066	-2.13	FGFR1OP
ENSG00000249884	-2.13	RNF103-CHMP3
ENSG00000286257	-2.13	
ENSG00000285077	-2.13	
ENSG00000113430	-2.13	IRX4
ENSG00000126562	-2.13	WNK4
ENSG00000119772	-2.13	DNMT3A
ENSG00000267882	-2.13	LOC100131496
ENSG00000247679	-2.13	
ENSG00000186047	-2.13	DLEU7
ENSG00000258761	-2.12	
ENSG00000271119	-2.12	
ENSG00000172927	-2.12	MYEOV
ENSG00000099953	-2.12	MMP11
ENSG00000126456	-2.12	IRF3
ENSG00000164742	-2.12	ADCY1
ENSG00000184619	-2.12	KRBA2
ENSG00000214439	-2.12	
ENSG00000278291	-2.12	
ENSG00000284188	-2.12	
ENSG00000129480	-2.12	DTD2
ENSG00000130202	-2.12	NECTIN2
ENSG00000114999	-2.12	TTL
ENSG00000105185	-2.12	PDCD5
ENSG00000143353	-2.12	LYPLAL1
ENSG00000266925	-2.12	
ENSG00000196678	-2.12	ERI2
ENSG00000197894	-2.12	ADH5
ENSG00000141098	-2.12	GFOD2
ENSG00000171606	-2.11	ZNF274
ENSG00000106344	-2.11	RBM28
ENSG00000226419	-2.11	SLC16A1-AS1

ENSG00000229205	-2.11	LINC00200
ENSG00000159459	-2.11	UBR1
ENSG00000107021	-2.11	TBC1D13
ENSG00000162493	-2.11	PDPN
ENSG00000087206	-2.11	UIMC1
ENSG00000101335	-2.11	MYL9
ENSG00000173451	-2.11	THAP2
ENSG00000175536	-2.11	LIPT2
ENSG00000223443	-2.11	USP17L2
ENSG00000224928	-2.11	
ENSG00000234350	-2.11	
ENSG00000274460	-2.11	
ENSG00000285162	-2.11	
ENSG00000112877	-2.11	CEP72
ENSG00000147475	-2.11	ERLIN2
ENSG00000286670	-2.11	
ENSG00000055332	-2.11	EIF2AK2
ENSG00000171311	-2.10	EXOSC1
ENSG00000115421	-2.10	PAPOLG
ENSG00000120693	-2.10	SMAD9
ENSG00000106686	-2.10	SPATA6L
ENSG00000136040	-2.10	PLXNC1
ENSG00000279386	-2.10	
ENSG00000163923	-2.10	RPL39L
ENSG00000176714	-2.10	CCDC121
ENSG00000221930	-2.10	
ENSG00000226360	-2.10	
ENSG00000259209	-2.10	
ENSG00000286544	-2.10	
ENSG00000117226	-2.10	GBP3
ENSG00000170322	-2.10	NFRKB
ENSG00000122068	-2.10	FYTTD1
ENSG00000011426	-2.10	ANLN
ENSG00000086475	-2.10	SEPHS1
ENSG00000255467	-2.10	
ENSG00000180096	-2.10	SEPT1
ENSG00000152217	-2.10	SETBP1
ENSG00000124120	-2.10	TTPAL
ENSG00000071282	-2.09	LMCD1
ENSG00000224078	-2.09	
ENSG00000176853	-2.09	FAM91A1
ENSG00000185201	-2.09	IFITM2

ENSG00000137563	-2.09	GGH
ENSG00000166278	-2.09	C2
ENSG00000279300	-2.09	
ENSG00000278993	-2.09	
ENSG00000189377	-2.09	CXCL17
ENSG00000273654	-2.09	
ENSG00000198785	-2.09	GRIN3A
ENSG00000167851	-2.09	CD300A
ENSG00000170144	-2.09	HNRNPA3
ENSG00000272849	-2.09	
ENSG00000070371	-2.09	CLTCL1
ENSG00000162639	-2.09	HENMT1
ENSG00000123739	-2.08	PLA2G12A
ENSG00000164251	-2.08	F2RL1
ENSG00000129465	-2.08	RIPK3
ENSG00000069399	-2.08	BCL3
ENSG00000173295	-2.08	FAM86B3P
ENSG00000204536	-2.08	CCHCR1
ENSG00000066135	-2.08	KDM4A
ENSG00000047056	-2.08	WDR37
ENSG00000232230	-2.08	
ENSG00000166123	-2.08	GPT2
ENSG00000198920	-2.08	KIAA0753
ENSG00000196591	-2.08	HDAC2
ENSG00000126218	-2.08	F10
ENSG00000196684	-2.08	HSH2D
ENSG00000228084	-2.08	
ENSG00000237922	-2.08	
ENSG00000238039	-2.08	
ENSG00000246366	-2.08	LACTB2-AS1
ENSG00000253671	-2.08	
ENSG00000256262	-2.08	
ENSG00000286219	-2.08	
ENSG00000134759	-2.08	ELP2
ENSG00000047634	-2.07	SCML1
ENSG00000260012	-2.07	
ENSG00000171456	-2.07	ASXL1
ENSG00000104361	-2.07	NIPAL2
ENSG00000158201	-2.07	ABHD3
ENSG00000267270	-2.07	PARD6G-AS1
ENSG00000112144	-2.07	ICK
ENSG00000267396	-2.07	

ENSG00000120802	-2.07	TMPO
ENSG00000118242	-2.07	MREG
ENSG00000107036	-2.07	RIC1
ENSG00000188266	-2.07	HYKK
ENSG00000008517	-2.07	IL32
ENSG00000213965	-2.07	NUDT19
ENSG00000145103	-2.07	ILDR1
ENSG00000076716	-2.07	GPC4
ENSG00000204622	-2.07	HLA-J
ENSG00000224184	-2.07	MIR3681HG
ENSG00000233427	-2.07	
ENSG00000245059	-2.07	
ENSG00000214145	-2.07	LINC00887
ENSG00000258959	-2.07	
ENSG00000272398	-2.07	CD24
ENSG00000140450	-2.07	ARRDC4
ENSG00000188958	-2.07	UTS2B
ENSG00000112812	-2.07	PRSS16
ENSG00000164611	-2.07	PTTG1
ENSG00000027869	-2.07	SH2D2A
ENSG00000070540	-2.07	WIPI1
ENSG00000112234	-2.06	FBXL4
ENSG00000253908	-2.06	
ENSG00000186625	-2.06	KATNA1
ENSG00000261602	-2.06	
ENSG00000070061	-2.06	ELP1
ENSG00000254166	-2.06	
ENSG00000168118	-2.06	RAB4A
ENSG00000113231	-2.06	PDE8B
ENSG00000221817	-2.06	PPP3CB-AS1
ENSG00000164542	-2.06	KIAA0895
ENSG00000135378	-2.06	PRRG4
ENSG00000132321	-2.06	IQCA1
ENSG00000132518	-2.06	GUCY2D
ENSG00000224728	-2.06	
ENSG00000224973	-2.06	LARGE-AS1
ENSG00000276007	-2.06	
ENSG00000149823	-2.06	VPS51
ENSG00000158373	-2.06	HIST1H2BD
ENSG00000273456	-2.06	
ENSG00000251867	-2.06	
ENSG00000234694	-2.06	LOC105378687

ENSG00000278964	-2.06	
ENSG00000169951	-2.06	ZNF764
ENSG00000101224	-2.06	CDC25B
ENSG00000186197	-2.06	EDARADD
ENSG00000285287	-2.06	
ENSG00000143179	-2.05	UCK2
ENSG00000143543	-2.05	JTB
ENSG00000143079	-2.05	CTTNBP2NL
ENSG00000264522	-2.05	OTUD7B
ENSG00000169019	-2.05	COMMD8
ENSG00000285565	-2.05	
ENSG00000163202	-2.05	LCE3D
ENSG00000272386	-2.05	
ENSG00000170779	-2.05	CDCA4
ENSG00000109220	-2.05	CHIC2
ENSG00000148481	-2.05	MINDY3
ENSG00000169894	-2.05	MUC3A
ENSG00000183742	-2.05	MACC1
ENSG00000225969	-2.05	ABHD11-AS1
ENSG00000226200	-2.05	
ENSG00000251246	-2.05	
ENSG00000274272	-2.05	
ENSG00000104472	-2.05	CHRAC1
ENSG00000163781	-2.05	TOPBP1
ENSG00000135525	-2.05	MAP7
ENSG00000260899	-2.05	
ENSG00000182963	-2.05	GJC1
ENSG00000198431	-2.05	TXNRD1
ENSG00000155099	-2.04	TMEM55A
ENSG00000145391	-2.04	SETD7
ENSG00000109814	-2.04	UGDH
ENSG00000075539	-2.04	FRYL
ENSG00000102572	-2.04	STK24
ENSG00000243509	-2.04	TNFRSF6B
ENSG00000064651	-2.04	SLC12A2
ENSG00000139946	-2.04	PELI2
ENSG00000259341	-2.04	
ENSG00000102710	-2.04	SUPT20H
ENSG00000167653	-2.04	PSCA
ENSG00000150627	-2.04	WDR17
ENSG00000135372	-2.04	NAT10
ENSG00000162231	-2.04	NXF1

ENSG00000277383	-2.04	
ENSG00000090971	-2.04	NAT14
ENSG00000109270	-2.04	LAMTOR3
ENSG00000286070	-2.04	
ENSG00000213160	-2.04	PHOSPHO2-KLHL23
ENSG00000181929	-2.04	PRKAG1
ENSG00000155275	-2.04	TRMT44
ENSG00000109107	-2.04	ALDOC
ENSG00000171476	-2.04	HOPX
ENSG00000163297	-2.04	ANTXR2
ENSG00000184497	-2.04	TMEM255B
ENSG00000185418	-2.03	TARSL2
ENSG00000262848	-2.03	
ENSG00000128951	-2.03	DUT
ENSG00000013725	-2.03	CD6
ENSG00000145365	-2.03	TIFA
ENSG00000240038	-2.03	AMY2B
ENSG00000186364	-2.03	NUDT17
ENSG00000131747	-2.03	TOP2A
ENSG00000162433	-2.03	AK4
ENSG00000170448	-2.03	NFXL1
ENSG00000079150	-2.03	FKBP7
ENSG00000143669	-2.03	LYST
ENSG00000169071	-2.03	ROR2
ENSG00000205403	-2.03	CFI
ENSG00000223804	-2.03	
ENSG00000227725	-2.03	
ENSG00000232710	-2.03	
ENSG00000233191	-2.03	
ENSG00000275557	-2.03	
ENSG00000255468	-2.03	LOC102724064
ENSG00000122678	-2.03	POLM
ENSG00000196975	-2.03	ANXA4
ENSG00000261526	-2.03	
ENSG00000020129	-2.03	NCDN
ENSG00000184708	-2.03	EIF4ENIF1
ENSG00000102178	-2.03	UBL4A
ENSG00000165732	-2.03	DDX21
ENSG00000197714	-2.03	ZNF460
ENSG00000164011	-2.03	ZNF691
ENSG00000143942	-2.02	CHAC2
ENSG00000169246	-2.02	

ENSG00000168994	-2.02	PXDC1
ENSG00000168566	-2.02	SNRNP48
ENSG00000070018	-2.02	LRP6
ENSG00000108448	-2.02	TRIM16L
ENSG00000140694	-2.02	PARN
ENSG00000288604	-2.02	
ENSG00000164849	-2.02	GPR146
ENSG00000055208	-2.02	
ENSG00000112739	-2.02	PRPF4B
ENSG00000286223	-2.02	
ENSG00000103426	-2.02	CORO7-PAM16
ENSG00000168228	-2.02	ZCCHC4
ENSG00000231889	-2.02	TRAF3IP2-AS1
ENSG00000240698	-2.02	
ENSG00000251203	-2.02	
ENSG00000287644	-2.02	
ENSG00000105516	-2.02	DBP
ENSG00000271780	-2.02	
ENSG00000184584	-2.02	TMEM173
ENSG00000232439	-2.02	
ENSG00000129657	-2.02	SEC14L1
ENSG00000175216	-2.02	CKAP5
ENSG00000133731	-2.02	IMPA1
ENSG00000243701	-2.01	DUBR
ENSG00000165995	-2.01	CACNB2
ENSG00000179168	-2.01	GGN
ENSG00000198783	-2.01	ZNF830
ENSG00000116922	-2.01	C1orf109
ENSG00000224747	-2.01	
ENSG00000225872	-2.01	LINC01529
ENSG00000260774	-2.01	
ENSG00000133597	-2.01	ADCK2
ENSG00000143376	-2.01	SNX27
ENSG00000255062	-2.01	
ENSG00000099954	-2.01	CECR2
ENSG00000180834	-2.01	MAP6D1
ENSG00000067191	-2.01	CACNB1
ENSG00000133398	-2.01	MED10
ENSG00000163607	-2.01	GTPBP8
ENSG00000006576	-2.01	PHTF2
ENSG00000177225	-2.01	GATD1
ENSG00000243279	-2.01	PRAF2

ENSG00000254680	-2.00	
ENSG00000189077	-2.00	TMEM120A
ENSG00000230325	-2.00	
ENSG00000119514	-2.00	GALNT12
ENSG00000125962	-2.00	ARMCX5
ENSG00000149742	-2.00	SLC22A9
ENSG00000196834	-2.00	POTEI
ENSG00000228697	-2.00	LOC101928565
ENSG00000228804	-2.00	LOC100131635
ENSG00000237225	-2.00	
ENSG00000271384	-2.00	
ENSG00000284678	-2.00	
ENSG00000010539	2.00	ZNF200
ENSG00000140265	2.00	ZSCAN29
ENSG00000226318	2.00	
ENSG00000249064	2.00	
ENSG00000253943	2.00	
ENSG00000254470	2.00	AP5B1
ENSG00000152061	2.00	RABGAP1L
ENSG00000258942	2.00	
ENSG00000164182	2.00	NDUFAF2
ENSG00000138942	2.01	RNF185
ENSG00000229757	2.01	
ENSG00000091428	2.01	RAPGEF4
ENSG00000186529	2.01	CYP4F3
ENSG00000106615	2.01	RHEB
ENSG00000172803	2.01	SNX32
ENSG00000136518	2.01	ACTL6A
ENSG00000101846	2.01	STS
ENSG00000168116	2.01	KIAA1586
ENSG00000168876	2.01	ANKRD49
ENSG00000214659	2.01	
ENSG00000237807	2.01	
ENSG00000261424	2.01	
ENSG00000270800	2.01	RPS10-NUDT3
ENSG00000281756	2.01	C2-AS1
ENSG00000287970	2.01	
ENSG00000179627	2.01	ZBTB42
ENSG00000123505	2.01	AMD1
ENSG00000279571	2.01	
ENSG00000279602	2.01	
ENSG00000197696	2.01	NMB

ENSG00000268785	2.01	
ENSG00000168389	2.01	MFSD2A
ENSG00000165915	2.01	SLC39A13
ENSG00000154803	2.01	FLCN
ENSG00000247796	2.01	LOC257396
ENSG00000126821	2.01	SGPP1
ENSG00000120910	2.01	PPP3CC
ENSG00000259448	2.01	
ENSG00000048140	2.02	TSPAN17
ENSG00000122674	2.02	CCZ1
ENSG00000050405	2.02	LIMA1
ENSG00000143363	2.02	PRUNE1
ENSG00000167604	2.02	NFKBID
ENSG00000186162	2.02	CIDECP
ENSG00000144306	2.02	SCRN3
ENSG00000142609	2.02	CFAP74
ENSG00000269481	2.02	
ENSG00000179335	2.02	CLK3
ENSG00000134398	2.02	ERN2
ENSG00000184774	2.02	MGAT4EP
ENSG00000223561	2.02	LOC646588
ENSG00000231509	2.02	
ENSG00000255639	2.02	
ENSG00000237476	2.02	LINC01637
ENSG00000267855	2.02	
ENSG00000111269	2.02	CREBL2
ENSG00000165646	2.02	SLC18A2
ENSG00000171798	2.02	KNDC1
ENSG00000140332	2.02	TLE3
ENSG00000254369	2.02	HOXA-AS3
ENSG00000082014	2.02	SMARCD3
ENSG00000230438	2.03	
ENSG00000272183	2.03	
ENSG00000151135	2.03	TMEM263
ENSG00000170242	2.03	USP47
ENSG00000115841	2.03	RMDN2
ENSG00000237989	2.03	LINC01679
ENSG00000279803	2.03	
ENSG00000226328	2.03	
ENSG00000136319	2.03	TTC5
ENSG00000278769	2.03	
ENSG00000227802	2.03	

ENSG00000197386	2.03	HTT
ENSG00000169131	2.03	ZNF354A
ENSG00000104320	2.03	NBN
ENSG00000167578	2.04	
ENSG00000181322	2.04	NME9
ENSG00000166716	2.04	ZNF592
ENSG00000135048	2.04	TMEM2
ENSG00000288656	2.04	
ENSG00000060656	2.04	PTPRU
ENSG00000184056	2.04	VPS33B
ENSG00000128335	2.04	APOL2
ENSG00000204946	2.04	ZNF783
ENSG00000132170	2.04	PPARG
ENSG00000163121	2.04	NEURL3
ENSG00000150275	2.04	PCDH15
ENSG00000165757	2.04	JCAD
ENSG00000179021	2.04	C3orf38
ENSG00000257534	2.04	
ENSG00000259073	2.04	FOXN3-AS2
ENSG00000287191	2.04	
ENSG00000284624	2.04	
ENSG00000047315	2.04	POLR2B
ENSG00000169758	2.04	TMEM266
ENSG00000214900	2.04	LINC01588
ENSG00000240024	2.04	LINC00888
ENSG00000264443	2.04	
ENSG00000132561	2.04	MATN2
ENSG00000166033	2.04	HTRA1
ENSG00000123473	2.04	STIL
ENSG00000112578	2.04	BYSL
ENSG00000196388	2.04	INCA1
ENSG00000158480	2.05	SPATA2
ENSG00000164494	2.05	PDSS2
ENSG00000173227	2.05	SYT12
ENSG00000103343	2.05	ZNF174
ENSG00000272661	2.05	
ENSG00000119616	2.05	FCF1
ENSG00000272449	2.05	
ENSG00000231683	2.05	LOC101927136
ENSG00000179038	2.05	
ENSG00000226383	2.05	LINC01876
ENSG00000226415	2.05	

ENSG00000230358	2.05	
ENSG00000249065	2.05	
ENSG00000257494	2.05	
ENSG00000186994	2.05	KANK3
ENSG00000268912	2.05	
ENSG00000109586	2.05	GALNT7
ENSG00000272163	2.05	
ENSG00000100614	2.05	PPM1A
ENSG00000127533	2.05	F2RL3
ENSG00000091039	2.05	OSBPL8
ENSG00000205268	2.05	PDE7A
ENSG00000111897	2.05	SERINC1
ENSG00000237307	2.06	
ENSG00000005175	2.06	RPAP3
ENSG00000260805	2.06	
ENSG00000173418	2.06	NAA20
ENSG00000136940	2.06	PDCL
ENSG00000223478	2.06	LOC100506100
ENSG00000180011	2.06	ZADH2
ENSG00000158985	2.06	CDC42SE2
ENSG00000262919	2.06	FAM58A
ENSG00000230698	2.06	
ENSG00000168140	2.06	VASN
ENSG00000229851	2.06	
ENSG00000114993	2.06	RTKN
ENSG00000183506	2.06	PI4KAP2
ENSG00000110031	2.06	LPXN
ENSG00000132204	2.06	
ENSG00000228328	2.06	
ENSG00000176155	2.06	CCDC57
ENSG00000124357	2.06	NAGK
ENSG00000101986	2.06	ABCD1
ENSG00000225507	2.06	
ENSG00000101654	2.07	RNMT
ENSG00000076043	2.07	REXO2
ENSG00000151332	2.07	MBIP
ENSG00000237101	2.07	LOC101927164
ENSG00000102981	2.07	PARD6A
ENSG00000197595	2.07	
ENSG00000220008	2.07	LINGO3
ENSG00000234043	2.07	
ENSG00000248112	2.07	

ENSG00000258134	2.07	
ENSG00000264644	2.07	
ENSG00000274351	2.07	
ENSG00000175591	2.07	P2RY2
ENSG00000132434	2.07	LANCL2
ENSG00000128463	2.07	EMC4
ENSG00000171004	2.07	HS6ST2
ENSG00000198000	2.07	NOL8
ENSG00000243989	2.07	ACY1
ENSG00000110080	2.07	ST3GAL4
ENSG00000100196	2.07	KDELR3
ENSG00000267056	2.07	
ENSG00000125812	2.07	GZF1
ENSG00000156931	2.07	VPS8
ENSG00000116455	2.08	WDR77
ENSG00000140254	2.08	DUOXA1
ENSG00000114796	2.08	KLHL24
ENSG00000153214	2.08	TMEM87B
ENSG00000196917	2.08	HCAR1
ENSG00000131969	2.08	ABHD12B
ENSG00000135617	2.08	PRADC1
ENSG00000197617	2.08	VN1R5
ENSG00000251288	2.08	
ENSG00000268460	2.08	LOC93429
ENSG00000288692	2.08	
ENSG00000285219	2.08	
ENSG00000134153	2.08	EMC7
ENSG00000184922	2.08	FMNL1
ENSG00000168476	2.08	REEP4
ENSG00000068383	2.08	INPP5A
ENSG00000153391	2.08	INO80C
ENSG00000108379	2.08	LOC101929777
ENSG00000110429	2.08	FBXO3
ENSG00000109466	2.09	KLHL2
ENSG00000156831	2.09	NSMCE2
ENSG00000166326	2.09	TRIM44
ENSG00000090989	2.09	EXOC1
ENSG00000106609	2.09	TMEM248
ENSG00000157106	2.09	SMG1
ENSG00000165091	2.09	TMC1
ENSG00000259494	2.09	MRPL46
ENSG00000272419	2.09	LOC103091866

ENSG00000178573	2.09	MAF
ENSG00000138375	2.09	SMARCAL1
ENSG00000131127	2.09	ZNF141
ENSG00000254783	2.09	
ENSG00000286165	2.09	
ENSG00000044090	2.09	CUL7
ENSG00000215910	2.09	C1orf167
ENSG00000120832	2.09	MTERF2
ENSG00000181704	2.09	YIPF6
ENSG00000167840	2.09	ZNF232
ENSG00000135900	2.10	MRPL44
ENSG00000125741	2.10	OPA3
ENSG00000167543	2.10	TP53I13
ENSG00000021776	2.10	AQR
ENSG00000173175	2.10	ADCY5
ENSG00000187595	2.10	
ENSG00000255154	2.10	
ENSG00000274363	2.10	
ENSG00000167333	2.10	TRIM68
ENSG00000136731	2.10	UGGT1
ENSG00000148225	2.10	WDR31
ENSG00000081791	2.10	KIAA0141
ENSG00000116711	2.10	PLA2G4A
ENSG00000279155	2.10	
ENSG00000167550	2.10	RHEBL1
ENSG00000234771	2.10	
ENSG00000184313	2.11	MROH7
ENSG00000070214	2.11	SLC44A1
ENSG00000257279	2.11	LOC100506551
ENSG00000111799	2.11	COL12A1
ENSG00000237399	2.11	PITRM1-AS1
ENSG00000168538	2.11	TRAPPC11
ENSG00000170262	2.11	MRAP
ENSG00000011677	2.11	GABRA3
ENSG00000273142	2.11	
ENSG00000147642	2.11	SYBU
ENSG00000111344	2.11	RASAL1
ENSG00000224934	2.11	
ENSG00000164117	2.11	FBXO8
ENSG00000047648	2.11	ARHGAP6
ENSG00000099937	2.11	SERPIND1
ENSG00000143184	2.11	XCL1

ENSG00000154175	2.11	ABI3BP
ENSG00000170577	2.11	SIX2
ENSG00000188322	2.11	SBK1
ENSG00000254775	2.11	
ENSG00000198252	2.11	STYX
ENSG00000166946	2.11	CCNDBP1
ENSG00000254902	2.11	
ENSG00000233515	2.11	
ENSG00000139988	2.11	RDH12
ENSG00000269858	2.11	EGLN2
ENSG00000112419	2.11	PHACTR2
ENSG00000275765	2.11	
ENSG00000179348	2.12	GATA2
ENSG00000182108	2.12	DEXI
ENSG00000152127	2.12	MGAT5
ENSG00000268087	2.12	
ENSG00000161642	2.12	ZNF385A
ENSG00000177951	2.12	BET1L
ENSG00000260577	2.12	
ENSG00000170049	2.12	KCNAB3
ENSG00000214046	2.12	
ENSG00000231822	2.12	
ENSG00000256399	2.12	
ENSG00000287437	2.12	
ENSG00000064195	2.12	DLX3
ENSG00000278996	2.13	
ENSG00000137494	2.13	ANKRD42
ENSG00000235750	2.13	KIAA0040
ENSG00000198917	2.13	SPOUT1
ENSG00000157224	2.13	CLDN12
ENSG00000273387	2.13	
ENSG00000004864	2.13	SLC25A13
ENSG00000175348	2.13	TMEM9B
ENSG00000265073	2.13	
ENSG00000141994	2.13	DUS3L
ENSG00000136861	2.13	CDK5RAP2
ENSG00000243955	2.13	GSTA1
ENSG00000266904	2.13	
ENSG00000287906	2.13	
ENSG00000011258	2.13	MBTD1
ENSG00000159322	2.13	ADPGK
ENSG00000073050	2.13	XRCC1

ENSG00000272288	2.13	LOC101929243
ENSG00000185522	2.13	LMNTD2
ENSG00000171109	2.13	MFN1
ENSG00000260563	2.13	
ENSG00000127419	2.13	TMEM175
ENSG00000185164	2.13	LOC102723728
ENSG00000100154	2.13	TTC28
ENSG00000144659	2.14	SLC25A38
ENSG00000149582	2.14	TMEM25
ENSG00000276570	2.14	
ENSG00000057704	2.14	TMCC3
ENSG00000149196	2.14	HIKESHI
ENSG00000205559	2.14	CHKB-AS1
ENSG00000104848	2.14	KCNA7
ENSG00000164663	2.14	USP49
ENSG00000224221	2.14	
ENSG00000259211	2.14	
ENSG00000261094	2.14	
ENSG00000270292	2.14	
ENSG00000095587	2.14	TLL2
ENSG00000268199	2.14	
ENSG00000092820	2.14	EZR
ENSG00000136867	2.14	SLC31A2
ENSG00000083123	2.14	BCKDHB
ENSG00000054116	2.14	TRAPPC3
ENSG00000132386	2.14	SERPINF1
ENSG00000259763	2.14	
ENSG00000188095	2.14	MESP2
ENSG00000106733	2.14	NMRK1
ENSG00000164086	2.14	DUSP7
ENSG00000183840	2.14	GPR39
ENSG00000245937	2.15	LINC01184
ENSG00000163431	2.15	LMOD1
ENSG00000235351	2.15	
ENSG00000272916	2.15	
ENSG00000280334	2.15	
ENSG00000123815	2.15	COQ8B
ENSG00000114450	2.15	GNB4
ENSG00000124788	2.15	ATXN1
ENSG00000120688	2.15	WBP4
ENSG00000136206	2.15	SPDYE1
ENSG00000184709	2.15	LRRC26

ENSG00000240477	2.15	
ENSG00000102606	2.15	ARHGEF7
ENSG00000146576	2.15	C7orf26
ENSG00000163064	2.15	EN1
ENSG00000087263	2.15	OGFOD1
ENSG00000233885	2.15	
ENSG00000171160	2.15	MORN4
ENSG00000280193	2.15	
ENSG00000280032	2.15	
ENSG00000245648	2.15	LOC101928100
ENSG00000167964	2.16	RAB26
ENSG00000182197	2.16	EXT1
ENSG00000082898	2.16	XPO1
ENSG00000263072	2.16	
ENSG00000258610	2.16	
ENSG00000095637	2.16	SORBS1
ENSG00000174007	2.16	CEP19
ENSG00000213781	2.16	
ENSG00000234562	2.16	
ENSG00000235253	2.16	
ENSG00000250546	2.16	LOC101928978
ENSG00000255872	2.16	
ENSG00000274925	2.16	
ENSG00000287896	2.16	
ENSG00000163898	2.16	LIPH
ENSG00000235703	2.16	LINC00894
ENSG00000285437	2.16	
ENSG00000147421	2.16	HMBOX1
ENSG00000256028	2.16	
ENSG00000170325	2.16	PRDM10
ENSG00000130720	2.16	FIBCD1
ENSG00000235954	2.16	TTC28-AS1
ENSG00000123636	2.16	
ENSG00000155090	2.16	KLF10
ENSG00000014123	2.17	UFL1
ENSG00000103994	2.17	ZNF106
ENSG00000183323	2.17	CCDC125
ENSG00000113273	2.17	ARSB
ENSG00000065621	2.17	GSTO2
ENSG00000092421	2.17	SEMA6A
ENSG00000132854	2.17	KANK4
ENSG00000183034	2.17	OTOP2

ENSG00000213089	2.17	
ENSG00000233485	2.17	
ENSG00000241170	2.17	
ENSG00000260494	2.17	
ENSG00000281974	2.17	
ENSG00000285770	2.17	
ENSG00000288618	2.17	
ENSG00000168779	2.17	SHOX2
ENSG00000106086	2.17	PLEKHA8
ENSG00000113580	2.17	NR3C1
ENSG00000228742	2.17	
ENSG00000265194	2.17	
ENSG00000057149	2.18	SERPINB3
ENSG00000152475	2.18	ZNF837
ENSG00000124225	2.18	PMEPA1
ENSG00000230185	2.18	
ENSG00000280649	2.18	
ENSG00000146350	2.18	TBC1D32
ENSG00000174899	2.18	PQLC2L
ENSG00000215127	2.18	
ENSG00000106327	2.18	TFR2
ENSG00000147400	2.18	CETN2
ENSG00000164708	2.18	PGAM2
ENSG00000268854	2.18	
ENSG00000138246	2.18	DNAJC13
ENSG00000137274	2.18	BPHL
ENSG00000071553	2.18	ATP6AP1
ENSG00000143322	2.19	ABL2
ENSG00000182541	2.19	LIMK2
ENSG00000103351	2.19	CLUAP1
ENSG00000151014	2.19	NOCT
ENSG00000130856	2.19	ZNF236
ENSG00000271625	2.19	
ENSG00000179046	2.19	TRIML2
ENSG00000213316	2.19	LTC4S
ENSG00000204174	2.19	NPY4R
ENSG00000119242	2.19	CCDC92
ENSG00000119661	2.19	DNAL1
ENSG00000119777	2.19	TMEM214
ENSG00000233825	2.19	
ENSG00000185689	2.19	C6orf201
ENSG00000124786	2.19	SLC35B3

ENSG00000175592	2.20	FOSL1
ENSG00000088854	2.20	C20orf194
ENSG00000085719	2.20	CPNE3
ENSG00000173614	2.20	NMNAT1
ENSG00000054983	2.20	GALC
ENSG00000165240	2.20	ATP7A
ENSG00000261061	2.20	
ENSG00000286877	2.20	
ENSG00000131791	2.20	PRKAB2
ENSG00000151092	2.20	NGLY1
ENSG00000167264	2.20	DUS2
ENSG00000197296	2.20	FITM2
ENSG00000188897	2.21	LOC400499
ENSG00000231770	2.21	
ENSG00000206560	2.21	ANKRD28
ENSG00000168818	2.21	STX18
ENSG00000172460	2.21	PRSS30P
ENSG00000127412	2.21	TRPV5
ENSG00000130592	2.21	LSP1
ENSG00000140025	2.21	EFCAB11
ENSG00000185988	2.21	PLK5
ENSG00000214286	2.21	
ENSG00000227660	2.21	
ENSG00000250127	2.21	LINC02108
ENSG00000166224	2.21	SGPL1
ENSG00000165886	2.21	UBTD1
ENSG00000228915	2.21	
ENSG00000186312	2.21	CA5BP1
ENSG00000143627	2.21	PKLR
ENSG00000258539	2.21	
ENSG00000123358	2.21	NR4A1
ENSG00000165105	2.21	RASEF
ENSG00000115756	2.22	HPCAL1
ENSG00000285967	2.22	
ENSG00000241570	2.22	PAQR9-AS1
ENSG00000108622	2.22	ICAM2
ENSG00000138641	2.22	LOC101929134
ENSG00000151687	2.22	ANKAR
ENSG00000213654	2.22	GPSM3
ENSG00000230699	2.22	
ENSG00000235806	2.22	
ENSG00000249803	2.22	

ENSG00000254558	2.22	
ENSG00000102316	2.22	MAGED2
ENSG00000169093	2.22	ASMTL
ENSG00000274791	2.22	F8A2
ENSG00000215769	2.22	ARHGAP27P1
ENSG00000228594	2.23	FNDC10
ENSG00000130939	2.23	UBE4B
ENSG00000124313	2.23	IQSEC2
ENSG00000204511	2.23	MCCD1
ENSG00000204611	2.23	ZNF616
ENSG00000215014	2.23	
ENSG00000230371	2.23	
ENSG00000269242	2.23	
ENSG00000087095	2.23	NLK
ENSG00000260425	2.23	
ENSG00000236283	2.23	
ENSG00000089154	2.23	GCN1
ENSG00000185436	2.23	IFNLR1
ENSG00000080815	2.23	PSEN1
ENSG00000172508	2.24	CARNS1
ENSG00000139200	2.24	PIANP
ENSG00000109184	2.24	DCUN1D4
ENSG00000265666	2.24	RARA-AS1
ENSG00000137411	2.24	VARS2
ENSG00000197021	2.24	CXorf40B
ENSG00000119866	2.24	BCL11A
ENSG00000164853	2.24	UNCX
ENSG00000272843	2.24	
ENSG00000167081	2.24	PBX3
ENSG00000113595	2.24	TRIM23
ENSG00000167107	2.24	ACSF2
ENSG00000103591	2.24	AAGAB
ENSG00000203896	2.24	LIME1
ENSG00000274659	2.24	
ENSG00000140157	2.24	NIPA2
ENSG00000137414	2.25	FAM8A1
ENSG00000175879	2.25	HOXD8
ENSG00000103723	2.25	AP3B2
ENSG00000255404	2.25	
ENSG00000163884	2.25	KLF15
ENSG00000237025	2.25	
ENSG00000276900	2.25	

ENSG00000131389	2.25	SLC6A6
ENSG00000051128	2.25	HOMER3
ENSG00000174206	2.25	C12orf66
ENSG00000168152	2.25	THAP9
ENSG00000256683	2.25	ZNF350
ENSG00000224531	2.26	SMIM13
ENSG00000271383	2.26	NBPF19
ENSG00000123268	2.26	ATF1
ENSG00000052850	2.26	ALX4
ENSG00000106819	2.26	ASPN
ENSG00000120217	2.26	CD274
ENSG00000176428	2.26	VPS37D
ENSG00000184227	2.26	ACOT1
ENSG00000226261	2.26	
ENSG00000228836	2.26	CT45A5
ENSG00000261105	2.26	LMO7-AS1
ENSG00000262488	2.26	
ENSG00000269720	2.26	LOC105372343
ENSG00000283886	2.26	
ENSG00000156876	2.26	SASS6
ENSG00000261373	2.26	VPS9D1-AS1
ENSG00000163053	2.26	SLC16A14
ENSG00000198951	2.26	NAGA
ENSG00000231806	2.26	PCAT7
ENSG00000197361	2.27	FBXL22
ENSG00000164051	2.27	CCDC51
ENSG00000075399	2.27	VPS9D1
ENSG00000284526	2.27	
ENSG00000196372	2.27	ASB13
ENSG00000113441	2.27	LNPEP
ENSG00000148158	2.27	SNX30
ENSG00000286974	2.27	
ENSG00000141858	2.27	SAMD1
ENSG00000087903	2.27	RFX2
ENSG00000229186	2.27	
ENSG00000123595	2.27	RAB9A
ENSG00000173083	2.27	HPSE
ENSG00000118579	2.27	MED28
ENSG00000104388	2.27	RAB2A
ENSG00000196141	2.27	SPATS2L
ENSG00000130675	2.27	MNX1
ENSG00000185798	2.28	WDR53

ENSG00000196189	2.28	SEMA4A
ENSG00000278594	2.28	
ENSG00000180919	2.28	OR56B4
ENSG00000276727	2.28	
ENSG00000157020	2.28	SEC13
ENSG00000075643	2.28	MOCOS
ENSG00000116539	2.28	ASH1L
ENSG00000131620	2.28	ANO1
ENSG00000100938	2.29	GMPR2
ENSG00000172785	2.29	CBWD1
ENSG00000137710	2.29	RDX
ENSG00000268069	2.29	
ENSG00000251201	2.29	TMED7-TICAM2
ENSG00000101204	2.29	CHRNA4
ENSG00000144847	2.29	IGSF11
ENSG00000233225	2.29	
ENSG00000205913	2.29	SRRM2-AS1
ENSG00000225361	2.29	PPP1R26-AS1
ENSG00000156411	2.29	C14orf2
ENSG00000106683	2.29	LIMK1
ENSG00000112186	2.30	CAP2
ENSG00000153246	2.30	PLA2R1
ENSG00000169429	2.30	CXCL8
ENSG00000174450	2.30	GOLGA6L2
ENSG00000180539	2.30	
ENSG00000230046	2.30	
ENSG00000274173	2.30	
ENSG00000287503	2.30	
ENSG00000253710	2.30	ALG11
ENSG00000132623	2.30	ANKEF1
ENSG00000101367	2.31	MAPRE1
ENSG00000111077	2.31	TNS2
ENSG00000141642	2.31	ELAC1
ENSG00000272931	2.31	
ENSG00000158055	2.31	GRHL3
ENSG00000143614	2.31	GATAD2B
ENSG00000152818	2.31	UTRN
ENSG00000164850	2.31	GPER1
ENSG00000138138	2.31	ATAD1
ENSG00000239607	2.32	
ENSG00000181284	2.32	TMEM102
ENSG00000107864	2.32	CPEB3

ENSG00000164306	2.32	PRIMPOL
ENSG00000136152	2.32	COG3
ENSG00000126005	2.32	
ENSG00000226742	2.32	HSBP1L1
ENSG00000140854	2.32	KATNB1
ENSG00000261487	2.32	
ENSG00000224652	2.32	LINC00885
ENSG00000261044	2.32	
ENSG00000188994	2.32	ZNF292
ENSG00000075234	2.32	TTC38
ENSG00000154582	2.32	ELOC
ENSG00000185482	2.32	STAC3
ENSG00000141562	2.33	NARF
ENSG00000118058	2.33	KMT2A
ENSG00000011590	2.33	ZBTB32
ENSG00000065615	2.33	CYB5R4
ENSG00000156313	2.33	RPGR
ENSG00000173567	2.33	ADGRF3
ENSG00000227582	2.33	
ENSG00000235296	2.33	
ENSG00000241560	2.33	ZBTB20-AS1
ENSG00000274461	2.33	
ENSG00000279434	2.33	
ENSG00000144591	2.33	GMPPA
ENSG00000152700	2.33	SAR1B
ENSG00000141441	2.33	GAREM1
ENSG00000204767	2.33	FAM196B
ENSG00000185158	2.33	LRRC37B
ENSG00000262246	2.33	CORO7
ENSG00000230612	2.33	
ENSG00000125746	2.34	EML2
ENSG00000086548	2.34	CEACAM6
ENSG00000064547	2.34	LPAR2
ENSG00000259539	2.34	
ENSG00000260394	2.34	LOC105371184
ENSG00000177683	2.34	THAP5
ENSG00000270055	2.34	
ENSG00000085377	2.34	PREP
ENSG00000261460	2.34	
ENSG00000141298	2.34	SSH2
ENSG00000169609	2.34	C15orf40
ENSG00000127540	2.34	

ENSG00000008869	2.34	HEATR5B
ENSG00000279032	2.34	
ENSG00000137193	2.34	PIM1
ENSG00000137075	2.35	RNF38
ENSG00000197980	2.35	LEKR1
ENSG00000111358	2.35	GTF2H3
ENSG00000287408	2.35	
ENSG00000229980	2.35	TOB1-AS1
ENSG00000280055	2.35	TMEM75
ENSG00000280063	2.35	
ENSG00000105793	2.35	LOC101927446
ENSG00000155158	2.35	TTC39B
ENSG00000172331	2.35	BPGM
ENSG00000253549	2.35	CA3-AS1
ENSG00000264673	2.35	
ENSG00000270426	2.35	
ENSG00000279408	2.35	
ENSG00000115760	2.35	BIRC6
ENSG00000099822	2.35	HCN2
ENSG00000163093	2.35	BBS5
ENSG00000232774	2.35	FLJ22447
ENSG00000134318	2.35	ROCK2
ENSG00000116741	2.35	RGS2
ENSG00000121680	2.36	PEX16
ENSG00000178297	2.36	TMPRSS9
ENSG00000283667	2.36	
ENSG00000248923	2.36	
ENSG00000186076	2.36	
ENSG00000149541	2.36	B3GAT3
ENSG00000172086	2.36	KRCC1
ENSG00000221990	2.36	EXOC3-AS1
ENSG00000251602	2.36	LOC100507437
ENSG00000187808	2.36	SOWAHD
ENSG00000140987	2.36	ZSCAN32
ENSG00000239789	2.36	MRPS17
ENSG00000037280	2.36	FLT4
ENSG00000113615	2.36	SEC24A
ENSG00000155265	2.36	GOLGA7B
ENSG00000229433	2.36	LINC02069
ENSG00000273542	2.36	HIST1H4K
ENSG00000123545	2.36	NDUFAF4
ENSG00000204852	2.36	TCTN1

ENSG00000075239	2.36	ACAT1
ENSG00000130751	2.36	NPAS1
ENSG00000286623	2.36	
ENSG00000203705	2.36	TATDN3
ENSG00000182934	2.36	SRPRA
ENSG00000260274	2.36	
ENSG00000204160	2.37	ZDHHC18
ENSG00000217733	2.37	
ENSG00000185838	2.37	GNB1L
ENSG00000212123	2.37	PRR22
ENSG00000227471	2.37	AKR1B15
ENSG00000255507	2.37	
ENSG00000259165	2.37	
ENSG00000267441	2.37	
ENSG00000277646	2.37	
ENSG00000277879	2.37	
ENSG00000279459	2.37	
ENSG00000156639	2.37	ZFAND3
ENSG00000258136	2.37	LOC101929162
ENSG00000254473	2.37	LOC105376114
ENSG00000263624	2.37	
ENSG00000088387	2.37	DOCK9
ENSG00000130150	2.37	MOSPD2
ENSG00000128609	2.37	NDUFA5
ENSG00000175279	2.37	CENPS
ENSG00000271699	2.38	
ENSG00000151176	2.38	PLBD2
ENSG00000169740	2.38	ZNF32
ENSG00000091164	2.38	TXNL1
ENSG00000237669	2.38	
ENSG00000232457	2.38	
ENSG00000203995	2.38	ZYG11A
ENSG00000115526	2.38	CHST10
ENSG00000162999	2.38	DUSP19
ENSG00000268947	2.38	
ENSG00000272143	2.38	FGF14-AS2
ENSG00000234983	2.38	
ENSG00000270554	2.38	
ENSG00000110799	2.38	VWF
ENSG00000138115	2.38	CYP2C8
ENSG00000256812	2.38	CAPNS2
ENSG00000163472	2.38	TMEM79

ENSG00000196458	2.38	ZNF605
ENSG00000181085	2.38	MAPK15
ENSG00000169891	2.39	REPS2
ENSG00000148450	2.39	MSRB2
ENSG00000215788	2.39	TNFRSF25
ENSG00000168778	2.39	TCTN2
ENSG00000092529	2.39	CAPN3
ENSG00000119778	2.39	ATAD2B
ENSG00000114784	2.39	EIF1B
ENSG00000149639	2.39	SOGA1
ENSG00000255158	2.39	
ENSG00000172006	2.39	ZNF554
ENSG00000182985	2.39	CADM1
ENSG00000236323	2.39	
ENSG00000272247	2.39	
ENSG00000272501	2.39	
ENSG00000119705	2.39	SLIRP
ENSG00000141294	2.39	LRRC46
ENSG00000065559	2.39	MAP2K4
ENSG00000111012	2.39	CYP27B1
ENSG00000069943	2.40	PIGB
ENSG00000243431	2.40	
ENSG00000213809	2.40	KLRK1
ENSG00000149499	2.40	EML3
ENSG00000174227	2.40	PIGG
ENSG00000204253	2.40	
ENSG00000158796	2.40	DEDD
ENSG00000166797	2.40	FAM96A
ENSG00000183648	2.40	NDUFB1
ENSG00000172476	2.40	RAB40A
ENSG00000223808	2.40	
ENSG00000231760	2.40	
ENSG00000137764	2.40	MAP2K5
ENSG00000214770	2.40	
ENSG00000066654	2.40	THUMPD1
ENSG00000144369	2.40	FAM171B
ENSG00000258048	2.40	
ENSG00000181027	2.40	FKRP
ENSG00000165923	2.41	AGBL2
ENSG00000272554	2.41	
ENSG00000114573	2.41	ATP6V1A
ENSG00000003402	2.41	CFLAR

ENSG00000112343	2.41	TRIM38
ENSG00000143878	2.41	RHOB
ENSG00000261528	2.41	
ENSG00000213757	2.41	
ENSG00000258917	2.41	
ENSG00000196199	2.41	MPHOSPH8
ENSG00000278973	2.41	
ENSG00000188760	2.41	TMEM198
ENSG00000269293	2.41	ZSCAN16-AS1
ENSG00000108799	2.41	EZH1
ENSG00000281016	2.41	
ENSG00000149634	2.41	SPATA25
ENSG00000283131	2.41	
ENSG00000285884	2.41	
ENSG00000129038	2.41	LOXL1
ENSG00000167741	2.41	GGT6
ENSG00000183570	2.41	PCBP3
ENSG00000104442	2.41	ARMC1
ENSG00000265750	2.41	
ENSG00000245711	2.41	
ENSG00000110944	2.42	IL23A
ENSG00000259026	2.42	
ENSG00000286102	2.42	
ENSG00000270036	2.42	
ENSG00000151247	2.42	EIF4E
ENSG00000272097	2.42	
ENSG00000271662	2.42	
ENSG00000134030	2.42	CTIF
ENSG00000267277	2.42	
ENSG00000279590	2.42	
ENSG00000214725	2.42	CDIPT-AS1
ENSG00000287921	2.42	
ENSG00000025708	2.42	TYMP
ENSG00000163216	2.42	SPRR2D
ENSG00000254996	2.42	ANKHD1-EIF4EBP3
ENSG00000204351	2.43	SKIV2L
ENSG00000273183	2.43	
ENSG00000167705	2.43	RILP
ENSG00000271133	2.43	LOC101927811
ENSG00000236935	2.43	
ENSG00000167360	2.43	OR51Q1
ENSG00000173838	2.43	MARCHF10

ENSG00000236819	2.43	LINC01563
ENSG00000261302	2.43	LOC105371286
ENSG00000130748	2.43	TMEM160
ENSG00000259732	2.43	
ENSG00000281538	2.43	
ENSG00000234699	2.43	
ENSG00000248863	2.43	
ENSG00000106992	2.44	AK1
ENSG00000237938	2.44	
ENSG00000254614	2.44	LOC728975
ENSG00000110906	2.44	KCTD10
ENSG00000260895	2.44	
ENSG00000271623	2.44	
ENSG00000224758	2.44	LINC01167
ENSG00000184828	2.44	ZBTB7C
ENSG00000224493	2.44	
ENSG00000240137	2.44	ERICH6-AS1
ENSG00000253161	2.44	
ENSG00000261079	2.44	LOC440386
ENSG00000285053	2.44	
ENSG00000012963	2.44	UBR7
ENSG00000171017	2.44	LRRC8E
ENSG00000100036	2.44	SLC35E4
ENSG00000157873	2.44	TNFRSF14
ENSG00000101745	2.44	ANKRD12
ENSG00000271843	2.44	
ENSG00000049883	2.44	PTCD2
ENSG00000276488	2.44	
ENSG00000186073	2.44	C15orf41
ENSG00000119862	2.45	LGALSL
ENSG00000105227	2.45	PRX
ENSG00000164073	2.45	MFSD8
ENSG00000259684	2.45	
ENSG00000174718	2.45	KIAA1551
ENSG00000147257	2.45	GPC3
ENSG00000218227	2.45	
ENSG00000270614	2.45	
ENSG00000278991	2.45	
ENSG00000235335	2.45	
ENSG00000083290	2.45	ULK2
ENSG00000187231	2.45	SESTD1
ENSG00000267827	2.45	

ENSG00000169504	2.45	CLIC4
ENSG00000214517	2.45	PPME1
ENSG00000117262	2.45	GPR89A
ENSG00000214140	2.46	PRCD
ENSG00000174738	2.46	NR1D2
ENSG00000253559	2.46	
ENSG00000116874	2.46	WARS2
ENSG00000119912	2.46	IDE
ENSG00000172554	2.46	SNTG2
ENSG00000223349	2.46	
ENSG00000235407	2.46	CYMP-AS1
ENSG00000258044	2.46	
ENSG00000268120	2.46	
ENSG00000286677	2.46	
ENSG00000268756	2.46	
ENSG00000099992	2.46	TBC1D10A
ENSG00000224875	2.46	
ENSG00000258521	2.46	
ENSG00000233998	2.46	
ENSG00000101665	2.46	SMAD7
ENSG00000258851	2.46	
ENSG00000171928	2.46	TVP23B
ENSG00000268605	2.46	
ENSG00000272936	2.46	
ENSG00000100372	2.47	SLC25A17
ENSG00000278390	2.47	LOC101929140
ENSG00000163635	2.47	ATXN7
ENSG00000228404	2.47	
ENSG00000278238	2.47	
ENSG00000166340	2.47	TPP1
ENSG00000167565	2.47	SERTAD3
ENSG00000258439	2.47	
ENSG00000140876	2.47	NUDT7
ENSG00000275560	2.47	
ENSG00000145685	2.47	LHFPL2
ENSG00000186891	2.47	TNFRSF18
ENSG00000196275	2.47	GTF2IRD2
ENSG00000125629	2.47	INSIG2
ENSG00000160233	2.47	LRRC3
ENSG00000267030	2.47	
ENSG00000227692	2.47	
ENSG00000217644	2.47	

ENSG00000267480	2.48	
ENSG00000164403	2.48	SHROOM1
ENSG00000135924	2.48	DNAJB2
ENSG00000139626	2.48	ITGB7
ENSG00000277182	2.48	LOC100287808
ENSG00000115705	2.48	TPO
ENSG00000145040	2.48	UCN2
ENSG00000177096	2.48	FAM109B
ENSG00000185758	2.48	CLDN24
ENSG00000267774	2.48	
ENSG00000285646	2.48	
ENSG00000185774	2.48	KCNIP4
ENSG00000243147	2.48	MRPL33
ENSG00000075292	2.48	ZNF638
ENSG00000224138	2.48	
ENSG00000197818	2.48	SLC9A8
ENSG00000230202	2.48	
ENSG00000267284	2.48	
ENSG00000131943	2.48	C19orf12
ENSG00000227678	2.49	
ENSG00000260755	2.49	
ENSG00000124587	2.49	PEX6
ENSG00000259955	2.49	
ENSG00000189001	2.49	SBSN
ENSG00000196668	2.49	LINC00173
ENSG00000149150	2.49	SLC43A1
ENSG00000124713	2.49	GNMT
ENSG00000149646	2.49	CNBD2
ENSG00000271888	2.49	
ENSG00000285665	2.49	
ENSG00000263164	2.49	
ENSG00000158805	2.49	ZNF276
ENSG00000138380	2.49	CARF
ENSG00000143093	2.49	STRIP1
ENSG00000204070	2.49	SYS1
ENSG00000149527	2.49	PLCH2
ENSG00000175595	2.50	ERCC4
ENSG00000096093	2.50	EFHC1
ENSG00000272669	2.50	
ENSG00000021355	2.50	SERPINB1
ENSG00000157216	2.50	SSBP3
ENSG00000163171	2.50	CDC42EP3

ENSG00000184058	2.50	TBX1
ENSG00000155755	2.50	TMEM237
ENSG00000267322	2.50	SNHG22
ENSG00000279465	2.50	
ENSG00000258646	2.50	
ENSG00000163900	2.50	TMEM41A
ENSG00000188660	2.50	LINC00319
ENSG00000183597	2.50	TANGO2
ENSG00000219039	2.50	
ENSG00000235034	2.51	C19orf81
ENSG00000184108	2.51	TRIML1
ENSG00000234619	2.51	
ENSG00000236449	2.51	
ENSG00000253875	2.51	
ENSG00000272056	2.51	
ENSG00000277247	2.51	
ENSG00000055609	2.51	KMT2C
ENSG00000173253	2.52	DMRT2
ENSG00000230750	2.52	
ENSG00000175322	2.52	ZNF519
ENSG00000176826	2.52	FKBP9P1
ENSG00000198538	2.52	ZNF28
ENSG00000257310	2.52	
ENSG00000279894	2.52	
ENSG00000185049	2.52	NELFA
ENSG00000229870	2.52	
ENSG00000179152	2.52	TCAIM
ENSG00000085552	2.53	IGSF9
ENSG00000115596	2.53	WNT6
ENSG00000267092	2.53	
ENSG00000070404	2.53	FSTL3
ENSG00000261349	2.53	
ENSG00000167123	2.53	CERCAM
ENSG00000125398	2.53	SOX9
ENSG00000163104	2.53	SMARCAD1
ENSG00000223510	2.53	CDRT15
ENSG00000243250	2.53	
ENSG00000259499	2.53	
ENSG00000284772	2.53	
ENSG00000287009	2.53	
ENSG00000149313	2.53	AASDHPPT
ENSG00000154305	2.53	MIA3

ENSG00000105127	2.53	AKAP8
ENSG00000065183	2.54	WDR3
ENSG00000173991	2.54	TCAP
ENSG00000186281	2.54	GPAT2
ENSG00000213699	2.54	SLC35F6
ENSG00000173542	2.54	MOB1B
ENSG00000220506	2.54	
ENSG00000232702	2.54	
ENSG00000243710	2.54	CFAP57
ENSG00000255150	2.54	EID3
ENSG00000267691	2.54	
ENSG00000286835	2.54	
ENSG00000276529	2.54	
ENSG00000148343	2.54	MIGA2
ENSG00000153558	2.55	FBXL2
ENSG00000286555	2.55	
ENSG00000197579	2.55	TOPORS
ENSG00000219249	2.55	
ENSG00000243224	2.55	LOC101929054
ENSG00000261202	2.55	
ENSG00000133393	2.55	FOPNL
ENSG00000164930	2.55	FZD6
ENSG00000130595	2.55	TNNT3
ENSG00000140386	2.55	SCAPER
ENSG00000177854	2.56	TMEM187
ENSG00000162368	2.56	CMPK1
ENSG00000069509	2.56	FUNDC1
ENSG00000166592	2.56	RRAD
ENSG00000231439	2.56	WASIR2
ENSG00000236311	2.56	TLX1NB
ENSG00000164366	2.56	CCDC127
ENSG00000115942	2.57	ORC2
ENSG00000144857	2.57	BOC
ENSG00000237360	2.57	
ENSG00000174327	2.57	SLC16A13
ENSG00000161217	2.57	PCYT1A
ENSG00000164169	2.57	PRMT9
ENSG00000165555	2.57	NOXRED1
ENSG00000188585	2.57	CLEC20A
ENSG00000256222	2.57	MTRNR2L3
ENSG00000284981	2.57	
ENSG00000256115	2.57	LINC02443

ENSG00000257386	2.57	
ENSG00000177000	2.58	MTHFR
ENSG00000100558	2.58	PLEK2
ENSG00000149131	2.58	SERPING1
ENSG00000167711	2.58	SERPINF2
ENSG00000223485	2.58	LINC01615
ENSG00000226043	2.58	
ENSG00000232533	2.58	
ENSG00000248192	2.58	
ENSG00000257830	2.58	
ENSG00000280310	2.58	
ENSG00000170191	2.58	NANP
ENSG00000286305	2.58	
ENSG00000171121	2.58	KCNMB3
ENSG00000108958	2.59	
ENSG00000167515	2.59	TRAPPC2L
ENSG00000186732	2.59	MPPED1
ENSG00000135709	2.59	KIAA0513
ENSG00000277548	2.59	
ENSG00000103089	2.59	FA2H
ENSG00000106560	2.59	GIMAP2
ENSG00000126561	2.59	STAT5A
ENSG00000233668	2.59	
ENSG00000181830	2.59	SLC35C1
ENSG00000205420	2.59	KRT6A
ENSG00000118004	2.59	COLEC11
ENSG00000204439	2.59	C6orf47
ENSG00000121653	2.59	MAPK8IP1
ENSG00000139537	2.60	CCDC65
ENSG00000261104	2.60	
ENSG00000056050	2.60	HPF1
ENSG00000166016	2.60	ABTB2
ENSG00000014919	2.60	COX15
ENSG00000058056	2.60	USP13
ENSG00000260782	2.60	
ENSG00000106078	2.60	COBL
ENSG00000205978	2.60	NYNRIN
ENSG00000029639	2.60	TFB1M
ENSG00000081026	2.60	MAGI3
ENSG00000108510	2.61	MED13
ENSG00000066629	2.61	EML1
ENSG00000178814	2.61	OPLAH

ENSG00000227417	2.61	
ENSG00000288611	2.61	
ENSG00000123106	2.61	CCDC91
ENSG00000083937	2.61	CHMP2B
ENSG00000167371	2.61	PRRT2
ENSG00000178971	2.62	CTC1
ENSG00000179314	2.62	LOC339166
ENSG00000266495	2.62	
ENSG00000198722	2.62	UNC13B
ENSG00000102384	2.62	CENPI
ENSG00000148795	2.62	CYP17A1
ENSG00000227963	2.62	LOC440600
ENSG00000272031	2.62	ANKRD34A
ENSG00000099260	2.62	PALMD
ENSG00000188613	2.62	NANOS1
ENSG00000163874	2.62	ZC3H12A
ENSG00000089123	2.63	TASP1
ENSG00000177738	2.63	LOC648987
ENSG00000108590	2.63	MED31
ENSG00000175984	2.63	DENND2C
ENSG00000176490	2.63	DIRAS1
ENSG00000234362	2.63	LINC01914
ENSG00000236304	2.63	
ENSG00000272462	2.63	
ENSG00000226306	2.63	NPY6R
ENSG00000130021	2.63	PUDP
ENSG00000108433	2.63	
ENSG00000144043	2.64	TEX261
ENSG00000198711	2.64	SSBP3-AS1
ENSG00000277142	2.64	
ENSG00000134013	2.64	LOXL2
ENSG00000133103	2.64	COG6
ENSG00000224631	2.64	
ENSG00000254162	2.64	
ENSG00000255000	2.64	
ENSG00000285693	2.64	
ENSG00000164778	2.64	EN2
ENSG00000186866	2.64	POFUT2
ENSG00000078747	2.64	ITCH
ENSG00000106012	2.64	IQCE
ENSG00000185220	2.64	PGBD2
ENSG00000135249	2.65	RINT1

ENSG00000118263	2.65	KLF7
ENSG00000238164	2.65	
ENSG00000188811	2.65	NHLRC3
ENSG00000260790	2.65	
ENSG00000167034	2.65	NKX3-1
ENSG00000160613	2.65	PCSK7
ENSG00000139697	2.65	SBNO1
ENSG00000154914	2.65	USP43
ENSG00000114541	2.66	FRMD4B
ENSG00000073150	2.66	PANX2
ENSG00000224516	2.66	
ENSG00000258479	2.66	LINC00640
ENSG00000143891	2.66	GALM
ENSG00000134758	2.66	RNF138
ENSG00000170653	2.66	ATF7
ENSG00000109917	2.66	ZPR1
ENSG00000157483	2.67	MYO1E
ENSG00000163915	2.67	IGF2BP2-AS1
ENSG00000180190	2.67	TDRP
ENSG00000224961	2.67	LINC01752
ENSG00000225285	2.67	
ENSG00000253955	2.67	LOC285593
ENSG00000258611	2.67	
ENSG00000286019	2.67	
ENSG00000272054	2.67	
ENSG00000171314	2.67	PGAM1
ENSG00000156171	2.68	DRAM2
ENSG00000109794	2.68	FAM149A
ENSG00000124787	2.68	RPP40
ENSG00000135241	2.68	PNPLA8
ENSG00000245261	2.68	
ENSG00000164465	2.68	DCBLD1
ENSG00000158315	2.68	RHBDL2
ENSG00000182796	2.69	TMEM198B
ENSG00000260017	2.69	
ENSG00000228716	2.69	DHFR
ENSG00000137055	2.69	PLAA
ENSG00000166803	2.69	
ENSG00000145246	2.69	ATP10D
ENSG00000182134	2.69	TDRKH
ENSG00000122643	2.69	NT5C3A
ENSG00000279862	2.69	

ENSG00000251417	2.69	
ENSG00000163684	2.70	RPP14
ENSG00000165685	2.70	TMEM52B
ENSG00000122912	2.70	SLC25A16
ENSG00000228686	2.70	
ENSG00000169299	2.70	PGM2
ENSG00000135919	2.70	SERPINE2
ENSG00000050555	2.70	LAMC3
ENSG00000149177	2.71	PTPRJ
ENSG00000110172	2.71	CHORDC1
ENSG00000105613	2.71	MAST1
ENSG00000115137	2.71	DNAJC27
ENSG00000124549	2.71	BTN2A3P
ENSG00000143125	2.71	PROK1
ENSG00000108523	2.71	RNF167
ENSG00000095752	2.71	IL11
ENSG00000108349	2.71	CASC3
ENSG00000121067	2.71	SPOP
ENSG00000140057	2.72	AK7
ENSG00000233138	2.72	
ENSG00000238121	2.72	LINC00426
ENSG00000215256	2.72	DHRS4-AS1
ENSG00000038532	2.72	CLEC16A
ENSG00000196562	2.72	SULF2
ENSG00000115718	2.73	PROC
ENSG00000104497	2.73	SNX16
ENSG00000181392	2.73	SYNE4
ENSG00000237118	2.73	
ENSG00000248415	2.73	
ENSG00000273812	2.73	
ENSG00000197536	2.73	C5orf56
ENSG00000128394	2.73	APOBEC3F
ENSG00000165934	2.73	CPSF2
ENSG00000176998	2.73	
ENSG00000259856	2.74	
ENSG00000164081	2.74	TEX264
ENSG00000213539	2.74	
ENSG00000135069	2.74	PSAT1
ENSG00000039560	2.74	RAI14
ENSG00000166507	2.74	
ENSG00000071894	2.74	CPSF1
ENSG00000170802	2.74	FOXN2

ENSG00000286158	2.74	
ENSG00000049769	2.74	PPP1R3F
ENSG00000268996	2.75	MAN1B1-AS1
ENSG00000259715	2.75	
ENSG00000198855	2.75	FICD
ENSG00000204296	2.75	C6orf10
ENSG00000270696	2.75	
ENSG00000110851	2.75	PRDM4
ENSG00000169258	2.75	GPRIN1
ENSG00000213337	2.75	ANKRD39
ENSG00000205089	2.76	CCNI2
ENSG00000245552	2.76	LOC101929295
ENSG00000158467	2.76	AHCYL2
ENSG00000117461	2.76	
ENSG00000125954	2.76	CHURC1-FNTB
ENSG00000172575	2.76	RASGRP1
ENSG00000213888	2.76	
ENSG00000232882	2.76	
ENSG00000242109	2.76	
ENSG00000286001	2.76	
ENSG00000126500	2.76	FLRT1
ENSG00000124356	2.76	STAMBP
ENSG00000153207	2.77	AHCTF1
ENSG00000167699	2.77	GLOD4
ENSG00000139624	2.77	CERS5
ENSG00000102781	2.77	KATNAL1
ENSG00000107771	2.77	CCSER2
ENSG00000036672	2.77	USP2
ENSG00000265690	2.78	
ENSG00000267618	2.78	
ENSG00000271366	2.78	
ENSG00000287918	2.78	
ENSG00000288044	2.78	
ENSG00000107742	2.79	SPOCK2
ENSG00000258730	2.79	ITPK1-AS1
ENSG00000140022	2.79	STON2
ENSG00000273271	2.79	
ENSG00000111731	2.79	C2CD5
ENSG00000103202	2.80	NME4
ENSG00000228696	2.80	ARL17B
ENSG00000228021	2.80	LOC283038
ENSG00000119737	2.80	GPR75

ENSG00000216378	2.80	
ENSG00000248265	2.80	FLJ12825
ENSG00000250011	2.80	
ENSG00000276744	2.80	
ENSG00000083799	2.81	CYLD
ENSG00000203668	2.81	CHML
ENSG00000187583	2.81	PLEKHN1
ENSG00000064309	2.81	CDON
ENSG00000120049	2.81	KCNIP2
ENSG00000156009	2.81	MAGEA8
ENSG00000174038	2.81	C9orf131
ENSG00000228380	2.81	
ENSG00000230082	2.81	PRRT3-AS1
ENSG00000287555	2.81	
ENSG00000259604	2.81	
ENSG00000215067	2.81	ALOX12-AS1
ENSG00000261556	2.81	SMG1P7
ENSG00000258674	2.82	
ENSG00000163157	2.82	TMOD4
ENSG00000215712	2.82	TMEM242
ENSG00000234722	2.82	LINC01287
ENSG00000278206	2.82	
ENSG00000178852	2.82	EFCAB13
ENSG00000169220	2.82	RGS14
ENSG00000143369	2.82	ECM1
ENSG00000081014	2.83	AP4E1
ENSG00000115112	2.83	TFCP2L1
ENSG00000171916	2.83	LGALS9C
ENSG00000231764	2.83	
ENSG00000143995	2.83	MEIS1
ENSG00000147124	2.83	ZNF41
ENSG00000111540	2.84	RAB5B
ENSG00000171475	2.84	WIPF2
ENSG00000137210	2.84	TMEM14B
ENSG00000124641	2.84	MED20
ENSG00000206262	2.84	FOXL2NB
ENSG00000231468	2.84	
ENSG00000117533	2.84	VAMP4
ENSG00000104228	2.84	TRIM35
ENSG00000105519	2.84	CAPS
ENSG00000257075	2.85	
ENSG00000141646	2.85	SMAD4

ENSG00000162407	2.85	PLPP3
ENSG00000170092	2.85	SPDYE5
ENSG00000272711	2.85	
ENSG00000286799	2.85	
ENSG00000275964	2.85	
ENSG00000078124	2.85	ACER3
ENSG00000137033	2.85	IL33
ENSG00000087303	2.85	NID2
ENSG00000249007	2.85	
ENSG00000066739	2.85	ATG2B
ENSG00000155744	2.85	FAM126B
ENSG00000073464	2.86	CLCN4
ENSG00000163840	2.86	DTX3L
ENSG00000112763	2.86	BTN2A1
ENSG00000168246	2.86	UBTD2
ENSG00000064012	2.86	CASP8
ENSG00000107164	2.87	FUBP3
ENSG00000198015	2.87	MRPL42
ENSG00000135837	2.87	CEP350
ENSG00000184384	2.87	MAML2
ENSG00000131873	2.87	CHSY1
ENSG00000146143	2.87	PRIM2
ENSG00000155393	2.87	HEATR3
ENSG00000272991	2.87	
ENSG00000142166	2.87	IFNAR1
ENSG00000260588	2.88	
ENSG00000247199	2.88	LOC102546294
ENSG00000162645	2.88	GBP2
ENSG00000125814	2.88	NAPB
ENSG00000118961	2.88	LDAH
ENSG00000197582	2.88	
ENSG00000212664	2.88	
ENSG00000230013	2.88	
ENSG00000255526	2.88	NEDD8-MDP1
ENSG00000256664	2.88	
ENSG00000272695	2.88	GAS6-AS2
ENSG00000114529	2.88	C3orf52
ENSG00000262678	2.88	
ENSG00000139163	2.88	ETNK1
ENSG00000279322	2.89	
ENSG00000149308	2.89	NPAT
ENSG00000183474	2.89	GTF2H2C

ENSG00000079134	2.89	THOC1
ENSG00000146426	2.89	TIAM2
ENSG00000100626	2.89	GALNT16
ENSG00000261652	2.89	C15orf65
ENSG00000272798	2.89	
ENSG00000271147	2.89	
ENSG00000107077	2.89	KDM4C
ENSG00000135424	2.90	ITGA7
ENSG00000278831	2.90	
ENSG00000241114	2.90	
ENSG00000107643	2.90	MAPK8
ENSG00000169313	2.90	P2RY12
ENSG00000226942	2.90	
ENSG00000228386	2.90	
ENSG00000198625	2.90	MDM4
ENSG00000154814	2.90	OXNAD1
ENSG00000149212	2.90	SESN3
ENSG00000047188	2.91	YTHDC2
ENSG00000222043	2.91	
ENSG00000134001	2.91	EIF2S1
ENSG00000133106	2.91	EPSTI1
ENSG00000272666	2.91	
ENSG00000283526	2.91	LOC642515
ENSG00000182158	2.91	CREB3L2
ENSG00000253320	2.91	
ENSG00000140511	2.92	HAPLN3
ENSG00000108785	2.92	
ENSG00000260306	2.92	
ENSG00000288549	2.92	
ENSG00000099308	2.93	MAST3
ENSG00000124459	2.93	ZNF45
ENSG00000172493	2.93	AFF1
ENSG00000107937	2.93	GTPBP4
ENSG00000120949	2.93	TNFRSF8
ENSG00000277050	2.93	
ENSG00000283654	2.93	
ENSG00000286334	2.93	
ENSG00000260464	2.93	
ENSG00000204104	2.93	TRAF3IP1
ENSG00000110171	2.93	TRIM3
ENSG00000242247	2.93	ARFGAP3
ENSG00000256462	2.94	

ENSG00000229800	2.94	
ENSG00000100652	2.94	SLC10A1
ENSG00000108878	2.94	CACNG1
ENSG00000250892	2.94	
ENSG00000270276	2.94	HIST2H4B
ENSG00000270839	2.94	
ENSG00000183665	2.94	TRMT12
ENSG00000198843	2.94	SELENOT
ENSG00000267733	2.94	
ENSG00000167768	2.94	KRT1
ENSG00000139117	2.94	CPNE8
ENSG00000272512	2.94	
ENSG00000170485	2.94	NPAS2
ENSG00000179178	2.95	TMEM125
ENSG00000139083	2.95	ETV6
ENSG00000226469	2.95	
ENSG00000263968	2.95	
ENSG00000122203	2.95	KIAA1191
ENSG00000230091	2.95	
ENSG00000254503	2.95	
ENSG00000116205	2.95	TCEANC2
ENSG00000131979	2.96	GCH1
ENSG00000100031	2.96	GGT1
ENSG00000143507	2.96	DUSP10
ENSG00000165704	2.96	HPRT1
ENSG00000131871	2.96	SELENOS
ENSG00000276180	2.96	HIST1H4I
ENSG00000176641	2.96	RNF152
ENSG00000088970	2.96	KIZ
ENSG00000197429	2.96	IPP
ENSG00000196323	2.96	ZBTB44
ENSG00000225880	2.96	LINC00115
ENSG00000277895	2.96	
ENSG00000078043	2.96	PIAS2
ENSG00000100281	2.96	HMGXB4
ENSG00000144115	2.97	THNSL2
ENSG00000231010	2.97	
ENSG00000235162	2.97	C12orf75
ENSG00000223842	2.97	
ENSG00000231929	2.97	
ENSG00000268015	2.97	
ENSG00000271936	2.97	

ENSG00000160201	2.97	U2AF1
ENSG00000074657	2.97	ZNF532
ENSG00000136144	2.97	RCBTB1
ENSG00000145029	2.97	
ENSG00000185760	2.97	KCNQ5
ENSG00000158411	2.97	MITD1
ENSG00000164008	2.98	C1orf50
ENSG00000005882	2.98	PDK2
ENSG00000279182	2.98	
ENSG00000204528	2.98	PSORS1C3
ENSG00000250765	2.98	
ENSG00000272217	2.98	
ENSG00000167114	2.98	SLC27A4
ENSG00000075089	2.99	ACTR6
ENSG00000160305	2.99	DIP2A
ENSG00000177685	2.99	CRACR2B
ENSG00000229638	2.99	
ENSG00000118985	2.99	ELL2
ENSG00000164244	2.99	PRRC1
ENSG00000184371	2.99	CSF1
ENSG00000263644	3.00	
ENSG00000274064	3.00	
ENSG00000141699	3.00	RETREG3
ENSG00000137070	3.00	IL11RA
ENSG00000123219	3.00	CENPK
ENSG00000204531	3.00	POU5F1
ENSG00000102904	3.01	TSNAXIP1
ENSG00000099330	3.01	OCEL1
ENSG00000231704	3.01	
ENSG00000099960	3.01	SLC7A4
ENSG00000172296	3.01	SPTLC3
ENSG00000272602	3.01	ZNF595
ENSG00000111252	3.01	SH2B3
ENSG00000108669	3.01	CYTH1
ENSG00000089048	3.01	ESF1
ENSG00000117016	3.01	RIMS3
ENSG00000275854	3.02	
ENSG00000142748	3.02	FCN3
ENSG00000146842	3.02	TMEM209
ENSG00000275234	3.03	
ENSG00000187240	3.03	DYNC2H1
ENSG00000141378	3.03	PTRH2

ENSG00000048392	3.03	RRM2B
ENSG00000174963	3.03	ZIC4
ENSG00000175087	3.03	PDIK1L
ENSG00000176971	3.03	FIBIN
ENSG00000223361	3.03	
ENSG00000223509	3.03	
ENSG00000228998	3.03	
ENSG00000232024	3.03	
ENSG00000235927	3.03	NEXN-AS1
ENSG00000241661	3.03	
ENSG00000272688	3.03	
ENSG00000255135	3.04	
ENSG00000249437	3.04	NAIP
ENSG00000242797	3.04	
ENSG00000170688	3.04	
ENSG00000256250	3.04	
ENSG00000152939	3.04	MARVELD2
ENSG00000078295	3.05	ADCY2
ENSG00000106178	3.05	CCL24
ENSG00000219392	3.05	
ENSG00000147419	3.05	CCDC25
ENSG00000184060	3.06	ADAP2
ENSG00000235213	3.06	
ENSG00000197965	3.06	MPZL1
ENSG00000182004	3.06	SNRPE
ENSG00000132906	3.06	CASP9
ENSG00000164684	3.06	ZNF704
ENSG00000112679	3.06	DUSP22
ENSG00000143155	3.07	TIPRL
ENSG00000111481	3.07	COPZ1
ENSG00000122035	3.07	RASL11A
ENSG00000049540	3.07	ELN
ENSG00000241269	3.07	
ENSG00000274026	3.07	FAM27E3
ENSG00000122257	3.07	RBBP6
ENSG00000121742	3.07	GJB6
ENSG00000171227	3.07	TMEM37
ENSG00000108244	3.08	KRT23
ENSG00000205423	3.08	CNEP1R1
ENSG00000135049	3.08	AGTPBP1
ENSG00000103042	3.08	SLC38A7
ENSG00000018280	3.08	SLC11A1

ENSG00000149328	3.08	GLB1L2
ENSG00000167419	3.08	LPO
ENSG00000226413	3.08	
ENSG00000227201	3.08	
ENSG00000237214	3.08	
ENSG00000254976	3.08	
ENSG00000279122	3.08	
ENSG00000164933	3.08	SLC25A32
ENSG00000271335	3.08	
ENSG00000198961	3.09	PJA2
ENSG00000271933	3.09	
ENSG00000107796	3.09	ACTA2
ENSG00000151090	3.09	THRB
ENSG00000166510	3.09	CCDC68
ENSG00000167207	3.09	NOD2
ENSG00000259868	3.09	
ENSG00000271551	3.09	
ENSG00000160325	3.09	CACFD1
ENSG00000130829	3.09	DUSP9
ENSG00000144642	3.10	RBMS3
ENSG00000185013	3.10	NT5C1B
ENSG00000236998	3.10	
ENSG00000237691	3.10	
ENSG00000123104	3.10	ITPR2
ENSG00000104856	3.10	RELB
ENSG00000186104	3.10	CYP2R1
ENSG00000176358	3.11	TAC4
ENSG00000260884	3.11	
ENSG00000128510	3.11	CPA4
ENSG00000152422	3.11	XRCC4
ENSG00000244459	3.11	
ENSG00000270071	3.11	
ENSG00000270249	3.11	
ENSG00000282021	3.11	
ENSG00000184985	3.11	SORCS2
ENSG00000204539	3.11	CDSN
ENSG00000186814	3.11	ZSCAN30
ENSG00000131016	3.12	AKAP12
ENSG00000265205	3.12	
ENSG00000162078	3.12	ZG16B
ENSG00000163904	3.12	SENP2
ENSG00000072858	3.12	SIDT1

ENSG00000107669	3.12	ATE1
ENSG00000137941	3.12	TTLL7
ENSG00000143536	3.12	CRNN
ENSG00000197208	3.12	SLC22A4
ENSG00000224431	3.12	
ENSG00000115520	3.12	COQ10B
ENSG00000104221	3.12	BRF2
ENSG00000171786	3.13	NHLH1
ENSG00000259756	3.13	
ENSG00000275111	3.13	ZNF2
ENSG00000281357	3.13	ARRDC3-AS1
ENSG00000249456	3.13	
ENSG00000157350	3.13	ST3GAL2
ENSG00000250596	3.13	
ENSG00000131779	3.14	PEX11B
ENSG00000177191	3.14	B3GNT8
ENSG00000230064	3.14	
ENSG00000174721	3.14	FGFBP3
ENSG00000187054	3.14	TMPRSS11A
ENSG00000188536	3.14	HBA2
ENSG00000213028	3.14	
ENSG00000214870	3.14	
ENSG00000224888	3.14	
ENSG00000162542	3.14	TMCO4
ENSG00000243305	3.14	
ENSG00000140525	3.14	FANCI
ENSG00000115808	3.14	STRN
ENSG00000256591	3.14	
ENSG00000170089	3.15	LOC728554
ENSG00000006042	3.15	TMEM98
ENSG00000254204	3.15	
ENSG00000122557	3.15	HERPUD2
ENSG00000204219	3.15	TCEA3
ENSG00000232490	3.15	OSBPL10-AS1
ENSG00000287525	3.15	
ENSG00000186908	3.15	ZDHHC17
ENSG00000153029	3.15	MR1
ENSG00000042062	3.16	RIPOR3
ENSG00000104879	3.16	CKM
ENSG00000196126	3.16	HLA-DRB1
ENSG00000205898	3.16	
ENSG00000224272	3.16	

ENSG00000236069	3.16	
ENSG00000183111	3.16	ARHGEF37
ENSG00000214106	3.17	PAXIP1-AS2
ENSG00000212952	3.17	
ENSG00000138399	3.17	FASTKD1
ENSG00000171649	3.18	ZIK1
ENSG00000237353	3.18	PATE4
ENSG00000242798	3.18	
ENSG00000248554	3.18	
ENSG00000134262	3.18	AP4B1
ENSG00000238198	3.18	LOC100996251
ENSG00000108468	3.18	CBX1
ENSG00000074211	3.19	PPP2R2C
ENSG00000279672	3.19	
ENSG00000272138	3.19	LINC01607
ENSG00000140931	3.20	CMTM3
ENSG00000178163	3.20	ZNF518B
ENSG00000047410	3.20	TPR
ENSG00000122786	3.20	CALD1
ENSG00000106526	3.20	ACTR3C
ENSG00000167601	3.20	AXL
ENSG00000215455	3.20	KRTAP10-1
ENSG00000047621	3.21	C12orf4
ENSG00000177943	3.21	MAMDC4
ENSG00000278987	3.21	
ENSG00000283317	3.21	
ENSG00000205763	3.21	RP9P
ENSG00000288683	3.21	
ENSG00000066777	3.22	ARFGEF1
ENSG00000264743	3.22	DPRXP4
ENSG00000284391	3.22	
ENSG00000147416	3.23	ATP6V1B2
ENSG00000111339	3.23	ART4
ENSG00000235189	3.23	
ENSG00000241058	3.23	NSUN6
ENSG00000254708	3.23	
ENSG00000269106	3.23	
ENSG00000105880	3.23	DLX5
ENSG00000160678	3.24	S100A1
ENSG00000236991	3.24	EDRF1-AS1
ENSG00000095777	3.24	MYO3A
ENSG00000220773	3.24	

ENSG00000258711	3.24	
ENSG00000172766	3.24	NAA16
ENSG00000260618	3.25	
ENSG00000130054	3.25	FAM155B
ENSG00000266844	3.25	
ENSG00000153531	3.25	ADPRHL1
ENSG00000136488	3.25	CSH1
ENSG00000188921	3.25	HACD4
ENSG00000246560	3.25	LOC105377348
ENSG00000273002	3.25	
ENSG00000269907	3.25	
ENSG00000233321	3.26	
ENSG00000125409	3.26	TEKT3
ENSG00000260000	3.26	
ENSG00000274553	3.26	
ENSG00000152642	3.26	GPD1L
ENSG00000284735	3.26	
ENSG00000198754	3.27	OXCT2
ENSG00000234607	3.27	
ENSG00000244731	3.27	C4A
ENSG00000151498	3.27	ACAD8
ENSG00000140932	3.28	CMTM2
ENSG00000258924	3.28	
ENSG00000224940	3.28	PRRT4
ENSG00000229154	3.28	KCNQ5-AS1
ENSG00000229657	3.28	
ENSG00000249407	3.28	
ENSG00000272831	3.28	
ENSG00000144559	3.29	TAMM41
ENSG00000268061	3.29	NAPA-AS1
ENSG00000033327	3.29	GAB2
ENSG00000166920	3.29	C15orf48
ENSG00000198093	3.29	ZNF649
ENSG00000267279	3.29	
ENSG00000171960	3.30	PPIH
ENSG00000157554	3.30	ERG
ENSG00000174028	3.30	
ENSG00000226533	3.30	
ENSG00000272842	3.30	
ENSG00000142687	3.30	KIAA0319L
ENSG00000135127	3.30	BICDL1
ENSG00000251022	3.30	THAP9-AS1

ENSG00000047230	3.30	CTPS2
ENSG00000121380	3.31	BCL2L14
ENSG00000120942	3.31	UBIAD1
ENSG00000154124	3.31	OTULIN
ENSG00000157303	3.32	SUSD3
ENSG00000167524	3.32	SGK494
ENSG00000261408	3.32	TEN1-CDK3
ENSG00000268366	3.32	
ENSG00000206075	3.32	SERPINB5
ENSG00000176170	3.32	SPHK1
ENSG00000257261	3.33	
ENSG00000175581	3.33	MRPL48
ENSG00000068971	3.33	PPP2R5B
ENSG00000082269	3.33	FAM135A
ENSG00000185467	3.33	KPNA7
ENSG00000227954	3.33	TARID
ENSG00000254995	3.33	STX16-NPEPL1
ENSG00000264668	3.33	
ENSG00000228925	3.33	
ENSG00000147044	3.34	CASK
ENSG00000187186	3.34	LOC730098
ENSG00000253536	3.34	
ENSG00000261633	3.34	
ENSG00000175606	3.35	TMEM70
ENSG00000071243	3.35	ING3
ENSG00000236452	3.35	
ENSG00000237783	3.35	
ENSG00000260650	3.35	
ENSG00000258559	3.35	
ENSG00000244879	3.36	
ENSG00000116497	3.36	S100PBP
ENSG00000198042	3.36	MAK16
ENSG00000165948	3.37	IFI27L1
ENSG00000235024	3.37	
ENSG00000100968	3.37	NFATC4
ENSG00000089820	3.38	ARHGAP4
ENSG00000152684	3.38	PELO
ENSG00000150625	3.38	GPM6A
ENSG00000197019	3.38	SERTAD1
ENSG00000232104	3.38	RFX3-AS1
ENSG00000243095	3.38	
ENSG00000155324	3.38	GRAMD2B

ENSG00000145293	3.38	ENOPH1
ENSG00000205436	3.38	EXOC3L4
ENSG00000179715	3.39	PCED1B
ENSG00000236383	3.39	LINC00854
ENSG00000163694	3.39	RBM47
ENSG00000285820	3.39	
ENSG00000118939	3.39	UCHL3
ENSG00000133424	3.39	LARGE1
ENSG00000151276	3.39	MAGI1
ENSG00000230487	3.40	PSMG3-AS1
ENSG00000197416	3.40	FABP12
ENSG00000230606	3.40	
ENSG00000272202	3.40	
ENSG00000108839	3.41	ALOX12
ENSG00000170298	3.41	LGALS9B
ENSG00000099999	3.41	RNF215
ENSG00000034677	3.41	RNF19A
ENSG00000197417	3.41	
ENSG00000255471	3.41	
ENSG00000273340	3.41	
ENSG00000264859	3.42	DSG2-AS1
ENSG00000019169	3.42	MARCO
ENSG00000215158	3.42	
ENSG00000260621	3.42	
ENSG00000150054	3.42	MPP7
ENSG00000205710	3.43	C17orf107
ENSG00000248844	3.43	LOC101928443
ENSG00000277287	3.43	
ENSG00000174428	3.43	GTF2IRD2B
ENSG00000065923	3.44	SLC9A7
ENSG00000198937	3.44	CCDC167
ENSG00000169084	3.44	DHRSX
ENSG00000181826	3.44	RELL1
ENSG00000182511	3.44	FES
ENSG00000224551	3.44	
ENSG00000267313	3.44	KC6
ENSG00000164002	3.44	EXO5
ENSG00000151779	3.45	NBAS
ENSG00000102003	3.45	SYP
ENSG00000172974	3.45	
ENSG00000242154	3.45	
ENSG00000267904	3.45	

ENSG00000117971	3.46	CHRNB4
ENSG00000049323	3.46	LTBP1
ENSG00000144485	3.47	HES6
ENSG00000186704	3.47	
ENSG00000249082	3.47	C5orf66-AS1
ENSG00000116701	3.47	NCF2
ENSG00000250536	3.48	
ENSG00000253931	3.48	
ENSG00000281379	3.48	
ENSG00000286629	3.48	
ENSG00000169314	3.48	C22orf15
ENSG00000124171	3.49	PARD6B
ENSG00000231728	3.49	
ENSG00000272037	3.49	
ENSG00000240344	3.49	PPIL3
ENSG00000133401	3.50	PDZD2
ENSG00000205622	3.50	
ENSG00000099866	3.50	MADCAM1
ENSG00000185986	3.50	
ENSG00000189127	3.50	ANKRD34B
ENSG00000205307	3.50	SAP25
ENSG00000232832	3.50	LMLN-AS1
ENSG00000287049	3.50	
ENSG00000251365	3.51	
ENSG00000178752	3.51	ERFE
ENSG00000263345	3.51	
ENSG00000188525	3.52	
ENSG00000204352	3.52	C9orf129
ENSG00000250539	3.52	
ENSG00000257809	3.52	
ENSG00000286659	3.52	
ENSG00000011478	3.52	QPCTL
ENSG00000099246	3.52	RAB18
ENSG00000286672	3.52	
ENSG00000155719	3.53	OTOA
ENSG00000226772	3.53	
ENSG00000235314	3.53	
ENSG00000247400	3.53	DNAJC3-AS1
ENSG00000281912	3.53	LINC01144
ENSG00000134152	3.54	KATNBL1
ENSG00000170684	3.54	ZNF296
ENSG00000171989	3.54	LDHAL6B

ENSG00000180610	3.54	
ENSG00000259033	3.54	
ENSG00000204406	3.54	MBD5
ENSG00000247828	3.55	TMEM161B-AS1
ENSG00000287966	3.55	
ENSG00000225830	3.55	PGBD3
ENSG00000080371	3.55	RAB21
ENSG00000283769	3.55	
ENSG00000134970	3.55	TMED7
ENSG00000155974	3.55	GRIP1
ENSG00000183313	3.55	OR52L1
ENSG00000187522	3.55	HSPA14
ENSG00000005810	3.55	MYCBP2
ENSG00000177519	3.55	RPRM
ENSG00000176198	3.56	OR11H4
ENSG00000259315	3.56	
ENSG00000275371	3.56	
ENSG00000261071	3.56	
ENSG00000287216	3.56	
ENSG00000174173	3.57	TRMT10C
ENSG00000262412	3.57	
ENSG00000143479	3.57	DYRK3
ENSG00000224389	3.57	C4B
ENSG00000226644	3.57	
ENSG00000234911	3.57	
ENSG00000256862	3.58	
ENSG00000249558	3.58	
ENSG00000225146	3.58	
ENSG00000254566	3.58	
ENSG00000280357	3.58	
ENSG00000286104	3.58	
ENSG00000287678	3.58	
ENSG00000231991	3.58	
ENSG00000184321	3.58	
ENSG00000287829	3.58	
ENSG00000287808	3.58	
ENSG00000019485	3.58	PRDM11
ENSG00000196183	3.58	
ENSG00000254317	3.59	
ENSG00000124370	3.59	MCEE
ENSG00000287837	3.59	
ENSG00000060762	3.59	MPC1

ENSG00000102984	3.60	ZNF821
ENSG00000240666	3.60	
ENSG00000125149	3.60	C16orf70
ENSG00000138018	3.60	SELENOI
ENSG00000006025	3.61	OSBPL7
ENSG00000231086	3.61	
ENSG00000223353	3.61	
ENSG00000068831	3.61	RASGRP2
ENSG00000181097	3.61	
ENSG00000236064	3.61	LOC101928335
ENSG00000262003	3.61	LOC101927727
ENSG00000170502	3.62	NUDT9
ENSG00000279758	3.62	
ENSG00000235257	3.62	ITGA9-AS1
ENSG00000180263	3.62	FGD6
ENSG00000278601	3.63	
ENSG00000237846	3.63	
ENSG00000272588	3.63	
ENSG00000101278	3.63	
ENSG00000214278	3.63	
ENSG00000273245	3.63	
ENSG00000118777	3.63	ABCG2
ENSG00000162601	3.64	MYSM1
ENSG00000277984	3.64	
ENSG00000076356	3.64	PLXNA2
ENSG00000287346	3.64	
ENSG00000224107	3.64	ETDB
ENSG00000238210	3.64	
ENSG00000249947	3.64	
ENSG00000237719	3.64	
ENSG00000119636	3.64	BBOF1
ENSG00000183397	3.65	C19orf71
ENSG00000174442	3.65	ZWILCH
ENSG00000272087	3.65	
ENSG00000126773	3.66	PCNX4
ENSG00000105479	3.66	CCDC114
ENSG00000246582	3.66	LOC389641
ENSG00000156413	3.66	FUT6
ENSG00000229065	3.66	
ENSG00000132000	3.66	PODNL1
ENSG00000277501	3.66	
ENSG00000230201	3.66	

ENSG00000237094	3.66	
ENSG00000137500	3.66	CCDC90B
ENSG00000101417	3.67	PXMP4
ENSG00000100167	3.67	SEPT3
ENSG00000104691	3.67	UBXN8
ENSG00000226608	3.67	
ENSG00000267394	3.67	
ENSG00000278133	3.67	
ENSG00000279262	3.67	
ENSG00000254397	3.67	
ENSG00000172954	3.68	LCLAT1
ENSG00000254230	3.68	
ENSG00000271550	3.68	
ENSG00000101440	3.69	ASIP
ENSG00000244485	3.69	
ENSG00000142233	3.69	NTN5
ENSG00000272821	3.69	
ENSG00000159079	3.69	C21orf59
ENSG00000148950	3.69	IMMP1L
ENSG00000251192	3.69	ZNF674
ENSG00000147535	3.69	PLPP5
ENSG00000258344	3.69	
ENSG00000233445	3.70	
ENSG00000177669	3.70	MBOAT4
ENSG00000235944	3.70	
ENSG00000271754	3.70	
ENSG00000223583	3.71	
ENSG00000236165	3.71	
ENSG00000141664	3.71	ZCCHC2
ENSG00000183092	3.72	BEGAIN
ENSG00000180398	3.72	MCFD2
ENSG00000119599	3.72	DCAF4
ENSG00000108515	3.72	ENO3
ENSG00000258425	3.72	
ENSG00000170364	3.72	SETMAR
ENSG00000237972	3.72	
ENSG00000170627	3.72	GTSF1
ENSG00000168148	3.72	HIST3H3
ENSG00000257121	3.73	
ENSG00000241634	3.73	
ENSG00000196569	3.73	LAMA2
ENSG00000138686	3.73	BBS7

ENSG00000169902	3.73	TPST1
ENSG00000132294	3.73	EFR3A
ENSG00000264920	3.75	LOC102724532
ENSG00000286896	3.75	
ENSG00000104381	3.76	GDAP1
ENSG00000150175	3.76	
ENSG00000154545	3.76	MAGED4
ENSG00000256340	3.76	ABCC6P1
ENSG00000182676	3.76	PPP1R27
ENSG00000188725	3.76	SMIM15
ENSG00000169403	3.77	PTAFR
ENSG00000273184	3.77	
ENSG00000121741	3.77	ZMYM2
ENSG00000116260	3.78	QSOX1
ENSG00000100379	3.78	KCTD17
ENSG00000167378	3.78	IRGQ
ENSG00000105374	3.78	NKG7
ENSG00000134198	3.78	TSPAN2
ENSG00000141854	3.78	MISP3
ENSG00000227398	3.78	
ENSG00000231312	3.78	LOC728730
ENSG00000258527	3.78	
ENSG00000108187	3.78	PBLD
ENSG00000187266	3.78	EPOR
ENSG00000206203	3.78	TSSK2
ENSG00000258623	3.79	
ENSG00000279799	3.79	
ENSG00000232956	3.79	SNHG15
ENSG00000236552	3.80	RPL13AP5
ENSG00000184206	3.80	GOLGA6L4
ENSG00000224892	3.80	
ENSG00000150455	3.80	TIRAP
ENSG00000155903	3.80	RASA2
ENSG00000129355	3.81	CDKN2D
ENSG00000259332	3.81	ST20-MTHFS
ENSG00000249240	3.81	
ENSG00000180318	3.81	ALX1
ENSG00000226674	3.81	TEX41
ENSG00000275674	3.81	
ENSG00000281383	3.81	
ENSG00000111911	3.81	HINT3
ENSG00000234203	3.82	

ENSG00000197568	3.82	HHLA3
ENSG00000163877	3.82	SNIP1
ENSG00000227232	3.82	
ENSG00000264070	3.82	
ENSG00000168995	3.82	SIGLEC7
ENSG00000132744	3.83	ACY3
ENSG00000033627	3.83	ATP6V0A1
ENSG00000119917	3.83	IFIT3
ENSG00000219395	3.83	
ENSG00000254578	3.83	
ENSG00000253706	3.84	
ENSG00000264666	3.84	
ENSG00000142185	3.84	TRPM2
ENSG00000144228	3.85	SPOPL
ENSG00000229848	3.85	
ENSG00000270948	3.85	
ENSG00000250320	3.85	
ENSG00000143033	3.86	MTF2
ENSG00000120008	3.87	WDR11
ENSG00000115539	3.87	PDCL3
ENSG00000111110	3.87	PPM1H
ENSG00000131233	3.87	GJA9
ENSG00000159871	3.88	LYPD5
ENSG00000112200	3.88	ZNF451
ENSG00000220925	3.88	
ENSG00000188785	3.88	ZNF548
ENSG00000185710	3.91	
ENSG00000279713	3.91	
ENSG00000286522	3.91	
ENSG00000136856	3.91	SLC2A8
ENSG00000163632	3.92	C3orf49
ENSG00000204882	3.92	GPR20
ENSG00000232197	3.92	
ENSG00000255446	3.92	
ENSG00000286119	3.92	
ENSG00000279071	3.92	
ENSG00000157796	3.92	WDR19
ENSG00000214309	3.92	MBLAC1
ENSG00000270804	3.93	
ENSG00000204152	3.93	TIMM23B
ENSG00000112394	3.93	SLC16A10
ENSG00000107819	3.94	SFXN3

ENSG00000213085	3.94	CFAP45
ENSG00000258524	3.94	
ENSG00000288598	3.94	
ENSG00000260128	3.95	ULK4P2
ENSG00000222014	3.95	RAB6C
ENSG00000162670	3.95	BRINP3
ENSG00000236519	3.95	
ENSG00000139193	3.95	CD27
ENSG00000134321	3.96	RSAD2
ENSG00000234709	3.96	
ENSG00000236670	3.96	
ENSG00000178363	3.97	CALML3
ENSG00000216921	3.97	LOC285095
ENSG00000258279	3.97	LINC00592
ENSG00000279827	3.97	
ENSG00000287329	3.97	
ENSG00000137876	3.97	RSL24D1
ENSG00000277203	3.98	F8A1
ENSG00000272870	3.98	
ENSG00000138111	3.98	MFSD13A
ENSG00000139133	3.99	ALG10
ENSG00000177311	3.99	ZBTB38
ENSG00000183054	3.99	RGPD6
ENSG00000279747	3.99	
ENSG00000177300	3.99	CLDN22
ENSG00000170540	4.00	ARL6IP1
ENSG00000214700	4.00	C12orf71
ENSG00000226471	4.00	
ENSG00000250746	4.00	
ENSG00000255538	4.00	OR10V2P
ENSG00000258985	4.00	
ENSG00000203760	4.01	CENPW
ENSG00000198680	4.01	TUSC1
ENSG00000135070	4.02	ISCA1
ENSG00000171695	4.02	LKAAEAR1
ENSG00000234111	4.02	LINC02433
ENSG00000261124	4.02	
ENSG00000262663	4.02	
ENSG00000169372	4.02	CRADD
ENSG00000243302	4.03	
ENSG00000265218	4.03	
ENSG00000235138	4.03	LOC100130548

ENSG00000188786	4.03	MTF1
ENSG00000137628	4.04	DDX60
ENSG00000154743	4.04	TSEN2
ENSG00000187796	4.05	CARD9
ENSG00000136273	4.05	HUS1
ENSG00000185888	4.05	PRSS38
ENSG00000198520	4.05	C1orf228
ENSG00000214575	4.05	CPEB1
ENSG00000282358	4.05	
ENSG00000176909	4.06	MAMSTR
ENSG00000086200	4.06	IPO11-LRRC70
ENSG00000184106	4.06	TREML3P
ENSG00000272910	4.06	
ENSG00000287196	4.06	
ENSG00000175170	4.07	
ENSG00000217653	4.07	
ENSG00000231443	4.08	
ENSG00000274211	4.08	SOCS7
ENSG00000115297	4.08	TLX2
ENSG00000163273	4.08	NPPC
ENSG00000104231	4.08	ZFAND1
ENSG00000230993	4.09	
ENSG00000233469	4.09	
ENSG00000143001	4.10	TMEM61
ENSG00000230943	4.10	LOC101927686
ENSG00000178401	4.11	DNAJC22
ENSG00000183454	4.11	GRIN2A
ENSG00000217130	4.11	
ENSG00000276533	4.11	
ENSG00000198466	4.11	ZNF587
ENSG00000185527	4.12	PDE6G
ENSG00000267649	4.12	
ENSG00000255143	4.13	
ENSG00000245317	4.13	LOC100996419
ENSG00000232320	4.13	
ENSG00000164532	4.14	TBX20
ENSG00000166454	4.14	ATMIN
ENSG00000275221	4.14	HIST1H2AK
ENSG00000276842	4.14	
ENSG00000157869	4.14	RAB28
ENSG00000256576	4.15	LINC02361
ENSG00000214783	4.16	POLR2J4

ENSG00000272072	4.17	
ENSG00000172938	4.17	MRGPRD
ENSG00000198064	4.18	NPIPB13
ENSG00000242735	4.18	
ENSG00000271870	4.18	
ENSG00000277775	4.18	HIST1H3F
ENSG00000285881	4.18	
ENSG00000139719	4.18	
ENSG00000188706	4.19	ZDHHC9
ENSG00000227638	4.19	
ENSG00000218069	4.19	
ENSG00000230061	4.19	
ENSG00000249085	4.19	
ENSG00000147133	4.19	TAF1
ENSG00000175582	4.19	RAB6A
ENSG00000188010	4.20	MORN2
ENSG00000231680	4.20	
ENSG00000100523	4.21	DDHD1
ENSG00000171116	4.21	HSFX1
ENSG00000198814	4.21	GK
ENSG00000244945	4.21	LOC101928445
ENSG00000238035	4.22	
ENSG00000196581	4.22	AJAP1
ENSG00000244476	4.22	ERVFRD-1
ENSG00000256040	4.22	PAPPA-AS1
ENSG00000152219	4.23	ARL14EP
ENSG00000275180	4.23	
ENSG00000243243	4.24	
ENSG00000131931	4.24	THAP1
ENSG00000134490	4.24	TMEM241
ENSG00000013375	4.24	PGM3
ENSG00000038295	4.24	TLL1
ENSG00000180953	4.25	ST20
ENSG00000228205	4.26	
ENSG00000277491	4.26	
ENSG00000259712	4.26	
ENSG00000204382	4.27	XAGE1E
ENSG00000259496	4.27	
ENSG00000180329	4.27	CCDC43
ENSG00000258724	4.28	
ENSG00000111653	4.28	ING4
ENSG00000253784	4.28	

ENSG00000262769	4.28	
ENSG00000054392	4.28	HHAT
ENSG00000173436	4.29	
ENSG00000136834	4.29	OR1J1
ENSG00000226476	4.29	
ENSG00000255441	4.29	
ENSG00000166670	4.29	MMP10
ENSG00000155592	4.30	ZKSCAN2
ENSG00000147586	4.30	MRPS28
ENSG00000213592	4.30	
ENSG00000253841	4.30	
ENSG00000279021	4.30	
ENSG00000172716	4.30	SLFN11
ENSG00000223396	4.31	
ENSG00000249129	4.31	
ENSG00000188373	4.32	C10orf99
ENSG00000288573	4.32	
ENSG00000251066	4.32	
ENSG00000155959	4.32	VBP1
ENSG00000162997	4.34	PRORSD1P
ENSG00000285803	4.35	
ENSG00000225693	4.35	
ENSG00000257253	4.35	
ENSG00000138614	4.35	INTS14
ENSG00000168056	4.35	LTBP3
ENSG00000174306	4.36	ZHX3
ENSG00000224831	4.36	
ENSG00000145781	4.36	COMMD10
ENSG00000267644	4.36	
ENSG00000230532	4.37	
ENSG00000255750	4.37	
ENSG00000138080	4.38	EMILIN1
ENSG00000162755	4.38	KLHDC9
ENSG00000117543	4.38	DPH5
ENSG00000067208	4.39	EVI5
ENSG00000170903	4.39	MSANTD4
ENSG00000254413	4.39	CHKB-CPT1B
ENSG00000188801	4.39	
ENSG00000217643	4.39	
ENSG00000230615	4.39	
ENSG00000258789	4.39	
ENSG00000273361	4.39	

ENSG00000134575	4.39	ACP2
ENSG00000280054	4.40	
ENSG00000178440	4.40	
ENSG00000162241	4.41	SLC25A45
ENSG00000115041	4.42	KCNIP3
ENSG00000182057	4.43	OGFRP1
ENSG00000126391	4.43	FRMD8
ENSG00000259692	4.43	
ENSG00000174326	4.44	SLC16A11
ENSG00000147697	4.44	GSDMC
ENSG00000118246	4.44	FASTKD2
ENSG00000243926	4.45	
ENSG00000240828	4.45	
ENSG00000154240	4.46	CEP112
ENSG00000248429	4.46	
ENSG00000197110	4.47	IFNL3
ENSG00000250155	4.47	
ENSG00000266717	4.47	
ENSG00000124143	4.49	ARHGAP40
ENSG00000272844	4.49	
ENSG00000145911	4.50	N4BP3
ENSG00000213663	4.50	
ENSG00000223829	4.50	
ENSG00000225511	4.50	
ENSG00000261884	4.51	
ENSG00000089163	4.52	SIRT4
ENSG00000235308	4.52	
ENSG00000261879	4.53	LOC100130950
ENSG00000260448	4.54	
ENSG00000113742	4.55	CPEB4
ENSG00000122008	4.55	POLK
ENSG00000260693	4.55	
ENSG00000285766	4.55	
ENSG00000121988	4.56	ZRANB3
ENSG00000168746	4.56	LINC01430
ENSG00000176884	4.56	GRIN1
ENSG00000268173	4.56	
ENSG00000271868	4.56	
ENSG00000164329	4.56	PAPD4
ENSG00000240184	4.57	
ENSG00000278399	4.57	
ENSG00000082213	4.57	C5orf22

ENSG00000166002	4.58	SMCO4
ENSG00000246339	4.58	EXTL3-AS1
ENSG00000241635	4.59	UGT1A1
ENSG00000144136	4.59	SLC20A1
ENSG00000109929	4.60	SC5D
ENSG00000149231	4.60	CCDC82
ENSG00000172382	4.60	PRSS27
ENSG00000174448	4.60	STARD6
ENSG00000197056	4.60	ZMYM1
ENSG00000220749	4.61	RPL21P28
ENSG00000157551	4.61	KCNJ15
ENSG00000197779	4.62	ZNF81
ENSG00000198586	4.63	TLK1
ENSG00000100060	4.63	MFNG
ENSG00000178233	4.63	TMEM151B
ENSG00000229048	4.63	
ENSG00000230798	4.63	FOXD3-AS1
ENSG00000167566	4.64	NCKAP5L
ENSG00000154079	4.64	SDHAF4
ENSG00000036448	4.64	MYOM2
ENSG00000133703	4.64	KRAS
ENSG00000236478	4.64	
ENSG00000186792	4.64	HYAL3
ENSG00000230562	4.65	
ENSG00000260033	4.65	
ENSG00000267751	4.65	LOC105372233
ENSG00000109756	4.66	RAPGEF2
ENSG00000182950	4.67	ODF3L1
ENSG00000273289	4.67	
ENSG00000130349	4.67	C6orf203
ENSG00000163738	4.67	MTHFD2L
ENSG00000240087	4.68	
ENSG00000162552	4.68	WNT4
ENSG00000134363	4.68	FST
ENSG00000189136	4.68	UBE2Q2P1
ENSG00000257800	4.68	
ENSG00000278112	4.69	
ENSG00000240051	4.71	
ENSG00000278383	4.71	
ENSG00000134258	4.72	VTCN1
ENSG00000136114	4.72	THSD1
ENSG00000227959	4.72	

ENSG00000270181	4.72	BIVM-ERCC5
ENSG00000136048	4.73	DRAM1
ENSG00000165972	4.73	CCDC38
ENSG00000225928	4.73	
ENSG00000124279	4.74	FASTKD3
ENSG00000128731	4.74	HERC2
ENSG00000115970	4.75	THADA
ENSG00000112742	4.75	TTK
ENSG00000229017	4.75	
ENSG00000258955	4.75	
ENSG00000267741	4.75	
ENSG00000236051	4.75	MYCBP2-AS1
ENSG00000189343	4.75	
ENSG00000128652	4.76	HOXD3
ENSG00000224712	4.76	NPIPA3
ENSG00000226669	4.78	
ENSG00000270419	4.78	CAHM
ENSG00000070761	4.78	CFAP20
ENSG00000111647	4.79	UHRF1BP1L
ENSG00000213658	4.79	LAT
ENSG00000224637	4.79	
ENSG00000251229	4.80	
ENSG00000151640	4.80	DPYSL4
ENSG00000255200	4.80	
ENSG00000258748	4.80	
ENSG00000286242	4.81	
ENSG00000105696	4.81	TMEM59L
ENSG00000006652	4.81	IFRD1
ENSG00000269153	4.82	
ENSG00000238110	4.82	
ENSG00000235280	4.83	
ENSG00000214295	4.84	
ENSG00000258412	4.84	
ENSG00000257732	4.84	
ENSG00000205236	4.84	
ENSG00000273011	4.84	
ENSG00000250151	4.85	ARPC4-TTLL3
ENSG00000229591	4.85	
ENSG00000284634	4.85	
ENSG00000179776	4.86	CDH5
ENSG00000263335	4.86	
ENSG00000270332	4.86	SMC2-AS1

ENSG00000272092	4.86	
ENSG00000142227	4.87	EMP3
ENSG00000089818	4.88	NECAP1
ENSG00000144468	4.88	RHBDD1
ENSG00000214322	4.89	
ENSG00000251669	4.90	FAM86EP
ENSG00000115461	4.91	IGFBP5
ENSG00000262655	4.92	SPON1
ENSG00000253121	4.92	
ENSG00000240859	4.93	
ENSG00000262227	4.93	
ENSG00000161509	4.94	GRIN2C
ENSG00000249679	4.94	LOC105377590
ENSG00000251520	4.94	
ENSG00000269388	4.95	
ENSG00000261469	4.95	
ENSG00000171054	4.96	OR13H1
ENSG00000223783	4.96	
ENSG00000204625	4.97	HCG9
ENSG00000236594	4.98	
ENSG00000258458	4.98	
ENSG00000272777	4.98	
ENSG00000135040	4.99	NAA35
ENSG00000214243	4.99	
ENSG00000165695	4.99	AK8
ENSG00000262480	4.99	
ENSG00000058729	4.99	RIOK2
ENSG00000157470	5.00	FAM81A
ENSG00000271983	5.00	
ENSG00000227492	5.00	
ENSG00000130023	5.01	ERMARD
ENSG00000277117	5.02	LOC102723996
ENSG00000269038	5.03	
ENSG00000157570	5.04	TSPAN18
ENSG00000153898	5.04	MCOLN2
ENSG00000258769	5.05	
ENSG00000161149	5.06	TUBA3FP
ENSG00000260439	5.06	LMF1-AS1
ENSG00000067715	5.06	SYT1
ENSG00000206538	5.07	VGLL3
ENSG00000176809	5.07	LRRC37A3
ENSG00000116667	5.07	C1orf21

ENSG00000239920	5.08	
ENSG00000283402	5.08	
ENSG00000172650	5.08	AGAP5
ENSG00000080031	5.09	PTPRH
ENSG00000172943	5.09	PHF8
ENSG00000280916	5.10	
ENSG00000115947	5.10	ORC4
ENSG00000165794	5.10	SLC39A2
ENSG00000279594	5.11	
ENSG00000168993	5.11	CPLX1
ENSG00000243055	5.11	
ENSG00000273680	5.11	
ENSG00000104671	5.12	DCTN6
ENSG00000176209	5.13	SMIM19
ENSG00000163209	5.13	SPRR3
ENSG00000258984	5.13	UBE2F-SCLY
ENSG00000267005	5.13	
ENSG00000241404	5.13	EGFL8
ENSG00000272702	5.13	
ENSG00000256940	5.14	LOC105369340
ENSG00000228823	5.14	
ENSG00000172548	5.15	NIPAL4
ENSG00000173080	5.15	RXFP4
ENSG00000255202	5.15	LOC105376617
ENSG00000269949	5.15	
ENSG00000272444	5.15	
ENSG00000180861	5.16	LINC01559
ENSG00000180089	5.17	TMEM86B
ENSG00000147121	5.18	KRBOX4
ENSG00000259125	5.19	LRP1-AS
ENSG00000284484	5.20	
ENSG00000169087	5.21	HSPBAP1
ENSG00000244219	5.21	TMEM225B
ENSG00000254779	5.21	
ENSG00000267372	5.21	
ENSG00000177054	5.22	ZDHHC13
ENSG00000272100	5.23	
ENSG00000253550	5.23	
ENSG00000286031	5.24	
ENSG00000166261	5.25	ZNF202
ENSG00000160766	5.26	GBAP1
ENSG00000262050	5.26	LOC105371592

ENSG00000272158	5.26	
ENSG00000250564	5.27	
ENSG00000165030	5.28	NFIL3
ENSG00000108797	5.28	CNTNAP1
ENSG00000179141	5.28	
ENSG00000135931	5.29	ARMC9
ENSG00000132832	5.30	
ENSG00000284644	5.31	
ENSG00000137573	5.31	SULF1
ENSG00000240463	5.31	
ENSG00000153234	5.31	NR4A2
ENSG00000120458	5.32	MSANTD2
ENSG00000111554	5.33	MDM1
ENSG00000164049	5.33	FBXW12
ENSG00000173727	5.33	
ENSG00000231969	5.33	LOC101929231
ENSG00000251112	5.36	
ENSG00000259540	5.36	LOC102723335
ENSG00000162614	5.37	NEXN
ENSG00000273455	5.37	
ENSG00000232386	5.37	
ENSG00000143847	5.38	PPFIA4
ENSG00000280852	5.39	LOC653653
ENSG00000286532	5.39	
ENSG00000218336	5.40	TENM3
ENSG00000262668	5.41	
ENSG00000185808	5.43	PIGP
ENSG00000169762	5.43	TAPT1
ENSG00000133067	5.44	LGR6
ENSG00000226756	5.44	
ENSG00000274213	5.44	
ENSG00000184111	5.45	
ENSG00000235245	5.45	
ENSG00000258378	5.45	
ENSG00000168824	5.46	NSG1
ENSG00000100815	5.46	TRIP11
ENSG00000144395	5.47	CCDC150
ENSG00000134769	5.48	DTNA
ENSG00000143178	5.48	TBX19
ENSG00000187556	5.48	NANOS3
ENSG00000112249	5.50	ASCC3
ENSG00000223701	5.51	

ENSG00000231748	5.51	
ENSG00000166128	5.53	RAB8B
ENSG00000205426	5.53	KRT81
ENSG00000260992	5.53	
ENSG00000114735	5.53	HEMK1
ENSG00000286588	5.54	
ENSG00000185966	5.55	LCE3E
ENSG00000171428	5.56	NAT1
ENSG00000274372	5.56	
ENSG00000172425	5.59	TTC36
ENSG00000231691	5.60	
ENSG00000163686	5.60	ABHD6
ENSG00000124802	5.61	EEF1E1
ENSG00000237940	5.63	LINC01238
ENSG00000250282	5.63	
ENSG00000267057	5.63	
ENSG00000139793	5.63	MBNL2
ENSG00000213791	5.64	
ENSG00000225489	5.65	
ENSG00000135469	5.66	COQ10A
ENSG00000158301	5.66	GPRASP2
ENSG00000054179	5.66	ENTPD2
ENSG00000250318	5.66	
ENSG00000229891	5.66	LINC01315
ENSG00000260727	5.69	
ENSG00000100312	5.69	ACR
ENSG00000185664	5.70	PMEL
ENSG00000182810	5.70	DDX28
ENSG00000114698	5.70	PLSCR4
ENSG00000188177	5.70	ZC3H6
ENSG00000233902	5.70	
ENSG00000011422	5.72	PLAUR
ENSG00000271200	5.72	
ENSG00000139324	5.73	TMTC3
ENSG00000254653	5.77	
ENSG00000227056	5.78	
ENSG00000152413	5.78	HOMER1
ENSG00000087502	5.80	ERGIC2
ENSG00000150756	5.80	FAM173B
ENSG00000264272	5.80	
ENSG00000204540	5.81	PSORS1C1
ENSG00000204934	5.82	ATP6V0E2-AS1

ENSG00000173846	5.83	PLK3
ENSG00000039987	5.84	BEST2
ENSG00000282306	5.84	
ENSG00000272142	5.85	LYRM4-AS1
ENSG00000260193	5.87	
ENSG00000283251	5.87	
ENSG00000240891	5.88	PLCXD2
ENSG00000282300	5.88	
ENSG00000274259	5.89	
ENSG00000285812	5.91	
ENSG00000282339	5.91	
ENSG00000214194	5.92	LINC00998
ENSG00000239706	5.92	
ENSG00000273450	5.92	
ENSG00000163626	5.93	COX18
ENSG00000287384	5.93	
ENSG00000278921	5.94	EPB41L4A-AS2
ENSG00000163467	5.94	TSACC
ENSG00000196344	5.95	ADH7
ENSG00000126860	5.95	EVI2A
ENSG00000170464	5.96	DNAJC18
ENSG00000172671	5.96	ZFAND4
ENSG00000182870	5.97	GALNT9
ENSG00000203711	5.97	
ENSG00000198576	5.97	ARC
ENSG00000282787	5.97	
ENSG00000286231	5.97	
ENSG00000229967	6.01	
ENSG00000177335	6.02	C8orf31
ENSG00000110375	6.02	UPK2
ENSG00000204179	6.02	PTPN20
ENSG00000236859	6.03	NIFK-AS1
ENSG00000196072	6.05	BLOC1S2
ENSG00000269139	6.06	
ENSG00000115474	6.07	KCNJ13
ENSG00000273449	6.08	
ENSG00000262979	6.09	
ENSG00000254616	6.09	
ENSG00000145736	6.10	GTF2H2
ENSG00000106336	6.10	FBXO24
ENSG00000243679	6.11	
ENSG00000279897	6.12	BIRC6-AS2

ENSG00000215630	6.13	
ENSG00000244270	6.13	
ENSG00000279334	6.13	
ENSG00000148832	6.13	PAOX
ENSG00000267543	6.15	
ENSG00000174871	6.17	CNIH2
ENSG00000100292	6.20	HMOX1
ENSG00000234005	6.20	
ENSG00000089847	6.22	ANKRD24
ENSG00000107105	6.22	ELAVL2
ENSG00000258034	6.23	
ENSG00000119440	6.23	
ENSG00000234975	6.24	
ENSG00000184260	6.24	HIST2H2AC
ENSG00000182600	6.25	C2orf82
ENSG00000164116	6.26	GUCY1A3
ENSG00000254602	6.28	
ENSG00000226088	6.30	
ENSG00000231663	6.30	LOC101927765
ENSG00000243402	6.30	
ENSG00000137574	6.31	TGS1
ENSG00000126243	6.31	LRFN3
ENSG00000110321	6.33	EIF4G2
ENSG00000203472	6.33	
ENSG00000126217	6.34	MCF2L
ENSG00000125775	6.34	SDCBP2
ENSG00000159197	6.35	KCNE2
ENSG00000154764	6.36	WNT7A
ENSG00000232119	6.37	MCTS1
ENSG00000104205	6.37	SGK3
ENSG00000184678	6.37	HIST2H2BE
ENSG00000213706	6.37	
ENSG00000137343	6.38	ATAT1
ENSG00000271161	6.39	
ENSG00000236986	6.39	
ENSG00000196167	6.40	
ENSG00000205683	6.41	DPF3
ENSG00000267082	6.41	LOC105372273
ENSG00000225951	6.43	
ENSG00000232346	6.43	
ENSG00000278887	6.44	
ENSG00000279228	6.44	

ENSG00000235655	6.45	
ENSG00000251432	6.46	LOC100507487
ENSG00000176927	6.47	EFCAB5
ENSG00000141655	6.48	TNFRSF11A
ENSG00000259485	6.49	
ENSG00000183305	6.49	MAGEA2B
ENSG00000260238	6.50	PMF1-BGLAP
ENSG00000249992	6.50	TMEM158
ENSG00000258644	6.53	SYNJ2BP-COX16
ENSG00000259308	6.55	
ENSG00000174151	6.56	CYB561D1
ENSG00000115170	6.56	ACVR1
ENSG00000143119	6.58	CD53
ENSG00000265646	6.58	
ENSG00000266947	6.58	
ENSG00000240401	6.59	
ENSG00000280145	6.59	
ENSG00000248576	6.60	
ENSG00000260235	6.60	
ENSG00000271869	6.60	
ENSG00000225806	6.61	
ENSG00000234174	6.62	
ENSG00000223722	6.64	
ENSG00000231245	6.64	
ENSG00000240224	6.64	UGT1A5
ENSG00000135114	6.68	OASL
ENSG00000129993	6.68	CBFA2T3
ENSG00000174945	6.68	AMZ1
ENSG00000226266	6.68	
ENSG00000267205	6.68	
ENSG00000216723	6.70	
ENSG00000237718	6.70	
ENSG00000172915	6.71	NBEA
ENSG00000288349	6.72	
ENSG00000258648	6.73	
ENSG00000153823	6.75	PID1
ENSG00000230495	6.75	
ENSG00000255364	6.75	
ENSG00000270528	6.75	
ENSG00000173926	6.76	MARCHF3
ENSG00000258654	6.76	
ENSG00000236886	6.77	

ENSG00000260630	6.78	SNAI3-AS1
ENSG00000261717	6.79	
ENSG00000278002	6.82	
ENSG00000151470	6.85	C4orf33
ENSG00000239969	6.87	
ENSG00000218757	6.88	
ENSG00000236264	6.88	
ENSG00000230216	6.90	
ENSG00000285619	6.90	
ENSG00000137819	6.91	PAQR5
ENSG00000273356	6.91	
ENSG00000243742	6.92	RPLP0P2
ENSG00000145979	6.92	TBC1D7-LOC100130357
ENSG00000140006	6.93	WDR89
ENSG00000237489	6.93	
ENSG00000271981	6.95	
ENSG00000184163	6.95	C1QTNF12
ENSG00000170088	6.96	TMEM192
ENSG00000101574	6.98	METTL4
ENSG00000248924	6.98	
ENSG00000128585	6.99	MKLN1
ENSG00000172346	6.99	CSDC2
ENSG00000230749	6.99	MEIS1-AS2
ENSG00000123600	7.02	METTL8
ENSG00000273747	7.03	
ENSG00000136521	7.03	NDUFB5
ENSG00000227218	7.05	
ENSG00000269680	7.06	
ENSG00000141756	7.09	FKBP10
ENSG00000225406	7.09	
ENSG00000258837	7.12	
ENSG00000260646	7.12	
ENSG00000238149	7.14	
ENSG00000270697	7.14	
ENSG00000267757	7.19	EML2-AS1
ENSG00000266541	7.19	
ENSG00000227430	7.24	
ENSG00000228201	7.24	
ENSG00000228436	7.24	LOC105378663
ENSG00000283255	7.24	
ENSG00000203618	7.26	GP1BB
ENSG00000179277	7.27	

ENSG00000270673	7.27	YTHDF3-AS1
ENSG00000204305	7.28	AGER
ENSG00000197780	7.29	TAF13
ENSG00000232859	7.31	LYRM9
ENSG00000251535	7.32	
ENSG00000239503	7.33	
ENSG00000281991	7.33	TMEM265
ENSG00000259468	7.34	
ENSG00000260082	7.34	
ENSG00000229873	7.36	OGFR-AS1
ENSG00000264932	7.37	
ENSG00000126233	7.37	SLURP1
ENSG00000229368	7.37	
ENSG00000205571	7.38	SMN2
ENSG00000060140	7.38	STYK1
ENSG00000233837	7.38	
ENSG00000184203	7.40	PPP1R2
ENSG00000224448	7.40	
ENSG00000249857	7.40	
ENSG00000276863	7.40	
ENSG00000134056	7.40	MRPS36
ENSG00000174514	7.40	MFSD4A
ENSG00000083750	7.44	RRAGB
ENSG00000234093	7.44	
ENSG00000278000	7.47	
ENSG00000113658	7.47	SMAD5
ENSG00000157617	7.48	C2CD2
ENSG00000113597	7.48	
ENSG00000128513	7.50	POT1
ENSG00000240875	7.52	LINC00886
ENSG00000243304	7.53	
ENSG00000198237	7.57	
ENSG00000227946	7.57	
ENSG00000167920	7.62	TMEM99
ENSG00000115827	7.64	DCAF17
ENSG00000214401	7.64	KANSL1-AS1
ENSG00000234353	7.64	
ENSG00000285625	7.66	
ENSG00000183793	7.68	NPIPA5
ENSG00000126861	7.68	LOC101927057
ENSG00000123609	7.68	NMI
ENSG00000167614	7.70	TTYH1

ENSG00000272719	7.70	
ENSG00000259429	7.74	
ENSG00000064787	7.75	BCAS1
ENSG00000152377	7.78	SPOCK1
ENSG00000278966	7.78	
ENSG00000273141	7.79	
ENSG00000251260	7.80	
ENSG00000226887	7.82	ERVMER34-1
ENSG00000113272	7.83	THG1L
ENSG00000148444	7.83	COMMD3
ENSG00000241852	7.83	C8orf58
ENSG00000233913	7.86	
ENSG00000227868	7.88	TEX46
ENSG00000120664	7.91	SPG20-AS1
ENSG00000180425	7.91	C11orf71
ENSG00000141380	7.94	SS18
ENSG00000236216	7.95	
ENSG00000226701	7.96	
ENSG00000260122	7.96	
ENSG00000249319	8.01	
ENSG00000163611	8.02	SPICE1
ENSG00000088053	8.05	GP6
ENSG00000151366	8.08	NDUFC2
ENSG00000259113	8.09	
ENSG00000132334	8.09	PTPRE
ENSG00000137185	8.10	ZSCAN9
ENSG00000171574	8.11	ZNF584
ENSG00000253948	8.12	LOC105375666
ENSG00000213302	8.14	
ENSG00000236325	8.14	
ENSG00000240541	8.15	TM4SF1-AS1
ENSG00000287799	8.15	
ENSG00000264265	8.16	LINC01925
ENSG00000261996	8.17	
ENSG00000165949	8.18	IFI27
ENSG00000176018	8.18	LYSMD3
ENSG00000260877	8.20	
ENSG00000224578	8.26	
ENSG00000149489	8.26	ROM1
ENSG00000223450	8.27	
ENSG00000234969	8.32	
ENSG00000164187	8.33	LMBRD2

ENSG00000236384	8.33	LINC00479
ENSG00000259656	8.33	
ENSG00000279509	8.33	
ENSG00000167759	8.33	KLK13
ENSG00000272906	8.33	
ENSG00000170006	8.35	TMEM154
ENSG00000244165	8.38	P2RY11
ENSG00000265254	8.39	
ENSG00000237063	8.40	
ENSG00000270427	8.41	
ENSG00000225187	8.43	
ENSG00000284095	8.43	
ENSG00000070501	8.45	POLB
ENSG00000178826	8.46	TMEM139
ENSG00000213023	8.48	SYT3
ENSG00000224886	8.49	
ENSG00000230593	8.49	
ENSG00000169271	8.50	HSPB3
ENSG00000120519	8.50	SLC10A7
ENSG00000226232	8.52	
ENSG00000165698	8.55	SPACA9
ENSG00000125910	8.56	S1PR4
ENSG00000270269	8.56	
ENSG00000235258	8.60	
ENSG00000229298	8.61	
ENSG00000114439	8.63	BBX
ENSG00000049768	8.64	FOXP3
ENSG00000258099	8.67	
ENSG00000260989	8.67	LOC105371046
ENSG00000166321	8.70	NUDT13
ENSG00000232888	8.71	
ENSG00000227258	8.75	SMIM2-AS1
ENSG00000283463	8.78	HSFX4
ENSG00000162006	8.88	
ENSG00000225630	8.89	
ENSG00000258366	8.90	
ENSG00000213760	8.90	ATP6V1G2
ENSG00000243749	8.95	TMEM35B
ENSG00000239483	8.97	
ENSG00000271949	8.97	
ENSG00000185442	9.00	FAM174B
ENSG00000165118	9.01	C9orf64

ENSG00000188647	9.09	PTAR1
ENSG00000198894	9.09	CIPC
ENSG00000260272	9.10	
ENSG00000258878	9.11	
ENSG00000233984	9.12	
ENSG00000256243	9.17	
ENSG00000224645	9.20	
ENSG00000223820	9.25	CFL1P1
ENSG00000269053	9.25	
ENSG00000241945	9.30	PWP2
ENSG00000081386	9.33	ZNF510
ENSG00000242067	9.33	
ENSG00000062725	9.34	APPBP2
ENSG00000180881	9.35	CAPS2
ENSG00000250315	9.38	
ENSG00000155975	9.38	VPS37A
ENSG00000115750	9.40	TAF1B
ENSG00000243697	9.41	
ENSG00000270995	9.42	
ENSG00000241907	9.45	
ENSG00000273138	9.45	
ENSG00000178445	9.51	GLDC
ENSG00000273248	9.53	
ENSG00000186001	9.54	LRCH3
ENSG00000276531	9.65	
ENSG00000236776	9.68	
ENSG00000225934	9.74	
ENSG00000248740	9.78	LINC02428
ENSG00000254681	9.79	
ENSG00000184434	9.81	LRRC19
ENSG00000265967	9.85	
ENSG00000258408	9.86	
ENSG00000262721	9.86	
ENSG00000285889	9.86	
ENSG00000280300	9.87	
ENSG00000226396	9.91	
ENSG00000232778	9.91	
ENSG00000166924	9.97	NYAP1
ENSG00000111450	9.98	STX2
ENSG00000176919	9.99	C8G
ENSG00000168899	10.00	VAMP5
ENSG00000008277	10.03	ADAM22

ENSG00000204072	10.04	
ENSG00000263155	10.05	
ENSG00000269145	10.08	
ENSG00000248302	10.10	
ENSG00000118363	10.16	SPCS2
ENSG00000240338	10.21	
ENSG00000262333	10.22	
ENSG00000225400	10.23	
ENSG00000240761	10.43	
ENSG00000261766	10.43	
ENSG00000276707	10.43	
ENSG00000159753	10.46	CARMIL2
ENSG00000224066	10.46	
ENSG00000251035	10.46	
ENSG00000234546	10.49	LINC01759
ENSG00000240096	10.50	
ENSG00000109189	10.57	USP46
ENSG00000174365	10.61	SNHG11
ENSG00000259065	10.64	
ENSG00000125841	10.67	NRSN2
ENSG00000213970	10.68	
ENSG00000234193	10.68	
ENSG00000165480	10.69	SKA3
ENSG00000126460	10.73	PRRG2
ENSG00000109911	10.74	ELP4
ENSG00000178372	10.75	CALML5
ENSG00000130822	10.75	PNCK
ENSG00000260537	10.77	
ENSG00000223652	10.79	
ENSG00000148153	10.80	INIP
ENSG00000234465	10.84	PINLYP
ENSG00000188428	10.86	BLOC1S5
ENSG00000182400	10.91	TRAPPC6B
ENSG00000213307	10.94	
ENSG00000197457	10.95	STMN3
ENSG00000163293	10.96	NIPAL1
ENSG00000270953	10.99	
ENSG00000286064	11.00	
ENSG00000214612	11.02	
ENSG00000119042	11.08	SATB2
ENSG00000256029	11.20	
ENSG00000203546	11.23	

ENSG00000230088	11.26	
ENSG00000185499	11.33	MUC1
ENSG00000269886	11.34	
ENSG00000279423	11.48	
ENSG00000286193	11.51	
ENSG00000275381	11.58	
ENSG00000184937	11.67	WT1
ENSG00000139865	11.73	TTC6
ENSG00000277599	11.77	
ENSG00000230896	11.77	
ENSG00000277959	11.83	
ENSG00000181524	11.84	
ENSG00000161243	11.86	FBXO27
ENSG00000279271	11.97	
ENSG00000082196	12.01	C1QTNF3
ENSG00000083454	12.05	P2RX5
ENSG00000200091	12.09	
ENSG00000264217	12.09	
ENSG00000197702	12.09	PARVA
ENSG00000227671	12.11	
ENSG00000279611	12.17	
ENSG00000233382	12.23	NKAPP1
ENSG00000267073	12.26	
ENSG00000163743	12.28	RCHY1
ENSG00000225548	12.31	
ENSG00000270462	12.33	
ENSG00000223505	12.36	
ENSG00000257906	12.36	
ENSG00000188015	12.44	S100A3
ENSG00000205116	12.63	TMEM88B
ENSG00000175548	12.75	ALG10B
ENSG00000236044	12.79	
ENSG00000279136	12.79	
ENSG00000121335	12.95	PRB2
ENSG00000260892	13.07	
ENSG00000196218	13.13	RYR1
ENSG00000228879	13.24	
ENSG00000231357	13.28	
ENSG00000236896	13.28	
ENSG00000270532	13.28	
ENSG00000269292	13.33	
ENSG00000229519	13.36	

ENSG00000222448	13.40	
ENSG00000260037	13.48	
ENSG00000225376	13.51	TMEM246-AS1
ENSG00000222460	13.56	
ENSG00000254924	13.56	
ENSG00000284829	13.56	
ENSG00000226564	13.72	
ENSG00000263412	13.73	
ENSG00000228727	13.90	SAPCD1
ENSG00000242602	13.90	
ENSG00000258186	13.91	
ENSG00000245556	13.95	SCAMP1-AS1
ENSG00000270149	14.01	
ENSG00000124116	14.06	WFDC3
ENSG00000128891	14.16	C15orf57
ENSG00000264339	14.26	
ENSG00000259118	14.28	LOC100506321
ENSG00000018610	14.31	CXorf56
ENSG00000265778	14.34	LOC101927989
ENSG00000230841	14.59	
ENSG00000260022	14.59	
ENSG00000216867	14.68	
ENSG00000250740	14.73	
ENSG00000134698	14.87	AGO4
ENSG00000160191	14.97	PDE9A
ENSG00000153485	15.04	TMEM251
ENSG00000239670	15.10	
ENSG00000152778	15.12	IFIT5
ENSG00000267795	15.16	SMIM22
ENSG00000224331	15.38	
ENSG00000249532	15.77	
ENSG00000232907	15.78	DLGAP4-AS1
ENSG00000138035	15.82	PNPT1
ENSG00000264924	15.88	
ENSG00000285160	16.12	
ENSG00000171204	16.28	TMEM126B
ENSG00000104863	16.55	LIN7B
ENSG00000269392	16.69	
ENSG00000204397	16.97	CARD16
ENSG00000258824	17.03	
ENSG00000219355	17.07	
ENSG00000224251	17.16	

ENSG00000257078	17.27	
ENSG00000239544	17.49	
ENSG00000276698	17.59	
ENSG00000271858	17.60	
ENSG00000259087	17.64	
ENSG00000279332	17.79	
ENSG00000182648	18.06	LINC01006
ENSG00000268903	18.22	
ENSG00000272379	18.26	
ENSG00000232442	18.65	MHENCR
ENSG00000255100	18.74	LOC101928837
ENSG00000255262	18.90	
ENSG00000213178	18.95	
ENSG00000240005	19.02	
ENSG00000256338	19.06	
ENSG00000163818	19.26	LZTFL1
ENSG00000240006	19.31	
ENSG00000215444	19.46	
ENSG00000263396	19.92	
ENSG00000144785	20.00	
ENSG00000229212	20.04	LOC101929479
ENSG00000257433	20.09	LOC105369748
ENSG00000281348	20.48	
ENSG00000266869	20.57	
ENSG00000228728	20.89	
ENSG00000235636	21.38	
ENSG00000279370	21.58	
ENSG00000234367	21.81	
ENSG00000217078	22.17	
ENSG00000254780	22.30	
ENSG00000259018	22.30	
ENSG00000237015	22.33	
ENSG00000243870	22.68	
ENSG00000277900	23.06	
ENSG00000277209	23.41	RPPH1
ENSG00000260608	23.68	
ENSG00000243519	24.18	
ENSG00000223975	24.28	
ENSG00000244296	24.71	
ENSG00000151883	24.99	PARP8
ENSG00000249791	24.99	
ENSG00000278876	25.58	

ENSG00000255198	26.55	SNHG9
ENSG00000241693	27.04	
ENSG00000226465	27.13	
ENSG00000134297	27.22	PLEKHA8P1
ENSG00000238072	28.11	
ENSG00000266538	28.14	
ENSG00000228974	29.59	
ENSG00000258300	29.99	
ENSG00000172062	30.50	SMN1
ENSG00000229046	30.77	
ENSG00000252233	31.18	
ENSG00000234753	32.77	FOXP4-AS1
ENSG00000267740	33.18	
ENSG00000256804	33.38	
ENSG00000263321	35.12	
ENSG00000240370	35.82	RPL13P5
ENSG00000263301	36.28	
ENSG00000256192	38.13	
ENSG00000169490	38.92	TM2D2
ENSG00000257403	39.50	
ENSG00000168273	41.90	SMIM4
ENSG00000277371	46.92	
ENSG00000275413	47.84	
ENSG00000100427	50.33	MLC1
ENSG00000286874	52.36	
ENSG00000258788	55.58	
ENSG00000268739	55.58	
ENSG00000265519	61.00	
ENSG00000222069	69.48	
ENSG00000267203	73.13	
ENSG00000284613	95.36	
ENSG00000271511	96.54	
ENSG00000271267	113.55	
ENSG00000224543	117.19	
ENSG00000143429	118.17	
ENSG00000227097	123.38	
ENSG00000288626	124.60	
ENSG00000277027	131.05	
ENSG00000223544	132.05	
ENSG00000272196	182.76	HIST2H2AA4
ENSG00000285723	186.33	
ENSG00000235681	196.45	

ENSG00000259813	226.25
ENSG00000272114	246.07
ENSG00000200693	295.04
ENSG00000274630	831.55

Supplementary Table 5. Potential miRNA target sites in the LINC02154 sequence

miRNA	Seed position (hg19)
miR-7/7ab	chrX:13321484
miR-144	chrX:13284192
miR-145	chrX:13305636
miR-155	chrX:13305648
miR-200bc/429/548a	chrX:13285000
miR-203	chrX:13284238
miR-217	chrX:13285003
miR-219-5p/508/508-3p/4782-3p	chrX:13284920
miR-22/22-3p	chrX:13285364
miR-31	chrX:13321516
miR-124/124ab/506	chrX:13285185
miR-34ac/34bc-5p/449abc/449c-5p	chrX:13284723
miR-425/425-5p/489	chrX:13284460
miR-455-5p	chrX:13284712
miR-128/128ab	chrX:13284693

Supplementary Table 6. Correlations between levels of LINC02154 expression and those of miRNAs

miRNA	Correlation coefficient	P
hsa-mir-7-1	-0.370	1.32E-06
hsa-mir-144	0.067	4.00E-01
hsa-mir-145	0.125	1.15E-01
hsa-mir-155	0.025	7.51E-01
hsa-mir-200b	-0.424	2.07E-08
hsa-mir-200c	-0.317	4.21E-05
hsa-mir-429	-0.356	3.56E-06
hsa-mir-203a	0.128	1.06E-01
hsa-mir-217	-0.164	3.81E-02
hsa-mir-219a	-0.235	2.69E-03
hsa-mir-508	-0.110	1.67E-01
hsa-mir-4782	-0.239	2.23E-03
hsa-mir-22	0.370	1.36E-06
hsa-mir-31	0.156	4.80E-02
hsa-mir-124	0.072	3.63E-01
hsa-mir-506	-0.028	7.21E-01
hsa-mir-34a	-0.168	3.32E-02
hsa-mir-34b	0.248	1.48E-03
hsa-mir-34c	0.208	8.20E-03
hsa-mir-449a	-0.056	4.80E-01
hsa-mir-449b	-0.045	5.73E-01
hsa-mir-449c	-0.062	4.35E-01
hsa-mir-425	-0.224	4.36E-03
hsa-mir-489	-0.198	1.18E-02
hsa-mir-455	0.158	4.51E-02
hsa-mir-128	-0.105	1.84E-01

Supplementary Table 7. Proteins detected with RNA pulldown-mass spectrometry analysis

Accession	Gene Symbol	Protein name	Score Sequest HT	Coverage (%)
Q5T749	KPRP	Keratinocyte proline-rich protein [OS=Homo sapiens]	21.2	20
Q02413	DSG1	Desmoglein-1 [OS=Homo sapiens]	14.42	11
P14923	JUP	Junction plakoglobin [OS=Homo sapiens]	25.72	15
P01040	CSTA	Cystatin-A [OS=Homo sapiens]	17.51	47
P04406	GAPDH	Glyceraldehyde-3-phosphate dehydrogenase [OS=Homo sapiens]	16.21	20
P29508	SERPINB3	Serpin B3 [OS=Homo sapiens]	13.2	18
P81605	DCD	Dermcidin [OS=Homo sapiens]	12.91	33
Q6KB66	KRT80	Keratin, type II cytoskeletal 80 [OS=Homo sapiens]	11.37	10
Q8N1N4	KRT78	Keratin, type II cytoskeletal 78 [OS=Homo sapiens]	12.01	10
Q08554	DSC1	Desmocollin-1 [OS=Homo sapiens]	8.54	4
Q08188	TGM3	Protein-glutamine gamma-glutamyltransferase E [OS=Homo sapiens]	7.08	4
P12273	PIP	Prolactin-inducible protein [OS=Homo sapiens]	5.69	18
P07355	ANXA2	Annexin A2 [OS=Homo sapiens]	2.08	7
P31949	S100A11	Protein S100-A11 [OS=Homo sapiens]	3.08	15
Q8WVV4	POF1B	Protein POF1B [OS=Homo sapiens]	1.84	2
P02790	HPX	Hemopexin [OS=Homo sapiens]	2.14	4
P60174	TPI1	Triosephosphate isomerase [OS=Homo sapiens]	1.91	5
Q6UWP8	SBSN	Suprabasin [OS=Homo sapiens]	2	2
Q13835	PKP1	Plakophilin-1 [OS=Homo sapiens]	1.71	1
A0A0A0MS14	IGHV1-45	Immunoglobulin heavy variable 1-45 [OS=Homo sapiens]	4.44	9
Q9NVJ2	ARL8B	ADP-ribosylation factor-like protein 8B [OS=Homo sapiens]	0	16
P14625	HSP90B1	Endoplasmin [OS=Homo sapiens]	2.43	1
O95715	CXCL14	C-X-C motif chemokine 14 [OS=Homo sapiens]	0	14
P59665	DEFA1; DEFA1B	Neutrophil defensin 1 [OS=Homo sapiens]	1.75	10
P35579	MYH9	Myosin-9 [OS=Homo sapiens]	0	1
P22528	SPRR1B	Cornifin-B [OS=Homo sapiens]	0	9
P27482	CALML3	Calmodulin-like protein 3 [OS=Homo sapiens]	0	11
P22735	TGM1	Protein-glutamine gamma-glutamyltransferase K [OS=Homo sapiens]	1.92	2
P16150	SPN	Leukosialin [OS=Homo sapiens]	0	7
Q86XP0	PLA2G4D	Cytosolic phospholipase A2 delta [OS=Homo sapiens]	0	1
P22532	SPRR2D	Small proline-rich protein 2D [OS=Homo sapiens]	2.19	13
P11021	HSPA5	Endoplasmic reticulum chaperone BiP [OS=Homo sapiens]	0	2
